# Ancient and modern DNA track temporal and spatial population dynamics in the European fallow deer since the Eemian interglacial

**DOI:** 10.1038/s41598-023-48112-6

**Published:** 2024-02-12

**Authors:** K. H. Baker, H. W. I. Gray, A. M. Lister, N. Spassov, A. J. Welch, K. Trantalidou, B. De Cupere, E. Bonillas, M. De Jong, C. Çakırlar, N. Sykes, A. R. Hoelzel

**Affiliations:** 1 Department of Biosciences, Durham University, South Road, Durham, DH1 3LE UK; 2 Natural History Museum, London, SW7 5BD UK; 3 National Museum of Natural History at the Bulgarian Academy of Sciences, Sofia, Bulgaria; 4 Hellenic Ministry of Culture, 5 Themistokleous Str., 10677 Athens, Greece; 5 OD Earth and History of Life, Royal Belgian Institute of Natural Sciences, Brussels, Belgium; 6 Groningen Institute of Archaeology, University of Groningen, Poststraat 6, 9712 NL Groningen, The Netherlands; 7 Department of Archaeology and History, University of Exeter, Exeter, UK

**Keywords:** Ecology, Evolution

## Abstract

Anthropogenic factors have impacted the diversity and evolutionary trajectory of various species. This can be through factors such as pressure on population size or range, habitat fragmentation, or extensive manipulation and translocation. Here we use time-calibrated data to better understand the pattern and processes of evolution in the heavily manipulated European fallow deer (*Dama dama*). During the Pleistocene, fallow deer had a broad distribution across Europe and were found as far north as Britain during the Eemian interglacial. The last glacial period saw fallow deer retreat to southern refugia and they did not disperse north afterwards. Their recolonisation was mediated by people and, from northern Europe and the British Isles, fallow deer were transported around the world. We use ancient and modern mitochondrial DNA (mtDNA) and mitogenomic data from Eemian Britain to assess the pattern of change in distribution and lineage structure across Europe over time. We find founder effects and mixed lineages in the northern populations, and stability over time for populations in southern Europe. The Eemian sample was most similar to a lineage currently in Italy, suggesting an early establishment of the relevant refuge. We consider the implications for the integration of anthropogenic and natural processes towards a better understanding of the evolution of fallow deer in Europe.

## Introduction

Patterns of population connectivity and structure are typically assessed at a point in time, and inference about biogeography and evolutionary process can be derived from this. However, populations are dynamic, as are the boundaries and factors that influence migration and dispersal. An important factor is the contribution of human influence on species distributions through exploitation and translocation. Here we focus on the fallow deer (*Dama dama*) a species subject both to processes associated with the glacial cycles^1^ and extensive human translocations^2^. We use ancient and modern DNA to assess the influence of historical dynamics on the modern population structure and patterns of diversity.

Palaeontological data indicate that fallow deer were widely distributed across continental Europe and the British Isles during the late Middle to Late Pleistocene interglacials, at least between Marine Isotope Stage 11 (MIS 11; 424–374 ka) and 5e (Eemian, 130–115 ka)^3,4^. Morphological studies have indicated some variation between periods. For instance, antlers from the Eemian of Germany were considered distinctive and named as a separate subspecies, *D. dama geiselana*^5^, later even elevated to species status as *D. geiselana*^6^. Fallow deer had became extinct in northern and central latitudes of Europe by the last glacial period (MIS 4-2, ca. 72–12 ka) and, unlike numerous other European mammals (e.g.^7^), this species failed to repopulate the north from any southern refugia during the Holocene.

Today, European fallow deer (*Dama dama*) are one of the world’s most widely distributed cervids due to their human-mediated translocation over the last 10 kya. They are now established across countries in Eurasia, Africa, the Americas and Oceania^8^. The timing and circumstances of their global spread have been investigated through numerous regional investigations^9–15^. These studies have raised questions about the fallow deer’s natural post-glacial range, and proposed refugial populations have included southern mainland Italy, Sicily, the southern Balkan peninsula and Anatolia (see^13,15–17^).

Baker et al.^2^ complement genetic data with osteological and isotopic data for ancient and modern specimens from Europe and beyond to assess the species’ origins and spread. They propose that fallow deer survived the Pleistocene/Holocene boundary in two areas. The first is Anatolia, where fallow deer are represented in archaeological assemblages dating from the Late Pleistocene to Early Holocene^13^. These populations diminished through time and the only surviving native herd exists in the Düzlerçamı wildlife reserve in the Antalya Province (see^18^). Secondly, the Balkans (Greece and Bulgaria) supported an extinct autochthonous population that is morphologically and genetically distinct from the Turkish fallow deer^2,15,17^. The original Balkan population was gone by Roman times, or possibly early Middle Ages. The fallow deer there now are translocated from Germany and Austria^15^.

While there is variation by region, in general the European Neolithic ran from about 9000–4000 years ago, the Bronze Age overlapped to some extent (~ 5200–3200 years ago), the Roman period ran from ~ 2000 to ~ 1600 years ago, and the Byzantine period from ~ 1700 to 570 years ago. Zooarchaeological data suggest that fallow deer were taken from the Balkans to Mediterranean islands during the late Neolithic and Bronze Age, and that in the Roman period they became established across Italy, Iberia, northern Europe and into Britain. Following their post-Roman extinction in northern Europe and Britain, these records imply that a new population (this time brought from Anatolia) was reintroduced to northern Europe and Britain from where they were transported around the world (see review^2^).

Genetic studies had earlier cast doubt on the proposition that modern populations derived from a single surviving Turkish population^1,19^, which should result in a ‘star-burst’ pattern of differentiation (little phylogenetic resolution). Instead, these studies revealed phylogenies with separate lineages, including a complex pattern for translocated lineages in the north. Nuclear microsatellite data, from an extensive sample (N = 364) across the current species range^1^ showed that samples from Iberia (Spain and Portugal), Italy, Turkey (Anatolia) and the Greek island of Rhodes clustered independently from each other, and genetic distances were much greater among these regions than among different locations within them, even though these within-region sites were sometimes geographically distant^1^.

Here we focus on the spatio-temporal patterns revealed by time-calibrated data and phylogenies, including data from the Eemian interglacial to help us to determine the timing and location of the earliest refugium. These data allow us to better understand the way natural and anthropogenic processes interact to establish modern distributions and diversity for species such as fallow deer that have been extensively manipulated and translocated by humans and also impacted by environmental change.

## Methods and materials

### Sampling

**Figure 1:**
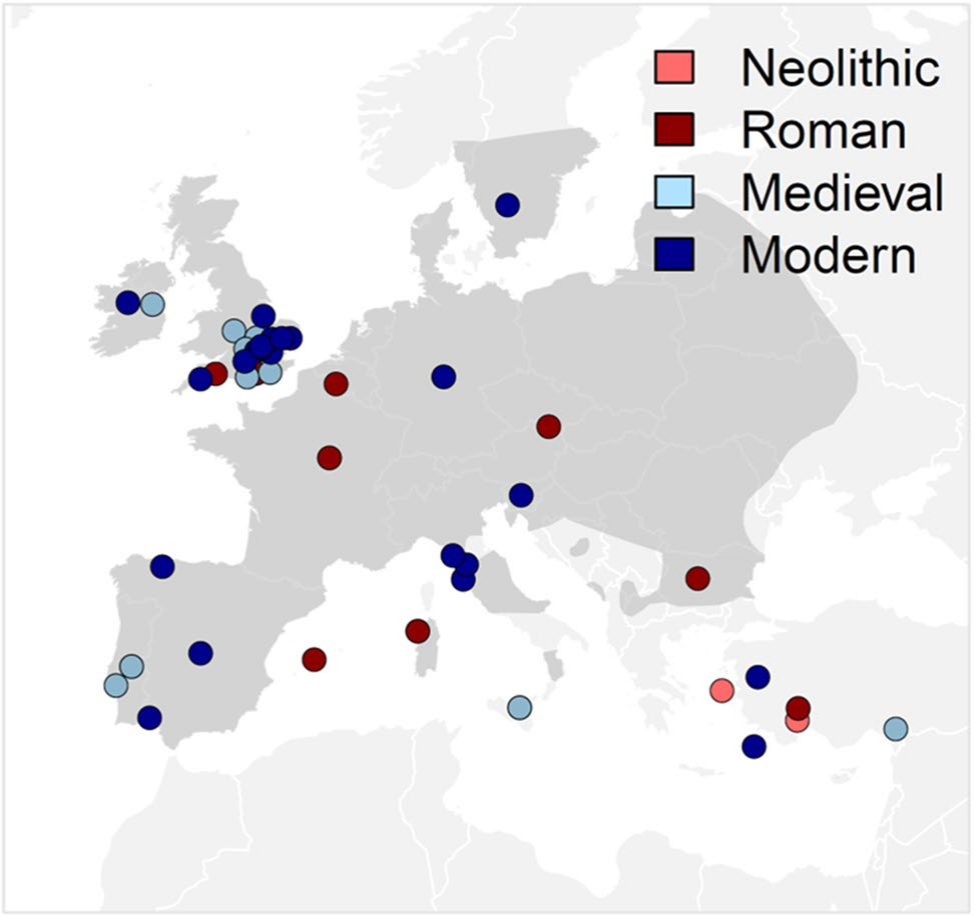
Map figure showing the distribution of sample sites by location and age in the European region. Translocated samples from Sweden, Barbuda and Canada are not shown. Darker grey shading is the approximate modern distribution (after https://www.iucnredlist.org/species/42188/10656554).

This study is based on 401 samples (182 of which are ancient and 219 of which are modern) collected across the *D. dama* species range with a focus on Europe (Fig. 1; see Table S1 for detailed locations). Southern Europe is represented by samples from Bulgaria, Turkey, Italy, Greece and Spain. Northern and central mainland Europe are represented by Germany, Hungary, France and Belgium. Additional northern samples are from the UK, Ireland and Sweeden. Samples from animals translocated to other continents are from Barbuda and Canada (Table S1). Two samples are from the Joint Mitnor Cave site in Devon, England date to ~ 125 ka (Eemian Interglacial). Full sample information including mtDNA haplotype details are given in the electronic supplementary material, Table S1.

### Ancient DNA extraction

Bone samples were surface sanded (to ensure removal of surface contaminating DNA) and a small segment of bone was then cut (~ 1 cm^3^) using a variable speed Dremmel™ drill. Bone powder was obtained from cut segments by placing in a mikrodismembrator (Sartorius). 50 mg of this bone powder was then incubated overnight at 50 °C with 1 mL of extraction buffer (0.5 M EDTA at pH 8.0, 0.5% SDS and 0.5 mg/mL proteinase K) in a 1.5 mL tube. DNA was extracted using a QIAquick purification kit™ (Qiagen) and stored at − 20 °C.

Precautions to avoid contamination were taken during every stage of aDNA extraction and PCR set up, which took place in a separate laboratory dedicated to aDNA research, free from contemporary DNA or PCR products. No laboratory materials or clothing were transferred from the post amplification rooms to the ancient laboratory. All equipment, plasticware and DNase & RNase-free deionized sterile water was autoclaved in a dedicated aDNA autoclave. All work surfaces and equipment were thoroughly cleaned with 10% bleach (sodium hypochlorite) followed by 70% ethanol. Surfaces, equipment and solutions were also routinely exposed to UV light for at least 10 min. All extractions and PCR work were carried out in class II PCR hoods. Negative extraction and PCR controls (1 sample in every 5) were both included to detect potential contamination in reagents and cross-contamination between samples. 30% of samples were replicated by extracting twice from independent samples of the same bone.

### Ancient mitochondrial D-loop sequencing and cloning

For ancient samples a 532 base pair subsection from the 5′ end of the mitochondrial control region (CR) was PCR amplified using a combination of the overlapping primers listed in Table S2. PCR Reactions (25 µl) contained 2 µl of DNA extract, 22.6 µl Multiplex *PCR* Kit (Qiagen) and 0.2 µM of each primer. Thermal cycling conditions were as follows: an initial denaturation of 15 min at 95 °C followed by 45 cycles of 30 s at 95 °C, 90 s annealing, 45 s at 72 °C followed by a final extension for 30 min at 60 °C. Negative controls (no DNA) were used for all PCR runs. Column-purified PCR products were Sanger sequenced at DBS Genomics, Durham University. Samples showing any sequencing ambiguities were replicated for all steps.

To help assess and quantify post-mortem damage and exogenous DNA content, for 6 ancient samples the PCR product was cloned into the pGEM-T Easy vector (Invitrogen) and transformed into JM109 competent cells (by heat-shock for 45–50 s). After blue/ white selection, positive colonies were miniprepped and the insert DNA amplified by PCR using T7 and Sp6 primers. A minimum of 6 clones were sequenced on an ABI automated sequencer from all 6 samples (chosen at random) to check for potential contamination or incorrect base calls. During cloning and sequencing, no evidence of nuclear copies was detected.

### Mitogenome capture and sequencing

A subset of ten modern samples (see below), together with two bone samples from the Joint Mitnor Cave in Devon, England (dated to the Eemian interglacial period) were subject to whole mitogenome hybridisation capture in the ancient DNA lab. First, library construction was performed based on the protocols of^20,21^. Modern and ancient samples were treated differently. For ancient samples with degraded DNA no sheering was required. Because miscoding errors can be generated by cytosine deamination, an end-repair step was included to remove uracil using 10 × NEB Buffer 2, T4 Polynucleotide Kinase (10U/ul), and USER enzyme (uracil DNA glycosylase, 1 U/ul)^20^. Initial steps of library preparation were carried out in a dedicated aDNA lab. Briefly, adapter sequences were ligated and MyOne C1 Streptavidin beads were used during washes to remove extra adapters not attached to the DNA as suggested by Knapp et al.^21^. After bead cleaning adapter fill-in was done using BWT buffer and Bst polymerase (8 U/ul). The initial amplification for ancient samples consisted of adding specific primer P7 (index) to each sample separately (universal P7 for the modern samples), followed by the addition of a “master mix” containing a universal primer P5, 10 × Thermopol buffer, dNTP mix, and AmpliTaq Gold DNA Polymerase and PCR amplified (94 °C for 8 min, then 12 cycles of 94 °C for 30 s, 58 °C for 30 s, 72 °C for 1 min, followed by 72 °C for 7 min). After cleanup on Qiaquick columns, captured DNA was quantified via qPCR and then amplified using Phusion Taq, and the P5 and P7 universal primers.

Capture enrichment was conducted using RNA baits in a kit produced by myBaits (ArborBioscience) designed from the *D. dama* mitogenome reference sequence (NC_020700.1). The capture protocol followed manufacturers recommendations. Briefly, capture baits were added in 0.21 ul per 16.5 ul reaction, hybridized over 2 days at 65 °C and then 55 °C. A blocker mix was used, which was comprised of human Cot-1, salmon sperm DNA and myBaits adapters. After washing and bead cleaning the captured DNA was taken up in 25 ul and quantified by qPCR before amplifying using universal Illumina adaptors to increase concentration. For the ancient samples a second round of capture was undertaken to facilitate greater target recovery (after^22^). Enriched libraries were sequenced paired end, 2 × 100 bp on an Illumina HiSeq 2500.

The raw fastq files were filtered (Trimmomatic) to remove adapter sequences. Demultiplexing was done in Stacks v1 generating FASTQ files^23^. Bowtie 2 was used to align the sequences against the reference mitogenome (Genbank accession: JN632629.1) and generate SAM (Sequence Alignment Map) files^24^. The SAM file was converted to BAM format using the software SAMTOOLS for alignment in Geneious (8.1.7). Segments with less than 4X coverage were filtered out.

### Modern sample extraction and sequencing for the mtDNA control region

DNA from modern samples (see Table 1) was extracted and amplified following the methods as described in^1^. For this study all sequences were trimmed to the comparative 532 bp, consensus region amplified in ancient samples.

**Table 1:** Diversity and demographic metrics for each population.

Population	N	*h*	π	Tajima’s D	Fu’s Fs
Ancient UK and Ireland	118	0.734 ± 0.037	0.0049 ± 0.0029	− 1.374 (*p* = 0.046)	− 9.378 (*p* = 0.004)
Modern UK and Ireland	71	0.862 ± 0.025	0.0046 ± 0.0028	− 0.594 (*p* = 0.3)	− 3.752 (*p* = 0.073)
Ancient northern Europe	12	0.667 ± 0.141	0.006 ± 0.0038	− 0.981 (*p* = 0.17)	0.807 (*p* = 0.68)
Modern northern Europe	24	0.859 ± 0.052	0.0052 ± 0.0032	− 1.384 (*p* = 0.073)	− 2.658 (*p* = 0.087)
Ancient Iberia	4	1.0 ± 0.177	0.0053 ± 0.0042	− 0.213 (*p* = 0.61)	− 1.414 (*p* = 0.062)
Modern Iberia	36	0.503 ± 0.093	0.0034 ± 0.0023	− 0.595 (*p* = 0.29)	1.138 (*p* = 0.75)
Ancient Italy	18	0.641 ± 0.13	0.0019 ± 0.0016	− 2.001 (*p* = 0.008)	− 5.426 (*p* = 0.0)
Modern Italy	26	0.582 ± 0.059	0.01 ± 0.0056	2.069 (*p* = 0.99)	6.764 (*p* = 0.977)
Anatolia	23	0.395 ± 0.128	0.0028 ± 0.002	− 1.621 (*p* = 0.044)	− 1.028 (*p* = 0.25)
Modern Turkey	20	0.1 ± 0.088	0.0004 ± 0.0006	− 1.512 (*p* = 0.046)	− 0.025 (*p* = 0.26)

### Genetic diversity and structure

MtDNA control region (CR) sequences were aligned using the MUSCLE algorithm^25^ as implemented in Geneious v. R6 (http://www.geneious.com)^26^. The program DnaSP v. 10.4.9^27^ was used to calculate mtDNA polymorphism estimated as haplotypic diversity, (*h*)^28^, nucleotide diversity (π)^29^, and average pairwise nucleotide divergence (*k*). To assess the level of genetic differentiation and demographic trends among populations, *h*, π, Tajima’s D and Fu’s Fs were calculated for CR sequences using ARLEQUIN^30^ v. 3.5. Significance for neutrality tests was assessed using 1000 permutations.

CR phylogenies were estimated using three datasets: ancient-only (46 haplotypes), modern-only (33 haplotypes), and a combined ancient-and-modern dataset (72 haplotypes). All analyses utilized 594 bp of mtDNA CR sequence and a database sequence of Persian fallow deer (*Dama mesopotamica*) was used as an outgroup (accession: AF291896.1). MrBayes^31^ v. 3.2.6 was implemented using the CIPRES Scientific Gateway^32^ v. 3.3 to estimate Bayesian phylogenies for each dataset. Four independent Markov chains (three heated) were run over 30,000,000 MCMC generations, with a 10% burn-in, and a sampling frequency of 1000 generations. Each analysis was run twice to confirm convergence. Diagnostic outputs such as effective sample size (ESS) and potential scale reduction factor (PSRF) values were consulted to confirm convergence had been achieved and that sufficient iterations had been implemented. All ESS values were greater than 100 and all PSRF values approached one. The best model of evolution was inferred using PartitionFinder^33,34^ v. 1.0.1 considering all the evolutionary models available to MrBayes. For all datasets, the HKY model^35^, with gamma distributed rate variation among sites (G), was chosen, based on the Bayesian information criterion (BIC) metric.

Mitogenome analyses were based on modern mitogenomes from ten samples edited down to match the regions amplified for the best Eemian sample. Two Bayesian phylogenies were constructed considering (1) a dataset partitioned according to data blocks (matching segments successfully sequenced for the Eemian sample) and, (2) an unpartitioned dataset. The best partitioning schemes and models of evolution were inferred using the BIC metric in PartitionFinder and Jmodeltest^36^ for the partitioned (see Table S3) and unpartitioned (GTR + I) datasets, respectively. Trees were inferred in MrBayes as outlined above. The mitogenome trees included red deer (*Cervus elaphus*; accession MF872248.1) and giant deer (*Megaloceros giganteus*; accession MW802577.1) as outgroups, and included database mitogenome sequences for *Dama dama dama* (JN632629.1) and *Dama dama mesopotamica* (JN632630.1).

**Figure 2:**
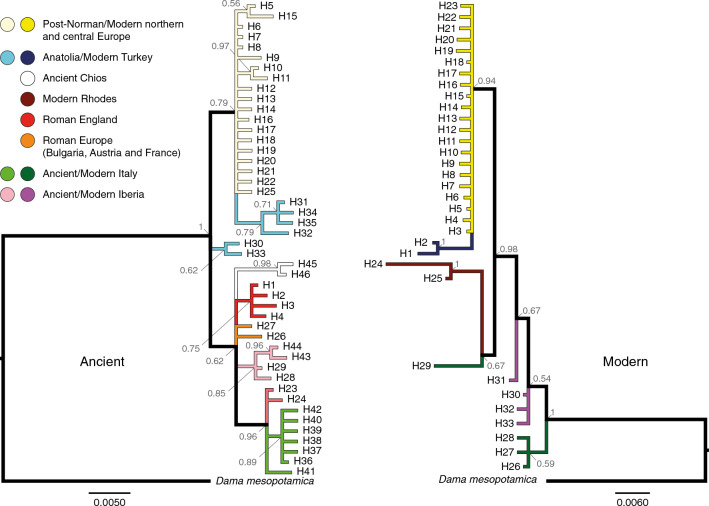
Bayesian inferred phylogenies for ancient-only and modern-only *D. dama* mtDNA control region haplotypes, generated using MrBayes. The trees illustrate a similar topology that persists over time. Posterior probability support values are presented at nodes. Colours refer to collection localities and time periods. Scale bar = substitutions per site.

**Figure 3:**
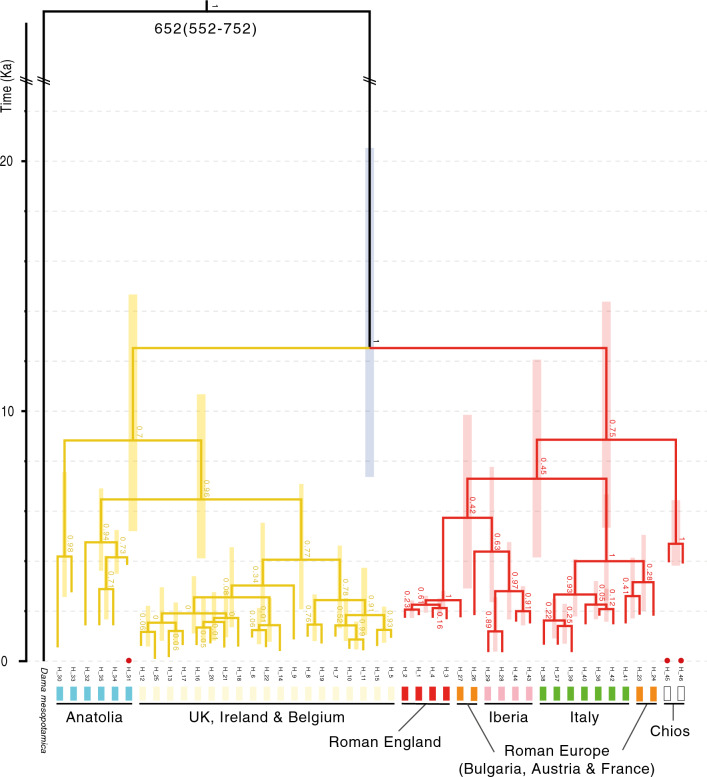
Time-calibrated tree using an ancient-only *D. dama* mtDNA control region dataset. Generated in BEAST utilising the strict-clock and exponential growth demographic model. Bars correspond to 95% highest posterior density (HPD) of divergence estimates at each node. Colour bars at terminal nodes correspond to localities and time periods given in Fig. 2. Posterior probability support values are given at respective nodes. The ‘//’ symbol denotes a shortening of branch length for the outgroup. The values given at the root node are the divergence time estimate (Ka) followed by the 95% HPD in parentheses. The oldest haplotypes (from the Neolithic) are indicated with a red dot. Red and yellow colours show well-supported lineages.

Divergence dates were estimated from the ancient-only and combined CR datasets and for the (unpartitioned) mitogenome dataset using BEAST^37,38^ v.1.10.4. The best substitution models for these datasets were identified using PartitionFinder. For the CR datasets, the *D. mesopotamica* haplotype was assigned as the outgroup by constraining the ingroup (*D. dama* haplotypes) to monophyly (tree prior, only). In addition, two calibration nodes were constrained to monophyly, representing (1) the divergence between *D. mesopotamica* and *D. dama* based on the fossil record (see review)^6^ ~ 700,000 YBP (priors: normal distribution; mean = 700,000; stdev = 50,000), and (2) the divergence of Roman-English *D. dama* populations (haplotypes H-1–H-4) from a haplotype (H-27) found in Roman-Europe (~ 2000 YBP; prior: lognormal distribution; mean (real space) = 2000; stdev (real space) = 200). This is based on the timing of the known first translocation of fallow deer into Britain^2^, and the fact that H-27 is within the same terminal lineage as the Roman-English haplotypes (Figs. 2 and 3). The oldest dates for each haplotype, estimated based on radiocarbon dating and sedimentary position, were also incorporated in the model (see Table S4). Modern haplotypes were assigned a fixed prior of 0. Tip dates were sampled using individual priors (normal distributions; see Table S4). The initial tree was generated at random, and a Yule-branching model was applied for the tree prior. Two demographic models (Constant Size and Exponential Growth) were tested under the strict clock and the lognormal uncorrelated relaxed clock models: four models were compared for each CR dataset (see Table S4).

For the mitogenome tree, *C. elaphus* was assigned as the outgroup by constraining all other sequences to monophyly (tree only prior). Two further time-calibrated nodes were constrained based on (1) divergence between *D. mesopotamica* and *D. dama*, as described above (prior: normal distribution; mean = 659,000; stdev = 50,000) and (2) divergence of the Rhodes lineage (prior: normal distribution; mean = 8,000; stdev = 500; based on suggested founding in the Neolithic). The tip dates for the Eemian haplotype (prior: normal distribution; mean = 130,000; stdev = 10,000) and the giant deer (prior: normal distribution; mean = 20,000; stdev = 10,000) were also included. All remaining modern haplotypes were assigned a fixed prior of ‘0’ and tip-date sampling was applied. A relaxed mutation rate prior was incorporated using a mitogenome mutation rate (1.3 × 10^–8^ per bp per year) based on analysis in^39^. Fourteen models were investigated considering different demographic models under various clock models (see Table S3 for further details).

MCMC analyses were run with 60,000,000 iterations and a 10% burn-in applied. Sampling was conducted every 1000 iterations. Three independent runs were performed to check for convergence on similar posterior distributions, as examined in TRACER^40^ v.1.7.2. Resulting ESS values for all parameters were above 200, suggesting an appropriate number of iterations had been performed. Outputs were combined in LogCombiner^37^ v. 1.10.4 and the combined trees were transferred to TreeAnnotator^37^ v.1.10.4 where an annotated maximum clade credibility tree with median node ‘heights’ was generated.

Models (see Table S3) were compared using stepping-stone sampling^41,42^. For each model, three independent runs were performed using 100 power-posteriors over 1,000,000 iterations. Stepping-stone sampling was used to estimate the log marginal likelihoods for each run and from combined independent runs^43^. Log Bayes factors were generated from the log marginal likelihoods for model comparison. To confirm convergence on log marginal likelihoods, runs were carried out again for twice the number of iterations (2,000,000).

## Results

There were a total of 72 CR haplotypes among the 402 samples, 46 of which were from the 183 ancient samples. Summary data on diversity per population and neutrality test statistics are given in Table 1. Ancient and modern sample sets from a given location are considered both together and separately. Data for Roman to Medieval Iberia are included for completeness, but the sample size is too small (N = 4) to allow for strong inference for population comparisons. The southern locations (both ancient and modern) each had relatively low diversity compared to the admixed populations (mixture of interbreeding natal populations) in the north. The strongest signals for population expansion based on tests that compare against random (neutral) expectations (Tajima’s D and Fu’s Fs) were in ancient UK/Ireland, and ancient Italy (Table 1). Positive values for both Tajima’s D and Fu’s Fs in modern Italy could suggest population contraction (Table 1). Comparing modern and ancient Bayesian phylogenies shows broad correspondence, though the regions sampled during different periods are not evenly represented (Fig. 2). There is clear clustering of both ancient and modern central and northern mainland Europe with ancient and modern Anatolia. Italy (including Roman Europe) and Iberia (ancient and modern) cluster separately. Modern Rhodes shares a lineage with a modern Italian haplotype. The general topology is retained when both modern and ancient samples are included in the same tree (Figure S1).

**Figure 4:**
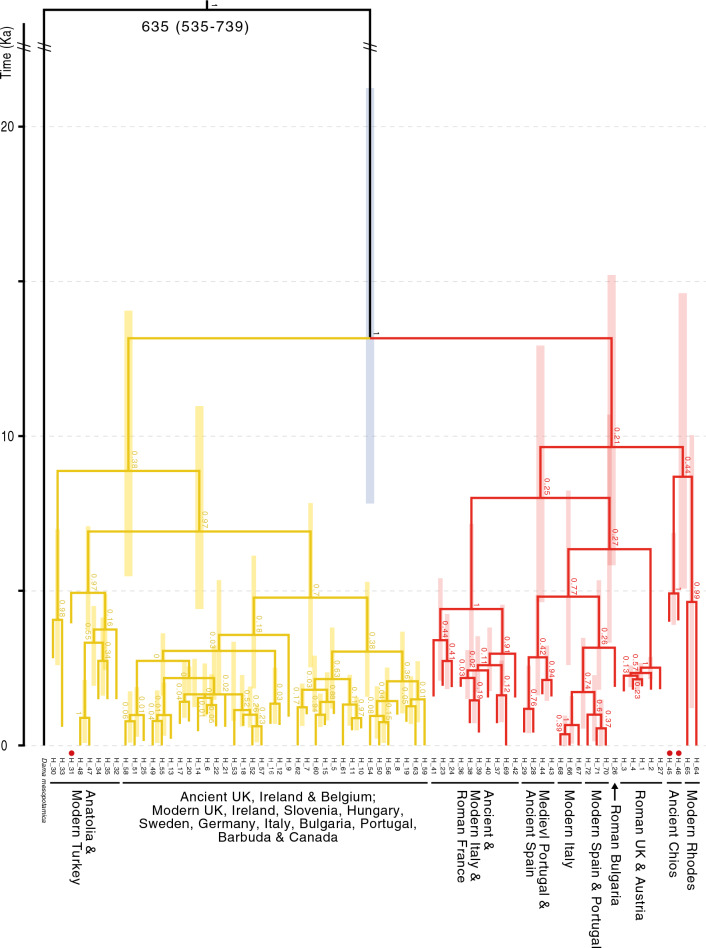
Time-calibrated tree using the ancient-and-modern *D. dama* mtDNA control region dataset. Generated in BEAST utilising the strict-clock and exponential growth demographic model. Bars correspond to 95% highest posterior density (HPD) of divergence estimates. Posterior probability support values are given at respective nodes. The ‘//’ symbol denotes a shortening of branch length for the outgroup. The values given at the root node are the divergence time estimate (Ka) followed by the 95% HPD in parentheses. The oldest haplotypes (from the Neolithic) are indicated with a red dot. Red and yellow colours show well-supported lineages.

Estimated node dating for the CR phylogeny based on analysis in BEAST is presented in Figs. 3 and 4. For both the ancient-only (Fig. 3) and combined datasets (Fig. 4), the model with the highest support was the strict clock with exponential growth (EG; Tables S4 and S5). Based on mean date estimates, there is a split at the Late Pleistocene or early-Holocene between Anatolia and Italy/Iberia for each tree (Figs. 3 and 4). Diversification into northern regions could have started as early as around ten thousand or as recent as 2000 years ago, based on confidence intervals. These phylogenies indicate that Roman Europe including England have diversified within the Italian/Iberian lineage while northern and central European regions are diversifying from Anatolia (Turkey). The precise timing is not known from these analyses due to the broad confidence limits. Italy and Iberia diverge around the time of the Neolithic, but there is some paraphyly in this relationship (a group that includes the common ancestor, but not all of its descendants), possibly consistent with the anthropogenic transfer of lineages from one location to the other. For each of these trees it is possible that some of the topology reflects incomplete lineage sorting, though clusters are sensible (regional samples generally clustering together).

One sample from the Balkans (from Roman period Bulgaria) is basal to a lineage that includes modern Iberian samples, together with some Italian haplotypes that are paraphyletic to the main Italian lineage. When all samples are pooled together for an analysis using BEAST (Fig. 4), ancient and modern samples from a given region group together, and mostly diverge within the last 5000 years (though confidence limits on some nodes are broad). The Greek samples of modern Rhodes and ancient Chios cluster together and diverge around the Neolithic or before. Node dates are consistent across the phylogeny, but the confidence limits are broad, which necessarily leads to possible alternative interpretations. For example, the main Italian and Iberian lineages in the complete phylogeny (ancient and modern; Fig. 4) split at a time that could be consistent with Neolithic translocations, but also could be consistent with a split during the last glacial period. The same is true of the split between the Anatolian and Italian/Iberian lineages. This is the deepest node, which could be consistent with a split during the last glacial period, but the Neolithic period is also within the confidence limits, especially for the ancient-only phylogeny in Fig. 3.

Several samples dated back to the Neolithic/Bronze Age period, representing haplotypes H31, H45 and H46 (see Table S1, Figs. 3 and 4). The clustering together of ancient Chios (late Neolithic) and modern Rhodes suggests continuity in that region (Greek islands) over that timeframe (Fig. 4). The division between the modern and ancient samples in that lineage is consistent with the proposed ancient vintage of the Chios samples. A sample from the late Neolithic in Turkey (H31) clusters with samples from Turkey in Roman or Byzantine periods (Fig. 3), and that lineage clusters with samples from modern Turkey (Fig. 4), again suggesting a long period of continuity. The broader lineage, including samples from Turkey, also includes samples from medieval to modern times in Britain, northern Europe and elsewhere in the world. These samples are predominantly in populations known to have experienced translocations in the past. The phylogenies suggest the possibility of two main translocation events out of Turkey, one earlier than the other, but both likely to be quite early (within the Neolithic; Figs. 3 and 4).

Ancient Italian samples all group together in a lineage with two clusters, one representing samples dating from the 2nd to the seventeenth centuries, and the other with one sample from the seventeenth century and two from Roman France (Fig. 3). Samples from modern Italy are also within this lineage, but two modern haplotypes cluster within a lineage dominated by Iberian samples. The broader lineage (one of two main lineages that could have split between the early Neolithic and the middle of the last glacial period; Figs. 3 and 4) includes other northern Roman sites, including Roman Britain, together with ancient and modern samples from Iberia. The Iberian samples all cluster together and include samples dating from Roman to modern times, again suggesting continuity over that timeframe. The modern Italian samples within this lineage seem credible recent translocations from Iberia into Italy. Two samples from Roman period Bulgaria share the haplotype H26, and cluster most closely with the Iberian lineage.

**Figure 5:**
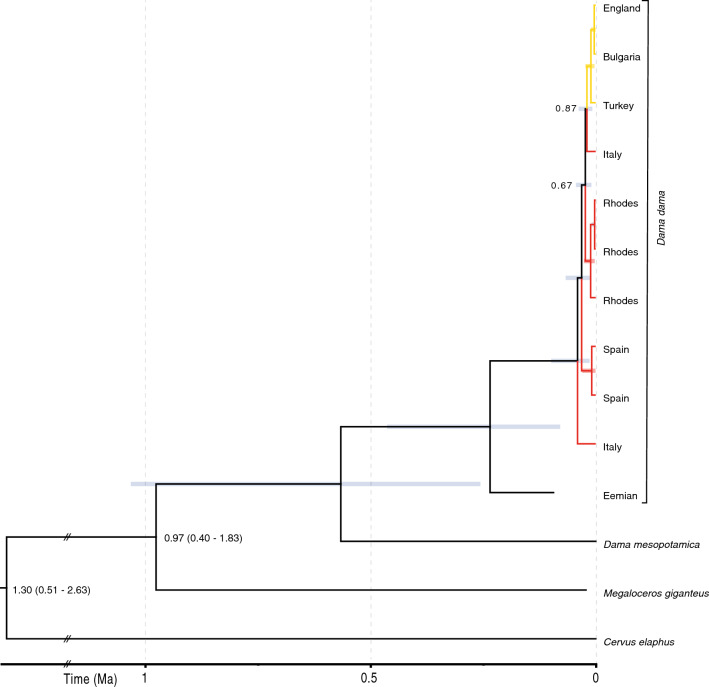
Time-calibrated tree using the mitogenome *D. dama* mtDNA dataset, including the haplotype from the Eemian. Generated in BEAST utilising the uncorrelated lognormal relaxed clock and the logistic growth demographic model. Bars at nodes correspond to 95% highest posterior density (HPD) of divergence estimates (Ma). Posterior probability support values are given at respective nodes. Only posterior probabilities less than one are shown. The ‘//’ symbol denotes a shortening of branch length for the outgroup. The values given at the two deepest nodes, representing divergences with *Cervus elephus* (root) and *Megaloceros giganteus* outgroups, are the divergence time estimates (Ma) followed by the 95% HPD in parentheses. Red and yellow colours show well-supported lineages.

Only one of the two Eemian samples provided enough sequence data to be useful (Table S5). This sample was compared against 14 modern sample mitogenomes (including the reference sequence), all of which sequenced with full coverage. For the Eemian sample, we used a uracil DNA glycosylase to convert post-mortem C/T transitions (see methods). However, we also employed a conservative approach whereby C/T transitions were only retained if shared with another sequence. If unique to the Eemian sample, they were converted to an ‘N’. The topology of the resulting phylogenies was the same as the trees retaining the identified base pairs (data not shown). The phylogenies based on shared sequence data between the modern mitogenomes and the fragmented Eemian sequence (retaining sequence with at least 4X coverage) included a shared total of 5618 bp (including segments from across the mitogenome, but there was insufficient coverage to include the control region; Table S5b). The Bayesian tree is shown in Figure S2, and the BEAST tree in Fig. 5. The Eemian sample is basal to both lineages, but average genetic distance (see matrix in Table S5a) is closer to the lineage including the Italian samples (mean = 0.0211, range = 0.02038–0.021943; N = 7; red lineage in Figs. S2 and S5) than to the other lineage which includes a Turkish sample (mean = 0.02204, range = 0.021955–0.022152; N = 7; yellow in Figs. S2 and S5). Note that the sample labelled ‘Bulgaria’ in Fig. 4 had the same haplotype as two other samples (one from England and the reference: NCBI accession = JN632629.1) and the sample marked ‘Turkey’ was the same haplotype as two other samples from modern Turkey. The Bulgarian sample in Figs. S2 and S5 is from modern Bulgaria, not the Roman period as in H-26 in Fig. 3, and likely represents a recent introduction. A non-parametric Mann–Whitney U-test (applied because sample sizes are too small to confirm normality) showed a significant difference comparing genetic distance of the Eemian sample to the red and yellow lineages (Z = 3.07, *p* = 0.002). The analysis in BEAST (Fig. 5) set the terminal node date for the Eemian sample at 125 ka, and the node separating the Rhodian lineage to 8 ka. The consequent node date for the division between the Turkish and Italian lineages was 19.4 ka; 95% HPD (7.7, 37.7; see Fig. 5). The date of the node separating the Eemian sample from the rest of the lineage [233.8 ka; 95% HPD (78.7, 461.9)] was consistent with the age of the sample (possibly including some divergence between the northern population and the founder population in the south).

## Discussion

The data show lineage sharing among haplotypes from a range of geographic regions in Britain, central/northern Europe, Barbuda and Canada, and those lineages date to relatively recent periods (Figs. 3 and 4), consistent with what we know about the extensive translocations there. In contrast, southern populations (i.e. Turkish, Iberian, and Italian samples) all form clusters dominated by (or exclusive to) samples from the same region, which are stable through time and include a temporal sampling range from the late Neolithic to modern times (Turkey), and Roman to modern times (Iberia and Italy). The apparent continuity of lineages over time in these southern regions (supported in all of our phylogenetic reconstructions; see Figs. 2, 3 and 4) suggests populations that have not been substantially changed since their initial founding. Based on the genetic data alone, these southern regions could be interpreted as representing glacial refugia. However, the extensive zooarchaeological data presented by^2^ demonstrate long periods of absence of fallow deer in Iberia and Italy. At the same time, the data suggest that unlike the northern populations, populations in Iberia and Italy were not subject to extensive translocations and local extinctions. This suggests periods of relative stability and limited connectivity.

Node dating in our phylogenetic reconstructions showed broad confidence limits, and these estimates are dependent on tree priors^44^. However, key division points were within a time range that are consistent with our hypotheses. Given the sample set available, we find two well supported lineages (depicted in the figures as yellow and red) that most likely diverged in the Late Pleistocene or Early Holocene (the timeframe representing most of the highest posterior probability distribution (HPD); see Figs. 3 and 4). This is consistent with a signal from two main refugial populations that formed when populations contracted during the last glacial period. One of these lineages is dominated by samples from Anatolia and translocated populations further north. The other also includes translocated northern populations together with lineages from Italy and Iberia. The division between lineages mostly represented by Italy and those mostly represented by Iberia is also quite old, with the HPD ranging from the Neolithic to the Late Pleistocene (consistent for both the CR phylogeny in Fig. 4 and the mitogenomic phylogeny in Fig. 5).

Populations in proposed refugial regions show lineage consistency over time, and phylogenetic division during the last glacial period. At the same time, there are some indications of possible signals from translocations in these regions, such as a possible translocation from the Balkans to Iberia ~ 10 ka, and a more recent translocation from Iberia to Italy. The dominant pattern, however, is stable lineage structure over time in locations (e.g. Iberia and Italy) known to reflect refugia for many European mammal species (see review in^7^) but the confidence limits also overlap with periods when humans were well known to have been translocating fallow deer. Baker et al.^2^ extensive zooarchaeological data would appear to rule out long-term occupancy of fallow deer in Iberia and Italy, and on this basis the idea of two refugial populations in the east (one in the Balkans and one in Anatolia) seems best supported (though this interpretation is open to revision based on further genetic or zooarchaeological data). This scenario is consistent with a phylogeographic break across the Bosphorus strait which has been shown to promote diversification in a range of other species^45^ and early translocations. Our inclusion of data from a sample that pre-dates the formation of refugia during the last glacial period allows some inference about the formation of refugia. The Eemian sample, while also reflecting divergence over time (comparing the ancient with modern samples), was most closely related to the lineage that included samples from Iberia and Italy (red in the figures), so this possibly reflects the descendants from a lineage originally formed there or in the Balkans. It suggests that a Balkan refuge would be at least as old as the one in Anatolia. Note that the posterior distribution for the date of the node separating the Eemian sample includes the age difference (~ 130,000 years), and therefore we cannot conclude any taxonomic distinction between the Eemain sample and modern *D. d. dama* populations.

## Conclusions

In an increasingly manipulated world, it is important to consider the conservation and management implications of the interaction between anthropogenic and natural processes. Here, time series data has allowed us to see both the effects of extensive relocation over relatively recent times, consistent with the documented movement of this species among parks and reserves^7^, and the persistent signal of ancestral populations, displaced from a range that covered most of Europe as recently as the Eemian interglacial, and remained in southern refugial populations (as seen for a few other species as well, see^46^).

### Supplementary Information

Supplementary Information.

## Data Availability

All DNA sequences are available on Genbank under accession numbers KY564399–KY564432; OR220344-OR220389, OR531442, OR531443, OR232305-OR232317 and in the supplement.
