# Supplementary material for: Ancient and modern DNA track temporal and spatial population dynamics in the European fallow deer since the Eemian interglacial
Source: Sci Rep. 2024 Feb 12;14:3015. doi: 10.1038/s41598-023-48112-6 (PMC10861457; doi:10.1038/s41598-023-48112-6)
Supplement: Supplementary file 1 — Supplementary Information. [file 41598_2023_48112_MOESM1_ESM.docx]

**Supplementary:**

**Figure S1:** Mr Bayes tree with ancient and modern haplotypes combined.


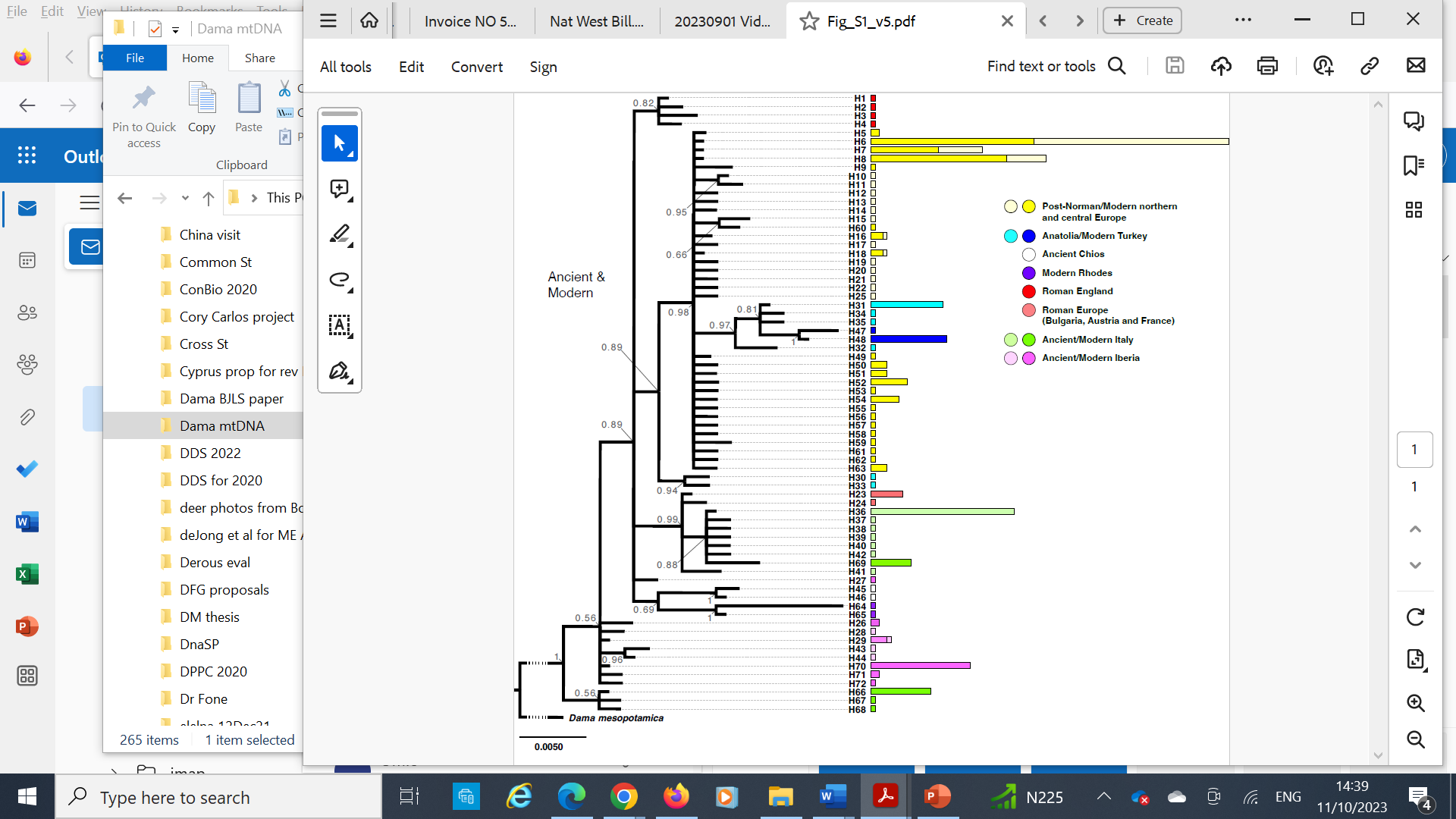


**Figure S2:** Bayesian phylogeny based on the partitioned mt-genome dataset and including the sample from the Eemian.


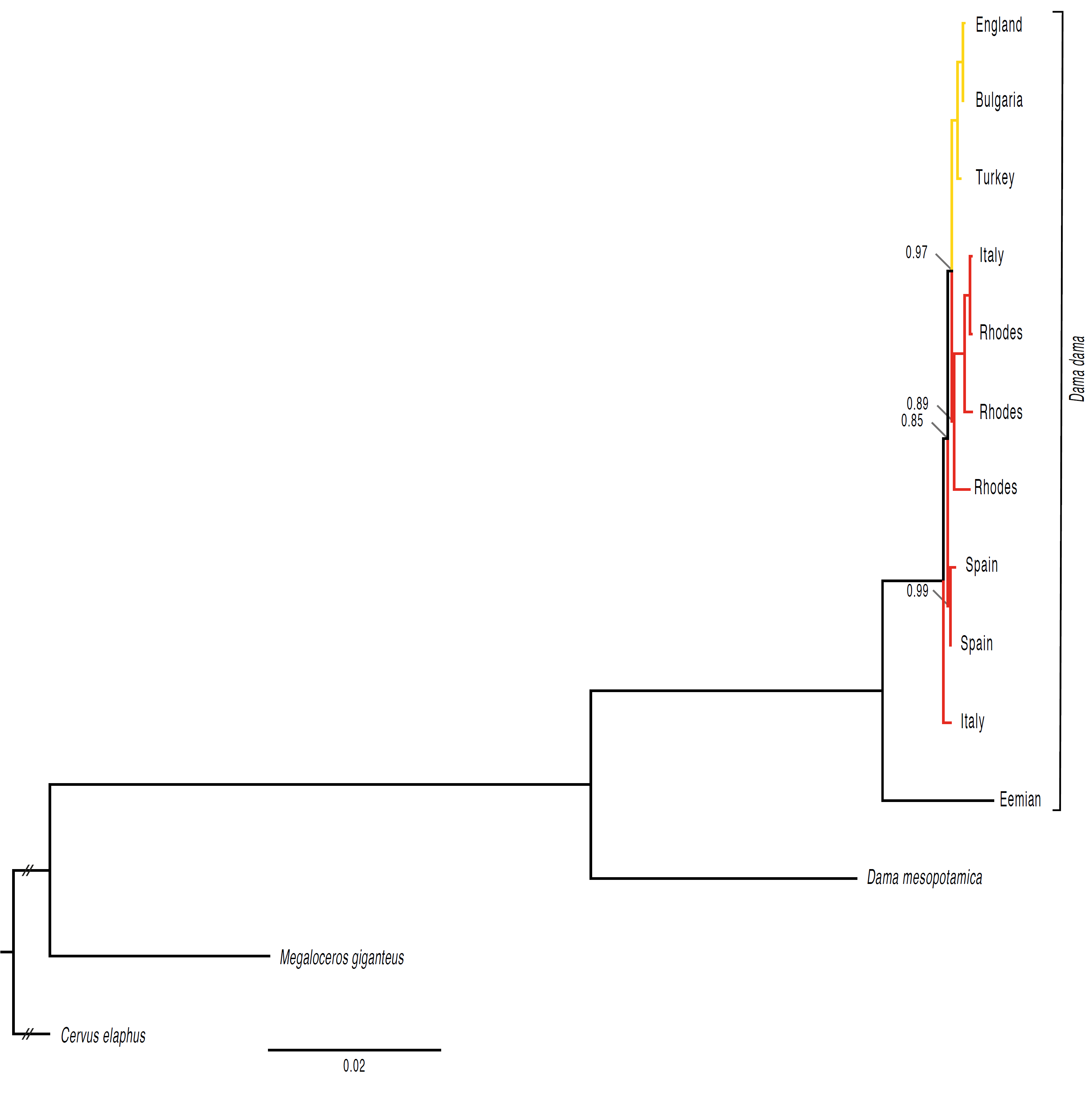


**Table S1:** List of haplotypes for ancient and modern combined and ancient and modern individually. The haplotype numbers correspond to the numbers shown in the median joining networks and trees.

| **Ancient and modern combined** | | **Ancient only** | | **Modern only** | | **Age range** | **Location** |
| --- | --- | --- | --- | --- | --- | --- | --- |
| **Hap no** | **Total indivs** | **Hap no** | **Total indivs** | **Hap no** | **Total indivs** |  |  |
| H1 | 20 | H1 | 20 |  |  | Roman | **Roman UK:** Monkton |
| H2 | 1 | H2 | 1 |  |  | Roman | **Roman UK:** Monkton |
| H3 | 1 | H3 | 1 |  |  | Roman | **Roman UK:** Fishbourne |
| H4 | 1 | H4 | 1 |  |  | Roman | **Roman UK:** Fishbourne |
| H5 | 4 | H5 | 2 | h14 | 2 | Early Medieval to Modern | **Post Norman England:** (2) Goltho Modern England: (2) Belton House |
| H6 | 90 | H6 | 56 | h3 | 34 | Early Medieval to Modern | **Post Norman England: (49)** Goltho, Sparsholt Villa, Dudley Castle, Hungate, Faccombe Netherton Cheddar Palaces, Carisbrooke, Hyde Abbey **Post Norman Ireland (7)**: Green castle, Newton Stewart and Trim Castle Ireland **Modern England:** (10) Belton House, Holkham, Cheshire, Lincolnshire, Attingham **Modern Ireland:** (11) Dublin, Monoghan, Wicklow, West Meath, Mayo **Modern EU: (12)** Slovenia (1), Sweden (7) Portugal (4) **Modern International:** (1) Barbuda |
| H7 | 28 | H7 | 11 | h15 | 17 | Early Medieval to Modern | **Post Norman England (11):** Goltho, Dudley Castle, Hungate, Binchester, Belton House **Modern England (8)**: Hatfield, Cambridgeshire, Wytham, Lincolnshire, Suffolk **Modern Ireland:** (1) **Modern EU (5):** Slovenia (2) and Hungary (2) **Modern International (3):** Canada (3) |
| H8 | 45 | H8 | 10 | h5 | 35 | Early Medieval to Modern | **Post Norman England: (4)** Goltho, Hungate , Faccombe Netherton **Post Norman Irleand:** (6) Green Castle, Trim Castle, Kilkenny **Modern England (9):**  Lincolnshire (2), Oxfordshire (5),Holkham (2) **Modern EU (25):** Hungary (9), Germany (1), Bulgaria (11), Piedmont (4) |
| H9 | 1 | H9 | 1 |  |  | Early Medieval | **Post Norman England:** Dudley Castle |
| H10 | 2 | H10 | 2 |  |  | Early Medieval | **Post Norman England:** Dudley Castle |
| H11 | 1 | H11 | 1 |  |  | Early Medieval | **Post Norman England:** Dudley Castle |
| H12 | 1 | H12 | 1 |  |  | Early Medieval | **Post Norman England**: Hungate |
| H13 | 1 | H13 | 1 |  |  | Early Medieval | **Post Norman England**: Hungate |
| H14 | 1 | H14 | 1 |  |  | Early Medieval | **Post Norman England**: Hungate |
| H15 | 1 | H15 | 1 |  |  | Early Medieval | **Post Norman England**: Hungate |
| H16 | 4 | H16 | 1 | h4 | 3 | Early Medieval to Modern | **Post Norman England:** (1) Faccombe Netherton  **Modern Ireland:** (3) Galway and Kilverstone |
| H17 | 1 | H17 | 1 |  |  | Early Medieval | **Post Norman UK:** (1) Faccombe Netherton |
| H18 | 4 | H18 | 3 | h19 | 1 | Early Medieval to Modern | **Post Norman UK:** (3) Eynsham Abbey, Green Castle **Modern EU:** (1) Germany |
| H19 | 1 | H19 | 1 |  |  | Early Medieval | **Post Norman England:** Lewes Castle |
| H20 | 1 | H20 | 1 |  |  | Early Medieval | **Post Norman England:** Carisbrooke Castle |
| H21 | 1 | H21 | 1 |  |  | Early Medieval | **Post Norman England:** Arcadia Building |
| H22 | 3 | H22 | 3 |  |  | Early Medieval | **Post Norman England:** Trim Castle |
| H23 | 8 | H23 | 8 |  |  | Roman to 14th Century | **Roman France** (7) A**ncient Italy:** (1) Sassari |
| H24 | 1 | H24 | 1 |  |  | Roman | **Roman France** (1) |
| H25 | 1 | H25 | 1 |  |  | 16th-17th Century | **Medieval Belgium:** Boussu |
| H26 | 2 | H26 | 2 |  |  | Roman | **Roman Bulgaria** (2) |
| H27 | 1 | H27 | 1 |  |  | Roman | **Roman Austria:** Carnutum Mulhacker |
| H28 | 1 | H28 | 1 |  |  | Medieval | **Medieval Portugal:** Castelo de Pumela |
| H29 | 1 | H29 | 1 | h31 | 4 | Medieval to Modern | **Medieval Portugal** (1) **Modern Portugal** (4) |
| H30 | 1 | H30 | 1 |  |  | 12^th^ to 13^th^ Century | **Ancient Turkey:** Kinet Höyük |
| H31 | 18 | H31 | 18 |  |  | Early Neolithic to Byzantine | **Ancient Turkey:** Bademağaci (2), Sagalassos (16) |
| H32 | 1 | H32 | 1 |  |  | Early Byzantine | **Ancient Turkey:** Sagalassos |
| H33 | 1 | H33 | 1 |  |  | Classical/Hellenistic | **Ancient Turkey:** Sagalassos |
| H34 | 1 | H34 | 1 |  |  | Roman/Byzantine | **Ancient Turkey:** Sagalassos |
| H35 | 1 | H35 | 1 |  |  | Roman/Byzantine | **Ancient Turkey:** Sagalassos |
| H36 | 11 | H36 | 11 |  |  | 2^nd^ to 17^th^ Century | **Ancient Italy** (36): Sassari and Sicily |
| H37 | 1 | H37 | 1 |  |  | 14^th^ Century | **Ancient Italy** (1): Sassari |
| H38 | 1 | H38 | 1 |  |  | 14^th^ Century | **Ancient Italy** (1): Sassari |
| H39 | 1 | H39 | 1 |  |  | 17^th^ Century | **Ancient Italy** (1): Sassari |
| H40 | 1 | H40 | 1 |  |  | 4^th^ to 5^th^ Century | **Ancient Italy** (1): Sassari |
| H41 | 1 | H41 | 1 |  |  | 13^th^ Century | **Ancient Italy** (1): Sicily |
| H42 | 1 | H42 | 1 |  |  | 13^th^ Century | **Ancient Italy** (1): Sicily |
| H43 | 1 | H43 | 1 |  |  | Roman | **Ancient Spain** (1): Torre de Palma |
| H44 | 1 | H44 | 1 |  |  | Roman | **Ancient Spain** (1): Torre de Palma |
| H45 | 1 | H45 | 1 |  |  | Late Neolithic/Early Bronze | **Ancient Chios** (1) |
| H46 | 1 | H46 | 1 |  |  | Late Neolithic/Early Bronze | **Ancient Chios** (1) |
| H47 | 1 |  |  | h1 | 1 | Modern | **Modern Turkey** (1) |
| H48 | 19 |  |  | h2 | 19 | Modern | **Modern Turkey** (19) |
| H49 | 1 |  |  | h6 | 1 | Modern | **Modern Ireland:** Roscommon |
| H50 | 4 |  |  | h7 | 4 | Modern | **Modern England** (1): Houghton **Modern EU** (3): Hungary and Germany |
| H51 | 4 |  |  | h8 | 4 | Modern | **Modern England** (3): Suffolk, Attingham **Modern EU:** Germany |
| H52 | 9 |  |  | h9 | 9 | Modern | **Modern UK:** 9 Dartmoor, Ashridge |
| H53 | 4 |  |  | h10 | 4 | Modern | **Modern UK:** Chilterns |
| H54 | 7 |  |  | h11 | 7 | Modern | **Modern England** (5) Epping Forest, Cambridge, Whipsnade Zoo **Modern EU:** (2): Germany |
| H55 | 1 |  |  | h12 | 1 | Modern | **Modern England:** Linken Holt |
| H56 | 2 |  |  | h13 | 2 | Modern | **Modern England:** Houghton |
| H57 | 2 |  |  | h16 | 2 | Modern | **Modern EU:** Germany |
| H58 | 2 |  |  | h17 | 2 | Modern | **Modern EU:** Germany |
| H59 | 1 |  |  | h18 | 1 | Modern | **Modern EU:** Germany |
| H60 | 1 |  |  | h20 | 1 | Modern | **Modern England:** Lincolnshire |
| H61 | 1 |  |  | h21 | 1 | Modern | **Modern England:** Horringer |
| H62 | 1 |  |  | h22 | 1 | Modern | **Modern EU:** Germany |
| H63 | 4 |  |  | h23 | 4 | Modern | **Modern International:** (4) Barbuda |
| H64 | 1 |  |  | h24 | 1 | Modern | **Modern Rhodes** |
| H65 | 3 |  |  | h25 | 3 | Modern | **Modern Rhodes** |
| H66 | 15 |  |  | h26 | 15 | Modern | **Modern Italy and Germany:** Italy (14) (Grossetto, Siena, Piedmont) and Germany (1) |
| H67 | 1 |  |  | h27 | 1 | Modern | **Modern Italy:** Italy(1) Siena |
| H68 | 1 |  |  | h28 | 1 | Modern | **Modern Italy:** Italy(1) Siena |
| H69 | 10 |  |  | h29 | 10 | Modern | **Modern Italy** (10): Siena, San Rossore, Piedmont, Palazetto, Ontanelli |
| H70 | 25 |  |  | h30 | 25 | Modern | **Modern Portugal and Spain:** Portugal (9), Spain (16) North Madrid, Jaen, Asturias |
| H71 | 2 |  |  | h32 | 2 | Modern | **Modern Spain** (2): Huelva |
| H72 | 1 |  |  | h33 | 1 | Modern | **Modern Spain** (1): Toledo |

**Table S2**: Forward and reverse primers used to amplify a partial region of the mtDNA d-loop.

| **Primer set** | **Primer** | **Primer sequence 3'-5'** | **PCR product length** | **Annealing Temperature** |
| --- | --- | --- | --- | --- |
| Fallow 1f and 1r | Fallow 1f | TTTAAACTATTCCCTGACGCTTA | 253 | 501 |
|  | Fallow 1r | AAGCATGGGGTATATGTAATGT |  |  |
| Dama 4f and 5r | Dama 4f | ACATTACATTATATACCCCATGCTT | 364 | 59 |
|  | Dama 5r | CACACAGTTATGTGTGAGCA |  |  |
| **Alternative primers for 4f and 5r region** | | |  |  |
| Ps6f and 6r | PS6F | TCTGGTTYTTTCTTCAGGGCCA | 231 | 572 |
|  | PS6R | GGGATGCTCAAGATGCAGTT |  |  |
| RD3F and 3r | RD3F | GATCACGAGCTTGATTACC | 149 | 572 |
|  | RD3R | TTAGGTGAGATGGCCCTGAA |  |  |
| 2f new and 2r new | 2f new | CAGCAAAACATGTGATACAAYCC | 239 | 572 |
|  | 2r new | GCGGCATGGTAATTAAGCTC |  |  |

**Table S3a**: Log marginal likelihoods for three independent runs under different models and datasets, estimated in BEAST using stepping-stone sampling (Xie *et al*. 2011; Baele *et al.* 2013). R1 = 100 power posteriors over 1,000,000 iterations R2 = 100 power posteriors over 2,000,000 iterations. The model with the highest log-marginal likelihoods for combined runs is highlighted in yellow; lower values are in orange. CS = constant size, EG = Exponential Growth, ExG = Expansion Growth, LG = Logistic Growth, UCLD = relaxed clock, Strict = strict clock, Fixed = fixed clock, RL = random local clock.

| **Control Region, Ancient-only dataset** | | | | | | | | | |
| --- | --- | --- | --- | --- | --- | --- | --- | --- | --- |
|  |  | **log marginal Likelihood (R1)** | | | | **log marginal Likelihood (R2)** | | | |
| **Clock** | **Pop. Model** | **log mle run1** | **log mle run2** | **log mle run3** | **combined** | **log mle run1** | **log mle run2** | **log mle run3** | **combined** |
| Strict | CS | -1444.80 | -1445.34 | -1444.99 | -1445.03 | -1445.25 | -1445.25 | -1445.58 | -1445.35 |
| Strict | EG | -1442.34 | -1442.21 | -1442.27 | -1442.26 | -1442.46 | -1442.19 | -1442.46 | -1442.36 |
| UCLD | CS | -1468.42 | -1468.32 | -1468.86 | -1468.38 | -1468.37 | -1468.63 | -1468.66 | -1468.44 |
| UCLD | EG | -1462.43 | -1463.28 | -1464.49 | -1463.21 | -1463.38 | -1463.44 | -1463.64 | -1463.42 |
|  |  |  |  |  |  |  |  |  |  |
| **Control Region, Ancient+Modern dataset** | | | | | | | | | |
|  |  | **log marginal Likelihood (R1)** | | | | **log marginal Likelihood (R2)** | | | |
| **Clock** | **Pop_Model** | **log mle run1** | **log mle run2** | **log mle run3** | **combined** | **log mle run1** | **log mle run2** | **log mle run3** | **combined** |
| Strict | CS | -1838.92 | -1839.20 | -1838.89 | -1838.93 | -1838.74 | -1838.91 | -1839.05 | -1838.88 |
| Strict | EG | -1837.26 | -1837.57 | -1837.64 | -1837.42 | -1836.92 | -1837.45 | -1837.08 | -1837.12 |
| UCLD | CS | -1851.46 | -1852.52 | -1853.27 | -1852.13 | -1852.40 | -1853.88 | -1853.88 | -1853.20 |
| UCLD | EG | -1848.57 | -1848.70 | -1849.92 | -1848.81 | -1849.99 | -1848.75 | -1849.01 | -1849.01 |
|  |  |  |  |  |  |  |  |  |  |
| **Mitogenome dataset** | |  |  |  |  |  |  |  |  |
|  |  | **log marginal Likelihood (R1)** | | | | **log marginal Likelihood (R2)** | | | |
| **Clock** | **Pop_Model** | **log mle run1** | **log mle run2** | **log mle run3** | **combined** | **log mle run1** | **log mle run2** | **log mle run3** | **combined** |
| Fixed | CS | -13051.65 | -13051.54 | -13051.59 | -13051.59 | -13051.55 | -13051.50 | -13051.51 | -13051.52 |
| Fixed | EG | -13050.26 | -13050.16 | -13050.26 | -13050.22 | -13050.06 | -13050.09 | -13050.18 | -13050.11 |
| Strict | CS | -13114.87 | -13114.70 | -13114.70 | -13114.75 | -13114.59 | -13114.81 | -13114.81 | -13114.74 |
| Strict | EG | -13113.89 | -13113.83 | -13113.79 | -13113.84 | -13113.78 | -13113.70 | -13113.86 | -13113.78 |
| UCLD | CS | -13056.21 | -13056.41 | -13056.35 | -13056.32 | -13056.29 | -13056.95 | -13056.40 | -13056.53 |
| UCLD | EG | -13057.55 | -13057.36 | -13057.69 | -13057.52 | -13057.43 | -13057.65 | -13057.78 | -13057.61 |
| RL | CS | -13058.30 | -13058.32 | -13057.75 | -13058.11 | -13058.19 | -13058.08 | -13058.08 | -13058.11 |
| RL | EG | -13057.58 | -13057.39 | -13057.04 | -13057.32 | -13056.85 | -13056.90 | -13056.92 | -13056.89 |
| Fixed | ExG | -13046.90 | -13047.11 | -13046.93 | -13046.98 | -13046.97 | -13047.12 | -13047.07 | -13047.05 |
| Fixed | LG | -13046.79 | -13046.63 | -13046.65 | -13046.68 | -13046.81 | -13046.65 | -13046.66 | -13046.71 |
| Fixed | EG* | -13059.59 | -13059.62 | -13059.70 | -13059.63 | -13059.59 | -13059.75 | -13059.75 | -13059.69 |
| Fixed | EG** | -13050.17 | -13050.15 | -13050.14 | -13050.15 | -13050.21 | -13050.09 | -13050.16 | -13050.15 |
| UCLD | LG | -13045.42 | -13045.13 | -13045.47 | -13045.34 | -13045.48 | -13045.34 | -13045.34 | -13045.39 |
| UCLD | ExG | -13045.74 | -13045.71 | -13045.70 | -13045.72 | -13045.59 | -13045.52 | -13045.59 | -13045.57 |

**Table S3b**: Pairwise log bayes factors for all BEAST models compared within each dataset, calculated from log marginal likelihoods estimated from stepping-stone sampling (Xie *et al*. 2011; Baele *et al.* 2013). Where the values are positive, the model in the row is better supported. Models are as listed in Table S5.

| **Control Region, Ancient-only** | | | | |  |  |  |  |  |  |  |  |  |  |
| --- | --- | --- | --- | --- | --- | --- | --- | --- | --- | --- | --- | --- | --- | --- |
| **log Bayes Factors** | | |  |  |  |  |  |  |  |  |  |  |  |  |
|  | **1** | **2** | **3** | **4** |  |  |  |  |  |  |  |  |  |  |
| **1** |  | -2.77 | 23.36 | 18.19 |  |  |  |  |  |  |  |  |  |  |
| **2** | 2.77 |  | 26.13 | 20.96 |  |  |  |  |  |  |  |  |  |  |
| **3** | -23.36 | -26.13 |  | -5.17 |  |  |  |  |  |  |  |  |  |  |
| **4** | -18.19 | -20.96 | 5.17 |  |  |  |  |  |  |  |  |  |  |  |
|  |  |  |  |  |  |  |  |  |  |  |  |  |  |  |
| **Control Region, Ancient+Modern** | | | | |  |  |  |  |  |  |  |  |  |  |
| **log Bayes Factors** | | |  |  |  |  |  |  |  |  |  |  |  |  |
|  | **1** | **2** | **3** | **4** |  |  |  |  |  |  |  |  |  |  |
| **1** |  | -1.51 | 13.20 | 9.89 |  |  |  |  |  |  |  |  |  |  |
| **2** | 1.51 |  | 14.71 | 11.40 |  |  |  |  |  |  |  |  |  |  |
| **3** | -13.20 | -14.71 |  | -3.32 |  |  |  |  |  |  |  |  |  |  |
| **4** | -9.89 | -11.40 | 3.32 |  |  |  |  |  |  |  |  |  |  |  |
|  |  |  |  |  |  |  |  |  |  |  |  |  |  |  |
| **Mitogenome** | | |  |  |  |  |  |  |  |  |  |  |  |  |
| **log Bayes Factors** | | |  |  |  |  |  |  |  |  |  |  |  |  |
|  | **1** | **2** | **3** | **4** | **5** | **6** | **7** | **8** | **9** | **10** | **11** | **12** | **13** | **14** |
| **1** |  | -1.37 | 63.16 | 62.25 | 4.73 | 5.93 | 6.52 | 5.73 | -4.61 | -4.90 | 8.04 | -1.44 | -6.25 | -5.87 |
| **2** | 1.37 |  | 64.53 | 63.61 | 6.09 | 7.29 | 7.89 | 7.10 | -3.25 | -3.54 | 9.41 | -0.07 | -4.89 | -4.51 |
| **3** | -63.16 | -64.53 |  | -0.92 | -58.44 | -57.24 | -56.64 | -57.43 | -67.78 | -68.07 | -55.12 | -64.60 | -69.42 | -69.04 |
| **4** | -62.25 | -63.61 | 0.92 |  | -57.52 | -56.32 | -55.73 | -56.51 | -66.86 | -67.15 | -54.21 | -63.68 | -68.50 | -68.12 |
| **5** | -4.73 | -6.09 | 58.44 | 57.52 |  | 1.20 | 1.79 | 1.01 | -9.34 | -9.63 | 3.31 | -6.16 | -10.98 | -10.60 |
| **6** | -5.93 | -7.29 | 57.24 | 56.32 | -1.20 |  | 0.59 | -0.19 | -10.54 | -10.83 | 2.11 | -7.36 | -12.18 | -11.80 |
| **7** | -6.52 | -7.89 | 56.64 | 55.73 | -1.79 | -0.59 |  | -0.79 | -11.13 | -11.43 | 1.52 | -7.96 | -12.77 | -12.39 |
| **8** | -5.73 | -7.10 | 57.43 | 56.51 | -1.01 | 0.19 | 0.79 |  | -10.35 | -10.64 | 2.31 | -7.17 | -11.99 | -11.61 |
| **9** | 4.61 | 3.25 | 67.78 | 66.86 | 9.34 | 10.54 | 11.13 | 10.35 |  | -0.29 | 12.65 | 3.18 | -1.64 | -1.26 |
| **10** | 4.90 | 3.54 | 68.07 | 67.15 | 9.63 | 10.83 | 11.43 | 10.64 | 0.29 |  | 12.95 | 3.47 | -1.35 | -0.97 |
| **11** | -8.04 | -9.41 | 55.12 | 54.21 | -3.31 | -2.11 | -1.52 | -2.31 | -12.65 | -12.95 |  | -9.48 | -14.29 | -13.92 |
| **12** | 1.44 | 0.07 | 64.60 | 63.68 | 6.16 | 7.36 | 7.96 | 7.17 | -3.18 | -3.47 | 9.48 |  | -4.82 | -4.44 |
| **13** | 6.25 | 4.89 | 69.42 | 68.50 | 10.98 | 12.18 | 12.77 | 11.99 | 1.64 | 1.35 | 14.29 | 4.82 |  | 0.38 |
| **14** | 5.87 | 4.51 | 69.04 | 68.12 | 10.60 | 11.80 | 12.39 | 11.61 | 1.26 | 0.97 | 13.92 | 4.44 | -0.38 |  |

**Table S4:** Details of the different models tested in BEAST for both control region datasets, including the priors used across all models (two calibrated nodes (TMRCA 1 & 2) and the haplotype ages) Strict = strict clock; UCLD = lognormal relaxed clock (uncorrelated); CS = constant size; EG = exponential growth TMRCA = time to most recent common ancestor Times in years before present.

|  | **Model 1** | **Model 2** | | **Model 3** | **Model 4** |  |  |
| --- | --- | --- | --- | --- | --- | --- | --- |
| **molecular clock** | Strict | Strict | | UCLD | UCLD |  |  |
| **demographic model** | CS | EG | | CS | EG |  |  |
| **PRIORS ACROSS ALL MODELS** | | | | | | | |
| **TMRCA1**  divergence of *D mesopotamica* and *D dama* | distribution: | | normal | mean | 700,000 | stdev | 50,000 |
| **TMRCA2**  divergence of Roman England *D dama* from Roman Europe | distribution: | | Lognormal | Mean  (real time) | 2000 | stdev  (real time) | 200 |
| age(Hap_1) | distribution: | | normal | mean = | 1907 | stdev = | 200 |
| age(Hap_2) | distribution: | | normal | mean = | 1865 | stdev = | 200 |
| age(Hap_3) | distribution: | | normal | mean = | 1865 | stdev = | 200 |
| age(Hap_4) | distribution: | | normal | mean = | 1865 | stdev = | 200 |
| age(Hap_5) | distribution: | | normal | mean = | 1003 | stdev = | 200 |
| age(Hap_6) | distribution: | | normal | mean = | 1003 | stdev = | 200 |
| age(Hap_7) | distribution: | | normal | mean = | 1003 | stdev = | 200 |
| age(Hap_8) | distribution: | | normal | mean = | 1003 | stdev = | 200 |
| age(Hap_9) | distribution: | | normal | mean = | 930 | stdev = | 50 |
| age(Hap_10) | distribution: | | normal | mean = | 550 | stdev = | 50 |
| age(Hap_11) | distribution: | | normal | mean = | 493 | stdev = | 50 |
| age(Hap_12) | distribution: | | normal | mean = | 665 | stdev = | 50 |
| age(Hap_13) | distribution: | | normal | mean = | 215 | stdev = | 50 |
| age(Hap_14) | distribution: | | normal | mean = | 465 | stdev = | 50 |
| age(Hap_15) | distribution: | | normal | mean = | 665 | stdev = | 50 |
| age(Hap_16) | distribution: | | normal | mean = | 697 | stdev = | 50 |
| age(Hap_17) | distribution: | | normal | mean = | 697 | stdev = | 50 |
| age(Hap_18) | distribution: | | normal | mean = | 640 | stdev = | 50 |
| age(Hap_19) | distribution: | | normal | mean = | 765 | stdev = | 50 |
| age(Hap_20) | distribution: | | normal | mean = | 770 | stdev = | 50 |
| age(Hap_21) | distribution: | | normal | mean = | 600 | stdev = | 50 |
| age(Hap_22) | distribution: | | normal | mean = | 615 | stdev = | 50 |
| age(Hap_23) | distribution: | | normal | mean = | 1865 | stdev = | 200 |
| age(Hap_24) | distribution: | | normal | mean = | 1865 | stdev = | 200 |
| age(Hap_25) | distribution: | | normal | mean = | 145 | stdev = | 50 |
| age(Hap_26) | distribution: | | normal | mean = | 1865 | stdev = | 200 |
| age(Hap_27) | distribution: | | normal | mean = | 1865 | stdev = | 200 |
| age(Hap_28) | distribution: | | normal | mean = | 415 | stdev = | 50 |
| age(Hap_29) | distribution: | | normal | mean = | 415 | stdev = | 50 |
| age(Hap_30) | distribution: | | normal | mean = | 600 | stdev = | 50 |
| age(Hap_31) | distribution: | | normal | mean = | 4015 | stdev = | 200 |
| age(Hap_32) | distribution: | | normal | mean = | 1460 | stdev = | 200 |
| age(Hap_33) | distribution: | | normal | mean = | 2815 | stdev = | 200 |
| age(Hap_34) | distribution: | | normal | mean = | 1460 | stdev = | 200 |
| age(Hap_35) | distribution: | | normal | mean = | 1460 | stdev = | 200 |
| age(Hap_36) | distribution: | | normal | mean = | 1915 | stdev = | 200 |
| age(Hap_37) | distribution: | | normal | mean = | 715 | stdev = | 50 |
| age(Hap_38) | distribution: | | normal | mean = | 715 | stdev = | 50 |
| age(Hap_39) | distribution: | | normal | mean = | 415 | stdev = | 50 |
| age(Hap_40) | distribution: | | normal | mean = | 1565 | stdev = | 200 |
| age(Hap_41) | distribution: | | normal | mean = | 1565 | stdev = | 200 |
| age(Hap_42) | distribution: | | normal | mean = | 1565 | stdev = | 200 |
| age(Hap_43) | distribution: | | normal | mean = | 1625 | stdev = | 200 |
| age(Hap_44) | distribution: | | normal | mean = | 1625 | stdev = | 200 |
| age(Hap_45) | distribution: | | normal | mean = | 4015 | stdev = | 200 |
| age(Hap_46) | distribution: | | normal | mean = | 4015 | stdev = | 200 |
| age(*D mesopotamica*) | distribution: | | normal | mean = | 50 | stdev = | 10 |

**Table S5**: a) Distance matrix based on GTR evolution model. Comparisons highlighted in yellow are against the yellow lineage (see Figure 4) and in red against the red lineage. b) Alignment of homologous regions of Eemian and other mitogenome sequences, supporting phylogeny shown in figure 5 and S2.

a)

b) Mitogenome fragment alignment with Eemian sequence data.

709_501Mod AACACGATAGCTAGGACCCAAACTGGGATTAGATACCCCACTATGCCT-AGCCCTAAACA

702_501Mod AACACGATAGCTAGGACCCAAACTGGGATTAGATACCCCACTATGCCT-AGCCCTAAACA

Eemian TACACGATAGCTAAGACCCAAACTGGGATTAGATACCCCACTATGCTTTAGCCCTAAACA

710_501Mod AACACGATAGCTAGGACCCAAACTGGGATTAGATACCCCACTATGCCT-AGCCCTAAACA

706_501Mod AACACGATAGCTAGGACCCAAACTGGGATTAGATACCCCACTATGCCT-AGCCCTAAACA

707_501Mod AACACGATAGCTAGGACCCAAACTGGGATTAGATACCCCACTATGCCT-AGCCCTAAACA

701_508Mod AACACGATAGCTAGGACCCAAACTGGGATTAGATACCCCACTATGCCT-AGCCCTAAACA

712_501Mod AACACGATAGCTAGGACCCAAACTGGGATTAGATACCCCACTATGCCT-AGCCCTAAACA

705_501Mod AACACGATAGCTAGGACCCAAACTGGGATTAGATACCCCACTATGCCT-AGCCCTAAACA

711_501Mod AACACGATAGCTAGGACCCAAACTGGGATTAGATACCCCACTATGCCT-AGCCCTAAACA

702_508Mod AACACGATAGCTAGGACCCAAACTGGGATTAGATACCCCACTATGCCT-AGCCCTAAACA

Dama_mesopotamica AACACGATAGCTAGGACCCAAACTGGGATTAGATACCCCACTATGCCT-AGCCTTAAACA

Irish_Elk AACACGATAGCTAGGACCCAAACTGGGATTAGATACCCCACTATGCCT-AGCCTTAAACA

red_deer AACACGATAGCTAGGACCCAAACTGGGATTAGATACCCCACTATGCCT-AGCCTTAAACA

************ ******************************** * **** ******

709_501Mod CAAATAGTTGTGTAAACAAAACTATTCGCCAGAGTACTACCGGCAATAGCTTAAAACTCA

702_501Mod CAAATAGTTGTGTAAACAAAACTATTCGCCAGAGTACTACCGGCAATAGCTTAAAACTCA

Eemian TAAATAGTTACC--AACAAAACTATTCGCCAGAGAACTACTAGCAACAGCTTAAAACTCA

710_501Mod CAAATAGTTGTGTAAACAAAACTATTCGCCAGAGTACTACCGGCAATAGCTTAAAACTCA

706_501Mod CAAATAGTTGTGTAAACAAAACTATTCGCCAGAGTACTACCGGCAATAGCTTAAAACTCA

707_501Mod CAAATAGTTGTGTAAACAAAACTATTCGCCAGAGTACTACCGGCAATAGCTTAAAACTCA

701_508Mod CAAATAGTTGTGTAAACAAAACTATTCGCCAGAGTACTACCGGCAATAGCTTAAAACTCA

712_501Mod CAAATAGTTGTGTAAACAAAACTATTCGCCAGAGTACTACCGGCAATAGCTTAAAACTCA

705_501Mod CAAATAGTTGTGTAAACAAAACTATTCGCCAGAGTACTACCGGCAATAGCTTAAAACTCA

711_501Mod CAAATAGTTGTGTAAACAAAACTATTCGCCAGAGTACTACCGGCAATAGCTTAAAACTCA

702_508Mod CAAATAGTTGTGTAAACAAAACTATTCGCCAGAGTACTACCAGCAATAGCTTAAAACTCA

Dama_mesopotamica CAAATAGTTATATAAACAAAACTATTCGCCAGAGTACTACCGGCAACAGCTTAAAACTCA

Irish_Elk CAAATAGTTATGTAAACAAAACTATTCGCCAGARTACTACCGGCAATAGCTTAAAACTCA

red_deer CAAATAGTTATGCAAACAAAACTATTCGCCAGAGTACTACCGGCAATAGCTTAAAACTCA

******** ******************* ***** **** *************

709_501Mod AAGGACTTGGCGGTGCTTTATACCCTTCTAGAGGAGCCTGTTCTATAATCGATAAACCCC

702_501Mod AAGGACTTGGCGGTGCTTTATACCCTTCTAGAGGAGCCTGTTCTATAATCGATAAACCCC

Eemian AAGGACTTGGCGGTGCTTCACACCCCTCTAGAGGAGCCTGTTCTATAATCGATAAACCCC

710_501Mod AAGGACTTGGCGGTGCTTTATACCCTTCTAGAGGAGCCTGTTCTATAATCGATAAACCCC

706_501Mod AAGGACTTGGCGGTGCTTTATACCCTTCTAGAGGAGCCTGTTCTATAATCGATAAACCCC

707_501Mod AAGGACTTGGCGGTGCTTTATACCCTTCTAGAGGAGCCTGTTCTATAATCGATAAACCCC

701_508Mod AAGGACTTGGCGGTGCTTTATACCCTTCTAGAGGAGCCTGTTCTATAATCGATAAACCCC

712_501Mod AAGGACTTGGCGGTGCTTTATACCCTTCTAGAGGAGCCTGTTCTATAATCGATAAACCCC

705_501Mod AAGGACTTGGCGGTGCTTTATACCCTTCTAGAGGAGCCTGTTCTATAATCGATAAACCCC

711_501Mod AAGGACTTGGCGGTGCTTTATACCCTTCTAGAGGAGCCTGTTCTATAATCGATAAACCCC

702_508Mod AAGGACTTGGCGGTGCTTTATACCCTTCTAGAGGAGCCTGTTCTATAATCGATAAACCCC

Dama_mesopotamica AAGGACTTGGCGGTGCTTTATACCCTTCTAGAGGAGCCTGTTCTATAATCGATAAACCCC

Irish_Elk AAGGACTTGGCGGTGCTTTATACCCTTCTAGAGGAGCCTGTTCTATAATCGATAAACCCC

red_deer AAGGACTTGGCGGTGCTTTATACCCTTCTAGAGGAGCCTGTTCTATAATCGATAAACCCC

****************** * **** **********************************

709_501Mod G-ATAAACCTCACCAT-TACCGCCACCTTCAGCAAACCCTAAAAA-GGTACAAAAGTAAG

702_501Mod G-ATAAACCTCACCAT-TACCGCCACCTTCAGCAAACCCTAAAAA-GGTACAAAAGTAAG

Eemian G-ATAAACCTCACCAC--ACCGCCATCTTCAGCAAACCCTAAAAG-GA---AAAAGTAAG

710_501Mod G-ATAAACCTCACCAT-TACCGCCACCTTCAGCAAACCCTAAAAA-GGTACAAAAGTAAG

706_501Mod G-ATAAACCTCACCAT-TACCGCCACCTTCAGCAAACCCTAAAAA-GGTACAAAAGTAAG

707_501Mod G-ATAAACCTCACCAT-TACCGCCACCTTCAGCAAACCCTAAAAA-GGTACAAAAGTAAG

701_508Mod G-ATAAACCTCACCAT-TACCGCCACCTTCAGCAAACCCTAAAAA-GGTACAAAAGTAAG

712_501Mod G-ATAAACCTCACCAT-TACCGCCACCTTCAGCAAACCCTAAAAA-GGTACAAAAGTAAG

705_501Mod G-ATAAACCTCACCAT-TACCGCCACCTTCAGCAAACCCTAAAAA-GGTACAAAAGTAAG

711_501Mod G-ATAAACCTCACCAT-TACCGCCACCTTCAGCAAACCCTAAAAA-GGTACAAAAGTAAG

702_508Mod G-ATAAACCTCACCAT-TACCGCCACCTTCAGCAAACCCTAAAAA-GGTACAAAAGTAAG

Dama_mesopotamica G-ATAAACCTCACCAT-TACCGCCATCTTCAGCAAACCCTAAAAA-GGTACAAAAGTAAG

Irish_Elk GACTAATACAGTCTATATACCGCCATCTTCAGCAAACCCTAAAAAAGGTACAAAAGTAAG

red_deer --CTAATACAGTCTATATACCGCCATCTTCAGCGAACCCTAAAAA-GGTACAAAAGTAAG

*** * * * ******* ******* ********** * *********

709_501Mod CACAATCATAATACATAAAAACGTTAGGTCAAGGTGTAACCTATGGAATGGGAAGAAATG

702_501Mod CACAATCATAATACATAAAAACGTTAGGTCAAGGTGTAACCTATGGAATGGGAAGAAATG

Eemian CACAATCATGACACATAAAAACGTTAGGTCAAGGTGTAACCTATGGAATGGGAAGAAATG

710_501Mod CACAATCATAATACATAAAAACGTTAGGTCAAGGTGTAACCTATGGAATGGGAAGAAATG

706_501Mod CACAATCATAATACATAAAAACGTTAGGTCAAGGTGTAACCTATGGAATGGGAAGAAATG

707_501Mod CACAATCATAATACATAAAAACGTTAGGTCAAGGTGTAACCTATGGAATGGGAAGAAATG

701_508Mod CACAATCATAATACATAAAAACGTTAGGTCAAGGTGTAACCTATGGAATGGGAAGAAATG

712_501Mod CACAATCATAATACATAAAAACGTTAGGTCAAGGTGTAACCTATGGAATGGGAAGAAATG

705_501Mod CACAATCATAATACATAAAAACGTTAGGTCAAGGTGTAACCTATGGAATGGGAAGAAATG

711_501Mod CACAATCATAATACATAAAAACGTTAGGTCAAGGTGTAACCTATGGAATGGGAAGAAATG

702_508Mod CACAATCATAATACATAAAAACGTTAGGTCAAGGTGTAACCTATGGAATGGGAAGAAATG

Dama_mesopotamica CACAATCATGACACATAAAAACGTTAGGTCAAGGTGTAACCTATGGAACGGGAAGAAATG

Irish_Elk CACAATYATAATACATAAAAACGTTAGGTCAAGGTGTAACCTATGGAACGGAAAGAAATG

red_deer CACAATCATAATACATAAAGACGTTAGGTCAAGGTGTAACCTATGGAACGGAAAGAAATG

****** ** * ******* **************************** ** ********

709_501Mod GGCTACATTTTCTAACCTAAGAAAATCTAATACGAAAGTTATTATGAAACCAGTAACCAA

702_501Mod GGCTACATTTTCTAACCTAAGAAAATCTAATACGAAAGTTATTATGAAACTAGTAACCAA

Eemian GGCTACATTTTCTAACCTAAGAAAATCTAATACGAAAGTTATTATGAAACTAGTAACCAA

710_501Mod GGCTACATTTTCTAACCTAAGAAAATCTAATACGAAAGTTATTATGAAACCAGTAACCAA

706_501Mod GGCTACATTTTCTAACCTAAGAAAATCTAATACGAAAGTTATTATGAAACTAGTAACCAA

707_501Mod GGCTACATTTTCTAACCTAAGAAAATCTAATACGAAAGTTATTATGAAACTAGTAACCAA

701_508Mod GGCTACATTTTCTAACCTAAGAAAATCTAATACGAAAGTTATTATGAAACTAGTAACCAA

712_501Mod GGCTACATTTTCTAACCTAAGAAAATCTAATACGAAAGTTATTATGAAACTAGTAACCAA

705_501Mod GGCTACATTTTCTAACCTAAGAAAATCTAATACGAAAGTTATTATGAAACTAGTAACCAA

711_501Mod GGCTACATTTTCTAACCTAAGAAAATCTAATACGAAAGTTATTATGAAACTAGTAACCAA

702_508Mod GGCTACATTTTCTAACCTAAGAAAATCTAATACGAAAGTTATTATGAAACTAGTAACCAA

Dama_mesopotamica GGCTACATTTTCTAATCCAAGAAAATCCAATACGAAAGTTATTATGAAATTAATAACCAA

Irish_Elk GGCTACATTTTCTAATCTAAGAAAATCCAATACGAAAGTTATTATGAAATTAATAACCAA

red_deer GGCTACATTTTCTAATCTAAGAAAATCCAACACGAAAGTTATTATGAAATTAATAACCAA

*************** * ********* ** ****************** * *******

709_501Mod AGGAGGATTTAGCAGTAAACTAAGAATAGAGTGCTT----AGGCACAATACACTCAAATT

702_501Mod AGGAGGATTTAGCAGTAAACTAAGAATAGAGTGCTT----AGGCACAATACACTCAAATT

Eemian AGGAGGATTTAGCAGTAAACTAAGAATAGAGTGCT-----AGGCACAATATACTCAAATT

710_501Mod AGGAGGATTTAGCAGTAAACTAAGAATAGAGTGCTT----AGGCACAATACACTCAAATT

706_501Mod AGGAGGATTTAGCAGTAAACTAAGAATAGAGTGCTT----AGGCACAATACACTCAAATT

707_501Mod AGGAGGATTTAGCAGTAAACTAAGAATAGAGTGCTT----AGGCACAATACACTCAAATT

701_508Mod AGGAGGATTTAGCAGTAAACTAAGAATAGAGTGCTT----AGGCACAATACACTCAAATT

712_501Mod AGGAGGATTTAGCAGTAAACTAAGAATAGAGTGCTT----AGGCACAATACACTCAAATT

705_501Mod AGGAGGATTTAGCAGTAAACTAAGAATAGAGTGCTT----AGGCACAATACACTCAAATT

711_501Mod AGGAGGATTTAGCAGTAAACTAAGAATAGAGTGCTT----AGGCACAATACACTCAAATT

702_508Mod AGGAGGATTTAGCAGTAAACTAAGAATAGAGTGCTT----AGGCACAATATACTCAAATT

Dama_mesopotamica AGGAGGATTTAGCAGTAAACTAAGAATAGAGTGCT-----AGGCACAATACACTCAAACC

Irish_Elk AGRAGGATTTAGCAGTAAACTAAG-TCACCCTCCTCAAGTAGGCACAATACACTCAAACT

red_deer AGGAGGATTTAGCAGTAAAC-----TCACCCTCCTCAAGTAGGCACAGTACACTCAAATT

** ***************** * * ** ******* ** *******

709_501Mod TATTCATACGTATTAATCACATGAGAGGAGACAAGTCGTAACAAGGTAAGCATACTGGAA

702_501Mod TATTCATACGTATTAATCACATGAGAGGAGACAAGTCGTAACAAGGTAAGCATACTGGAA

Eemian TATTCATACGTATTAATCACATGAGAGGAGACAAGTCGTAACAAGGTAAGCATACTGGAA

710_501Mod TATTCATACGTATTAATCACATGAGAGGAGACAAGTCGTAACAAGGTAAGCATACTGGAA

706_501Mod TATTCATACGTATTAATCACATGAGAGGAGACAAGTCGTAACAAGGTAAGCATACTGGAA

707_501Mod TATTCATACGTATTAATCACATGAGAGGAGACAAGTCGTAACAAGGTAAGCATACTGGAA

701_508Mod TATTCATACGTATTAATCACATGAGAGGAGACAAGTCGTAACAAGGTAAGCATACTGGAA

712_501Mod TATTCATACGTATTAATCACATGAGAGGAGACAAGTCGTAACAAGGTAAGCATACTGGAA

705_501Mod TATTCATACGTATTAATCACATGAGAGGAGACAAGTCGTAACAAGGTAAGCATACTGGAA

711_501Mod TATTCATACGTATTAATCACATGAGAGGAGACAAGTCGTAACAAGGTAAGCATACTGGAA

702_508Mod TATTCATACGTATTAATCACATGAGAGGAGACAAGTCGTAACAAGGTAAGCATACTGGAA

Dama_mesopotamica TATTTACACGTATTAATCATATGAGAGGAGACAAGTCGTAACAAGGTAAGCATACTGGAA

Irish_Elk TATTTATACGTATTAATCAYATGAGAGGAGACAAGTCGTAACAAGGTAAGCATACTGGAA

red_deer TATTTGCACGTATTAATCATATGAGAGGAGACAAGTCGTAACAAGGTAAGCATACTGGAA

**** ************ ****************************************

709_501Mod AGTGTGCTTGGATAAA--TCAAGATATAGCTTAAACAAAGCGCCTAGTCTACACCTAGAA

702_501Mod AGTGTGCTTGGATAAA--TCAAGATATAGCTTAAACAAAGCGCCTAGTCTACACCTAGAA

Eemian AGTGTGCTTGGATAAA--TCAAGATATAGCTTAAACAAAGCGCCTAGTCTACACCTAGAA

710_501Mod AGTGTGCTTGGATAAA--TCAAGATATAGCTTAAACAAAGCGCCTAGTCTACACCTAGAA

706_501Mod AGTGTGCTTGGATAAA--TCAAGATATAGCTTAAACAAAGCGCCTAGTCTACACCTAGAA

707_501Mod AGTGTGCTTGGATAAA--TCAAGATATAGCTTAAACAAAGCGCCTAGTCTACACCTAGAA

701_508Mod AGTGTGCTTGGATAAA--TCAAGATATAGCTTAAACAAAGCGCCTAGTCTACACCTAGAA

712_501Mod AGTGTGCTTGGATAAA--TCAAGATATAGCTTAAACAAAGCGCCTAGTCTACACCTAGAA

705_501Mod AGTGTGCTTGGATAAA--TCAAGATATAGCTTAAACAAAGCGCCTAGTCTACACCTAGAA

711_501Mod AGTGTGCTTGGATAAA--TCAAGATATAGCTTAAACAAAGCGCCTAGTCTACACCTAGAA

702_508Mod AGTGTGCTTGGATAAA--TCAAGATATAGCTTAAACAAAGCGCCTAGTCTACACCTAGAA

Dama_mesopotamica AGTGTGCTTGGATAAA--CCAAGATATAGCTTAAACAAAGCACCTAGTTTACACCTAGAA

Irish_Elk AGTGTGCTTGGATAAA--TCAAGATATAGCTTAAATAAAGCACCTAGTTTACACCTAGAA

red_deer AGTGTGCTTGGATAAAAATCAAGATATAGCTTAAACAAAGCATCTAGTTTACACCTAGAA

**************** **************** ***** ***** ***********

709_501Mod GATTTCACATATTATGAATATCTTGAACTAATTCTAGCCCGAAAAATTAACTAGTAAAAA

702_501Mod GATTTCACATATTATGAATATCTTGAACTAATTCTAGCCCGAAAAATTAACTAGTAAAAA

Eemian GATTTCACATATTATGAATATCTTGAACTAATTCTAGCCCGAAAA-TTAACTAGTAAAAA

710_501Mod GATTTCACATATTATGAATATCTTGAACTAATTCTAGCCCGAAAAATTAACTAGTAAAAA

706_501Mod GATTTCACATATTATGAATATCTTGAACTAATTCTAGCCCGAAAAATTAACTAGTAAAAA

707_501Mod GATTTCACATATTATGAATATCTTGAACTAATTCTAGCCCGAAAA-TTAACTAGTAAAAA

701_508Mod GATTTCACATATTATGAATATCTTGAACTAATTCTAGCCCGAAAA-TTAACTAGTAAAAA

712_501Mod GATTTCACATATTATGAATATCTTGAACTAATTCTAGCCCGAAAA-TTAACTAGTAAAAA

705_501Mod GATTTCACATATTATGAATATCTTGAACTAATTCTAGCCCGAAAAATTAACTAGTAAAAA

711_501Mod GATTTCACATATTATGAATATCTTGAACTAATTCTAGCCCGAAAAATTAACTAGTAAAAA

702_508Mod GATTTCACATATTATGAATATCTTGAACTAATTCTAGCCCGAAAAATTAACTAGTAAAAA

Dama_mesopotamica GATTTCACATATTATGAATATCTTGAACCAATTCTAGCCCGAAAA--TAACTAGTAAAAA

Irish_Elk GATTTCATATATTACGAATATCTTGAACC-------------------------------

red_deer GATTTCATATATCATGAATATCTTGAACAGC-----------------------------

******* **** * *************

709_501Mod CTTAACAAAATGAATTTCAGCTAAGTACCCCGAAACCAGACGAGCTACTTATGAACAATT

702_501Mod CTTAACAAAATGAATTTCAGCTAAGTACCCCGAAACCAGACGAGCTACTTATGAACAATT

Eemian CTTAACAAAATGAATTTCAGCTAAGTACCCCGAAACCAGACGAGCTACTTATGAACAATT

710_501Mod CTTAACAAAATGAATTTCAGCTAAGTACCCCGAAACCAGACGAGCTACTTATGAACAATT

706_501Mod CTTAACAAAATGAATTTCAGCTAAGTACCCCGAAACCAGACGAGCTACTTATGAACAATT

707_501Mod CTTAACAAAATGAATTTCAGCTAAGTACCCCGAAACCAGACGAGCTACTTATGAACAATT

701_508Mod CTTAACAAAATGAATTTCAGCTAAGTACCCCGAAACCAGACGAGCTACTTATGAACAATT

712_501Mod CTTAACAAAATGAATTTCAGCTAAGTACCCCGAAACCAGACGAGCTACTTATGAACAATT

705_501Mod CTTAACAAAATGAATTTCAGCTAAGTACCCCGAAACCAGACGAGCTACTTATGAACAATT

711_501Mod CTTAACAAAATGAATTTCAGCTAAGTACCCCGAAACCAGACGAGCTACTTATGAACAATT

702_508Mod CTTAACAAAATGAATTTCAGCTAAGTACCCCGAAACCAGACGAGCTACTTATGAACAATT

Dama_mesopotamica CTTAACAAAATGAATTTCAGCTAAGTACCCCGAAACCAGACGAGCTACTTATGAACAATT

Irish_Elk -------------ATTTTA-----------------------------------------

red_deer ------------GATTTTA-----------------------------------------

**** *

709_501Mod TATCGAGAACCAACTCATCTATGTAGCAAAATAGTGAGAAGATTTATAAGTAGAGGTGAA

702_501Mod TATCGAGAACCAACTCATCTATGTAGCAAAATAGTGAGAAGATTTATAAGTAGAGGTGAA

Eemian TATCGAGAACCAAC--ATCTATGTAGCAAAATAGTGAGAAGATTTATAAGTAGAGGTGAA

710_501Mod TATCGAGAACCAACTCATCTATGTAGCAAAATAGTGAGAAGATTTATAAGTAGAGGTGAA

706_501Mod TATCGAGAACCAACTCATCTATGTAGCAAAATAGTGAGAAGATTTATAAGTAGAGGTGAA

707_501Mod TATCGAGAACCAACTCATCTATGTAGCAAAATAGTGAGAAGATTTATAAGTAGAGGTGAA

701_508Mod TATCGAGAACCAACTCATCTATGTAGCAAAATAGTGAGAAGATTTATAAGTAGAGGTGAA

712_501Mod TATCGAGAACCAACTCATCTATGTAGCAAAATAGTGAGAAGATTTATAAGTAGAGGTGAA

705_501Mod TATCGAGAACCAACTCATCTATGTAGCAAAATAGTGAGAAGATTTATAAGTAGAGGTGAA

711_501Mod TATCGAGAACCAACTCATCTATGTAGCAAAATAGTGAGAAGATTTATAAGTAGAGGTGAA

702_508Mod TATCGAGAACCAACTCATCTATGTAGCAAAATAGTGAGAAGATTTATAAGTAGAGGTGAA

Dama_mesopotamica TATCGAGAACTAACTCATCTATGTAGCAAAATAGTGAGAAGATTTGTAAGTAGAGGTGAA

Irish_Elk --------------------------------------AAGACT----------------

red_deer --------------------------------------AAGACT----------------

**** *

709_501Mod ACGCCCAACGAGCCTGGTGATAGCTGGTTGTCCAAGACCTACAAGTCGAATCACACAATC

702_501Mod ACGCCCAACGAGCCTGGTGATAGCTGGTTGTCCAAGACCTACAAGTCGAATCACACAATC

Eemian ACGCCCAACGAGCCTGGTGATAGCTGGTTGTCCTAGACTAACCAGTCAAAGTATCACATC

710_501Mod ACGCCCAACGAGCCTGGTGATAGCTGGTTGTCCAAGACCTACAAGTCGAATCACACAATC

706_501Mod ACGCCCAACGAGCCTGGTGATAGCTGGTTGTCCAAGACCTACAAGTCGAATCACACAATC

707_501Mod ACGCCCAACGAGCCTGGTGATAGCTGGTTGTCCAAGACCTACAAGTCGAATCACACAATC

701_508Mod ACGCCCAACGAGCCTGGTGATAGCTGGTTGTCCAAGACCTACAAGTCGAATCACACAATC

712_501Mod ACGCCCAACGAGCCTGGTGATAGCTGGTTGTCCAAGACCTACAAGTCGAATCACACAATC

705_501Mod ACGCCCAACGAGCCTGGTGATAGCTGGTTGTCCAAGACCTACAAGTCGAATCACACAATC

711_501Mod ACGCCCAACGAGCCTGGTGATAGCTGGTTGTCCAAGACCTACAAGTCGAATCACACAATC

702_508Mod ACGCCCAACGAGCCTGGTGATAGCTGGTTGTCCAAGACCTACAAGTCGAATCACACAATC

Dama_mesopotamica ACGCCCAACGAGCCTGGTGATAGCTGGTTGTCC--AACTTACAAGTCGAATCACACAATC

Irish_Elk ----------------------------------AGACCTACAAGTCGAATCACACAATC

red_deer ----------------------------------AGACCTACAAGTCGAATCACACAATC

** ** **** ** * ***

709_501Mod GCTTATTGATCCAAAAAATT-GATCAACGGAACAAGTTACCCTAGGGATAACAGCGCAAT

702_501Mod GCTTATTGATCCAAAAAATT-GATCAACGGAACAAGTTACCCTAGGGATAACAGCGCAAT

Eemian ACTTATTGATCCAAAAATTTTGATCAACGGAACAAGTTACCCTAGGGATAACAGCGCAAT

710_501Mod GCTTATTGATCCAAAAAATT-GATCAACGGAACAAGTTACCCTAGGGATAACAGCGCAAT

706_501Mod GCTTATTGATCCAAAAAATT-GATCAACGGAACAAGTTACCCTAGGGATAACAGCGCAAT

707_501Mod GCTTATTGATCCAAAAAATT-GATCAACGGAACAAGTTACCCTAGGGATAACAGCGCAAT

701_508Mod GCTTATTGATCCAAAAAATT-GATCAACGGAACAAGTTACCCTAGGGATAACAGCGCAAT

712_501Mod GCTTATTGATCCAAAAAATT-GATCAACGGAACAAGTTACCCTAGGGATAACAGCGCAAT

705_501Mod GCTTATTGATCCAAAAAATT-GATCAACGGAACAAGTTACCCTAGGGATAACAGCGCAAT

711_501Mod GCTTATTGATCCAAAAAATT-GATCAACGGAACAAGTTACCCTAGGGATAACAGCGCAAT

702_508Mod GCTTATTGATCCAAAAAATT-GATCAACGGAACAAGTTACCCTAGGGATAACAGCGCAAT

Dama_mesopotamica GCTTATTGATCCAAAAAATT-GATCAACGGAACAAGTTACCCTAGGGATAACAGCGCAAT

Irish_Elk GCTTATTGATCCAAAAAATT-GATCAACGGAACAAGTTACCCTAGGGATAACAGCGCAAT

red_deer GCTTATTGATCCAAAAAATT-GATCAACGGAACAAGTTACCCTAGGGATAACAGCGCAAT

**************** ** ***************************************

709_501Mod CCTATTCAAGAGTCCATATCGACAATAGGGTTTACGACCTCGATGTTGGATCAGGACATC

702_501Mod CCTATTCAAGAGTCCATATCGACAATAGGGTTTACGACCTCGATGTTGGATCAGGACATC

Eemian CCTATTCAAGAGTCCATATCGACAATAGGGTTTACGACCTCGATGTTGGATCAGGACATC

710_501Mod CCTATTCAAGAGTCCATATCGACAATAGGGTTTACGACCTCGATGTTGGATCAGGACATC

706_501Mod CCTATTCAAGAGTCCATATCGACAATAGGGTTTACGACCTCGATGTTGGATCAGGACATC

707_501Mod CCTATTCAAGAGTCCATATCGACAATAGGGTTTACGACCTCGATGTTGGATCAGGACATC

701_508Mod CCTATTCAAGAGTCCATATCGACAATAGGGTTTACGACCTCGATGTTGGATCAGGACATC

712_501Mod CCTATTCAAGAGTCCATATCGACAATAGGGTTTACGACCTCGATGTTGGATCAGGACATC

705_501Mod CCTATTCAAGAGTCCATATCGACAATAGGGTTTACGACCTCGATGTTGGATCAGGACATC

711_501Mod CCTATTCAAGAGTCCATATCGACAATAGGGTTTACGACCTCGATGTTGGATCAGGACATC

702_508Mod CCTATTCAAGAGTCCATATCGACAATAGGGTTTACGACCTCGATGTTGGATCAGGACATC

Dama_mesopotamica CCTATTCAAGAGTCCCTATCGACAATAGGGTTTACGACCTCGATGTTGGATCAGGACATC

Irish_Elk CCTATTCAAGAGTCCTTATCGACAATAGGGTTTACGACCTCGATGTTGGATCAGGACATC

red_deer CCTATTCAAGAGTCCATATCGACAATAGGGTTTACGACCTCGATGTTGGATCAGGACATC

*************** ********************************************

709_501Mod CCGATGGTGCAACCGCTATCAAAGGTTCGTTTGTTCAACGATTAAAGTCCTACGTGATCT

702_501Mod CCGATGGTGCAACCGCTATCAAAGGTTCGTTTGTTCAACGATTAAAGTCCTACGTGATCT

Eemian CTAATGGTGCAGCAGCTATTAAGGGTTCGTTTGTTCAACGATTAAAGTCCTACGTGATCT

710_501Mod CCGATGGTGCAACCGCTATCAAAGGTTCGTTTGTTCAACGATTAAAGTCCTACGTGATCT

706_501Mod CCGATGGTGCAACCGCTATCAAAGGTTCGTTTGTTCAACGATTAAAGTCCTACGTGATCT

707_501Mod CCGATGGTGCAACCGCTATCAAAGGTTCGTTTGTTCAACGATTAAAGTCCTACGTGATCT

701_508Mod CCGATGGTGCAACCGCTATCAAAGGTTCGTTTGTTCAACGATTAAAGTCCTACGTGATCT

712_501Mod CCGATGGTGCAACCGCTATCAAAGGTTCGTTTGTTCAACGATTAAAGTCCTACGTGATCT

705_501Mod CCGATGGTGCAACCGCTATCAAAGGTTCGTTTGTTCAACGATTAAAGTCCTACGTGATCT

711_501Mod CCGATGGTGCAACCGCTATCAAAGGTTCGTTTGTTCAACGATTAAAGTCCTACGTGATCT

702_508Mod CCGATGGTGCAACCGCTATCAAAGGTTCGTTTGTTCAACGATTAAAGTCCTACGTGATCT

Dama_mesopotamica CCGATGGTGCAACCGCTATCAAAGGTTCGTTTGTTCAACGATTAAAGTCCTACGTGATCT

Irish_Elk CCGATGGTGCAACCGCTATCAAAGGTTCGTTTGTTCAACGATTAAAGTCTGGGGCCCTAC

red_deer CCGATGGTGCAACCGCTATCAAAGGTTCGTTTGTTCAACGATTAATAATCGGGGCCCTGC

* ******** * ***** ** ********************** * *

709_501Mod GAGAATGATATCATCTTAATTAACTTCACAAACAAATCTTGCCCTAGAAAAGGGCCTTGT

702_501Mod GAGAATGATATCATCTTAATTAACTTCACAAACAAATCTTGCCCTAGAAAAGGGCCTTGT

Eemian GA-AATGATATCATCTTAATTAACTTCACAAACAAATCTTGCCCTAGAAAAGGGCCTTGT

710_501Mod GAGAATGATATCATCTTAATTAACTTCACAAACAAATCTTGCCCTAGAAAAGGGCCTTGT

706_501Mod GAGAATGATATCATCTTAATTAACTTCACAAACAAATCTTGCCCTAGAAAAGGGCCTTGT

707_501Mod GAGAATGATATCATCTTAATTAACTTCACAAACAAATCTTGCCCTAGAAAAGGGCCTTGT

701_508Mod GAGAATGATATCATCTTAATTAACTTCACAAACAAATCTTGCCCTAGAAAAGGGCCTTGT

712_501Mod GAGAATGATATCATCTTAATTAACTTCACAAACAAATCTTGCCCTAGAAAAGGGCCTTGT

705_501Mod GAGAATGATATCATCTTAATTAACTTCACAAACAAATCTTGCCCTAGAAAAGGGCCTTGT

711_501Mod GAGAATGATATCATCTTAATTAACTTCACAAACAAATCTTGCCCTAGAAAAGGGCCTTGT

702_508Mod GAGAATGATATCATCTTAATTAACTTCACAAACAAATCTTGCCCTAGAAAAGGGCCTTGT

Dama_mesopotamica GA----------------------------------------------------------

Irish_Elk GA----------------------------------------------------------

red_deer GA----------------------------------------------------------

**

709_501Mod TAAGGTGGCAGAGCCGCGGTAGCACAAACAATTTCATATGAAGTAACACTAGCAATTATC

702_501Mod TAAGGTGGCAGAGCCGCGGTAGCACAAACAATTTCATATGAAGTAACACTAGCAATTATC

Eemian TAAGGTGGCAGAGC-GCGGTAGCACAAACAATTTCATATGAAGTAACACTAGCAATTATC

710_501Mod TAAGGTGGCAGAGCCGCGGTAGCACAAACAATTTCATATGAAGTAACACTAGCAATTATC

706_501Mod TAAGGTGGCAGAGCCGCGGTAGCACAAACAATTTCATATGAAGTAACACTAGCAATTATC

707_501Mod TAAGGTGGCAGAGCCGCGGTAGCACAAACAATTTCATATGAAGTAACACTAGCAATTATC

701_508Mod TAAGGTGGCAGAGCCGCGGTAGCACAAACAATTTCATATGAAGTAACACTAGCAATTATC

712_501Mod TAAGGTGGCAGAGCCGCGGTAGCACAAACAATTTCATATGAAGTAACACTAGCAATTATC

705_501Mod TAAGGTGGCAGAGCCGCGGTAGCACAAACAATTTCATATGAAGTAACACTAGCAATTATC

711_501Mod TAAGGTGGCAGAGCCGCGGTAGCACAAACAATTTCATATGAAGTAACACTAGCAATTATC

702_508Mod TAAGGTGGCAGAGCCGCGGTAGCACAAACAATTTCATATGAAGTAACACTAGCAATTATC

Dama_mesopotamica ---------------GCGGTAGCACAAACAATTTCATACGAAGTAACACTAGCAATTATC

Irish_Elk ---------------GCAGTAGCACAAACAATTTCATATGAAGTAACACTAGCAATCATT

red_deer ---------------GCAGTAGCACAAACAATTTCATATGAAGTAACACTAGCAATTATT

** ******************** ***************** **

709_501Mod CTACTATCCATCCTCTTAATAAACGGATCCTTTACACTCTCTACCCTAATTATTACACAA

702_501Mod CTACTATCCATCCTCTTAATAAACGGATCCTTTACACTCTCTACCCTAATTATTACACAA

Eemian CTACTATCCATCCTCTTAATAAACGGATCCTTTACACTCTCTACCCTAATTATTACACAA

710_501Mod CTACTATCCATCCTCTTAATAAACGGATCCTTTACACTCTCTACCCTAATTATTACACAA

706_501Mod CTACTATCCATCCTCTTAATAAATGGATCCTTTACACTCTCTACCCTAATTATTACACAA

707_501Mod CTACTATCCATCCTCTTAATAAATGGATCCTTTACACTCTCTACCCTAATTATTACACAA

701_508Mod CTACTATCCATCCTCTTAATAAACGGATCCTTTACACTCTCTACCCTAATTATTACACAA

712_501Mod CTACTATCCATCCTCTTAATAAACGGATCCTTTACACTCTCTACCCTAATTATTACACAA

705_501Mod CTACTATCCATCCTCTTAATAAATGGATCCTTTACACTCTCTACCCTAATTATTACACAA

711_501Mod CTACTATCCATCCTCTTAATAAACGGATCCTTTACACTCTCTACCCTAATTATTACACAA

702_508Mod CTACTATCCATCCTCTTAATAAACGGATCCTTTACACTCTCTACCCTAATTATTACACAA

Dama_mesopotamica CTATTGTCTATTCTCCTAATAAACGGGTCCTTTACACTTTCCACTTTAATCATTACACAA

Irish_Elk CTACTATCAGTCCTCCTAATAAACGGATCCTTTACACTCTCCACTTTAATTATCACACAA

red_deer CTACTATCCGTCCTCCTAATAAATGGGTCCTTTACACTCTCCACCTTAATTATTACACAA

*** * ** * *** ******* ** *********** ** ** **** ** ******

709_501Mod GAACAAGTATGACTTATTTTCCCAGCATGGCCCCTGGCAATAATATGATTTATCTCAACA

702_501Mod GAACAAGTATGACTTATTTTCCCAGCATGGCCCCTGGCAATAATATGATTTATCTCAACA

Eemian GAACAAGTATGACTTATTTTCCCAGCATGGCCCCTGGCAATAATATGATTTATCTCAACA

710_501Mod GAACAAGTATGACTTATTTTCCCAGCATGGCCCCTGGCAATAATATGATTTATCTCAACA

706_501Mod GAACAAGTATGACTTATTTTCCCAGCATGGCCCCTGGCAATAATATGATTTATCTCAACA

707_501Mod GAACAAGTATGACTTATTTTCCCAGCATGGCCCCTGGCAATAATATGATTTATCTCAACA

701_508Mod GAACAAGTATGACTTATTTTCCCAGCATGGCCCCTGGCAATAATATGATTTATCTCAACA

712_501Mod GAACAAGTATGACTTATTTTCCCAGCATGGCCCCTGGCAATAATATGATTTATCTCAACA

705_501Mod GAACAAGTATGACTTATTTTCCCAGCATGGCCCCTGGCAATAATATGATTTATCTCAACA

711_501Mod GAACAAGTATGACTTATTTTCCCAGCATGGCCCCTGGCAATAATATGATTTATCTCAACA

702_508Mod GAACAAGTATGACTTATTTTCCCAGCATGGCCCCTGGCAATAATATGATTTATCTCAACA

Dama_mesopotamica GAACAAGTATGACTTATTTTCCCAGCATGACCCCTAGCAATAATATGATTCATCTCAACG

Irish_Elk GAACAAGTATGACTTATCTTCCCAGCATGANNNNNNNTAATAATATGATTTATCTCAACA

red_deer GAACAAGTATGGCTCATCTTCCCGGCATGACCCCTAGCAATAATGTGATTCATCTCAACA

*********** ** ** ***** ***** ****** ***** ********

709_501Mod CTAGCAGAAACAAACCGGGCTCCATTTGACCTCACCGAGGGCGA-ATCAGAATTCTTTAC

702_501Mod CTAGCAGAAACAAACCGGGCTCCATTTGACCTCACCGAGGGCGA-ATCAGAATTCTTTAC

Eemian CTAGCAGAAACAAACCGGGCTCCATTTGACCTCACCGAGGGCGA-ATCAGA--TTTTTAC

710_501Mod CTAGCAGAAACAAACCGGGCTCCATTTGACCTCACCGAGGGCGA-ATCAGAATTCTTTAC

706_501Mod CTAGCAGAAACAAACCGGGCTCCATTTGACCTCACCGAGGGCGA-ATCAGAATTCTTTAC

707_501Mod CTAGCAGAAACAAACCGGGCTCCATTTGACCTCACCGAGGGCGA-ATCAGAATTCTTTAC

701_508Mod CTAGCAGAAACAAACCGGGCTCCATTTGACCTCACCGAGGGCGA-ATCAGAATTCTTTAC

712_501Mod CTAGCAGAAACAAACCGGGCTCCATTTGACCTCACCGAGGGCGA-ATCAGAATTCTTTAC

705_501Mod CTAGCAGAAACAAACCGGGCTCCATTTGACCTCACCGAGGGCGA-ATCAGAATTCTTTAC

711_501Mod CTAGCAGAAACAAACCGGGCTCCATTTGACCTCACCGAGGGCGA-ATCAGAATTCTTTAC

702_508Mod CTAGCAGAAACAAACCGGGCTCCATTTGACCTCACCGAGGGCGA-ATCAGAATTCTTTAC

Dama_mesopotamica TTAGCAGAAACAAACCGGGCCCCATTTGACCTTACCGAAGGTGA-ATCAGAA-TTTCTAC

Irish_Elk CTAGCAGAAACAAATCGGGCCCCATTCGACCTCACCGAAAATTTTACCAGAA--CTCTAC

red_deer CTAGCAGAAACAAACCGAGCCCCATTTGATCTCACACCCAGTCCTACCAGAA--CTTTAC

************* ** ** ***** ** ** ** * **** * ***

709_501Mod ACAATCAATTTTACCATTAAATCTTTACTACTAACAATCTCCTTCTTATGAATCCGAGCA

702_501Mod ACAATCAATTTTACCATTAAATCTTTACTACTAACAATCTCCTTCTTATGAATCCGAGCA

Eemian ACAATCAATTTTACCATTAAATCTTTACTACTAACAATCTCCTTCTTATGAATCCGAGCA

710_501Mod ACAATCAATTTTACCATTAAATCTTTACTACTAACAATCTCCTTCTTATGAATCCGAGCA

706_501Mod ACAATCAATTTTACCATTAAATCTTTACTACTAACAATCTCCTTCTTATGAATCCGAGCA

707_501Mod ACAATCAATTTTACCATTAAATCTTTACTACTAACAATCTCCTTCTTATGAATCCGAGCA

701_508Mod ACAATCAATTTTACCATTAAATCTTTACTACTAACAATCTCCTTCTTATGAATCCGAGCA

712_501Mod ACAATCAATTTTACCATTAAATCTTTACTACTAACAATCTCCTTCTTATGAATCCGAGCA

705_501Mod ACAATCAATTTTACCATTAAATCTTTACTACTAACAATCTCCTTCTTATGAATCCGAGCA

711_501Mod ACAATCAATTTTACCATTAAATCTTTACTACTAACAATCTCCTTCTTATGAATCCGAGCA

702_508Mod ACAATCAATTTTACCATTAAATCTTTACTACTAACAATCTCCTTCTTATGAATCCGAGCA

Dama_mesopotamica ACAATCAATTTTACTATTAAATCTCTACTACTAACGATCTCCTTCTTATGAATCCGAGCA

Irish_Elk ACAATCAACTTTACCATTAAATCCCTACTACTGACAATCTCCTTCCTATGAATCCGAGCA

red_deer ACAATCAATTTTATCATAAAATCCCTACTATTAACAATTTCCTTCTTATGAATCCGAGCA

******** **** ** ***** ***** * ** ** ****** **************

709_501Mod TCCTATCCTCGATTTCGCTACGACCAACTAATACACCTATTATGAAAAAACTTTTTACCC

702_501Mod TCCTATCCTCGATTTCGCTACGACCAACTAATACACCTATTATGAAAAAACTTTTTACCC

Eemian TCCTATCCTCGATTTCGCTACGACCAACTAATACACCTATTATGAAAAAACTTTTTACCC

710_501Mod TCCTATCCTCGATTTCGCTACGACCAACTAATACACCTATTATGAAAAAACTTTTTACCC

706_501Mod TCCTATCCTCGATTTCGCTACGACCAACTAATACACCTATTATGAAAAAACTTTTTACCC

707_501Mod TCCTATCCTCGATTTCGCTACGACCAACTAATACACCTATTATGAAAAAACTTTTTACCC

701_508Mod TCCTATCCTCGATTTCGCTACGACCAACTAATACACCTATTATGAAAAAACTTTTTACCC

712_501Mod TCCTATCCTCGATTTCGCTACGACCAACTAATACACCTATTATGAAAAAACTTTTTACCC

705_501Mod TCCTATCCTCGATTTCGCTACGACCAACTAATACACCTATTATGAAAAAACTTTTTACCC

711_501Mod TCCTATCCTCGATTTCGCTACGACCAACTAATACACCTATTATGAAAAAACTTTTTACCC

702_508Mod TCCTATCCTCGATTTCGCTACGACCAACTAATACACCTATTATGAAAAAACTTTTTACCC

Dama_mesopotamica TCTTATCCTCGATTTCGCTATGACCAACTGATACACCTACTATGAAAAAATTTTCTACCC

Irish_Elk TCCTATCCTCGATTTCGCTACGACCAACTAATACACCTACTATGAAAAAATTTTTTACTA

red_deer TCTTACCCTCGATTCCGTTATGACCAACTAATACACCTATTATGAAAAAATTTTATACTA

** ** ******** ** ** ******** ********* ********** *** ***

709_501Mod TTGACATTAGCCCGTCATTATTAACCTAATATTTTCAGGCCAATGAACTGTAATAAAACT

702_501Mod TTGACATTAGCCCGTCATTATTAACCTAATATTTTCAGGCCAATGAACTGTAATAAAACT

Eemian TTGACATTAGCC-GTCATTATTAACCTAATATTTTCAGGCCAATGAACTGTAATAAAACT

710_501Mod TTGACATTAGCCCGTCATTATTAACCTAATATTTTCAGGCCAATGAACTGTAATAAAACT

706_501Mod TTGACATTAGCCCGTCATTATTAACCTAATATTTTCAGGCCAATGAACTGTAATAAAACT

707_501Mod TTGACATTAGCCCGTCATTATTAACCTAATATTTTCAGGCCAATGAACTGTAATAAAACT

701_508Mod TTGACATTAGCCCGTCATTATTAACCTAATATTTTCAGGCCAATGAACTGTAATAAAACT

712_501Mod TTGACATTAGCCCGTCATTATTAACCTAATATTTTCAGGCCAATGAACTGTAATAAAACT

705_501Mod TTGACATTAGCCCGTCATTATTAACCTAATATTTTCAGGCCAATGAACTGTAATAAAACT

711_501Mod TTGACATTAGCCCGTCATTATTAACCTAATATTTTCAGGCCAATGAACTGTAATAAAACT

702_508Mod TTGACATTAGCCCGTCATTATTAACCTAATATTTTCAGGCCAATGAACTGTAATAAAACT

Dama_mesopotamica TTAACATTAGCC-GTTATTATTAACCTAATATTTTCAGGCCAATGAACTGTAATAAAACT

Irish_Elk CTAATAATAGCC-GTCATTATTAACCTAATATTTTCAGGCCAATGAACCGTAATAAAACT

red_deer CTAATAATAGCC-ATTATTATTAACTTAATATTTTCAGGCCAATGAACTGTAATAAAACT

* * * ***** * ********* ********************** ***********

709_501Mod ATTTAATCCAGTGGCATCCATACTCATAACAATAGCTCTCACTATAAAACTAGGAATAGC

702_501Mod ATTTAATCCAGTGGCATCCATACTCATAACAATAGCTCTCACTATAAAACTAGGAATAGC

Eemian ATTTAATCCAGTGGCATCCATACTCATAACAATAGCTCTCACTATAAAACTAGGAATAGC

710_501Mod ATTTAATCCAGTGGCATCCATACTCATAACAATAGCTCTCACTATAAAACTAGGAATAGC

706_501Mod ATTTAATCCAGTGGCATCCATACTCATAACAATAGCTCTCACTATAAAACTAGGAATAGC

707_501Mod ATTTAATCCAGTGGCATCCATACTCATAACAATAGCTCTCACTATAAAACTAGGAATAGC

701_508Mod ATTTAATCCAGTGGCATCCATACTCATAACAATAGCTCTTACTATAAAACTAGGAATAGC

712_501Mod ATTTAATCCAGTGGCATCCATACTCATAACAATAGCTCTCACTATAAAACTAGGAATAGC

705_501Mod ATTTAATCCAGTGGCATCCATACTCATAACAATAGCTCTCACTATAAAACTAGGAATAGC

711_501Mod ATTTAATCCAGTGGCATCCATACTCATAACAATAGCTCTCACTATAAAACTAGGAATAGC

702_508Mod ATTTAATCCAGTGGCATCCATACTCATAACAATAGCTCTCACTATAAAACTAGGAATAGC

Dama_mesopotamica ATTTAATCCAACGGCATCTATACTCATAACAATGGCTCTCACTATAAAACTAGGAATAGC

Irish_Elk ATTCAACCCAGTAGCGTCTATACTTATAACAATGGCTCTCACCATAAAACTAGGAATAGC

red_deer ATTTAACCCAGTAGCATCTATATTCATAACAATGGCTCTCACTATAAAACTAGGAATAGT

*** ** *** ** ** *** * ******** ***** ** ****************

709_501Mod CCCATTTCACTTCTGACTCTCTACCAAATTTTCCCATCCATTAACCTAAACATGATCCTA

702_501Mod CCCATTTCACTTCTGACTCTCTACCAAATTTTCCCATCCATTAACCTAAACATGATCCTA

Eemian CCCATTTCACTTCTGA-TCTCTACCAAATTTTCCCATCCATTAACCTAAACATGATCCTA

710_501Mod CCCATTTCACTTCTGACTCTCTACCAAATTTTCCCATCCATTAACCTAAACATGATCCTA

706_501Mod CCCATTTCACTTCTGACTCTCTACCAAATTTTCCCATCCATTAACCTAAACATGATCCTA

707_501Mod CCCATTTCACTTCTGACTCTCTACCAAATTTTCCCATCCATTAACCTAAACATGATCCTA

701_508Mod CCCATTTCACTTCTGACTCTCTACCAAATTTTCCCATCCATTAACCTAAACATGATCCTA

712_501Mod CCCATTTCACTTCTGACTCTCTACCAAATTTTCCCATCCATTAACCTAAACATGATCCTA

705_501Mod CCCATTTCACTTCTGACTCTCTACCAAATTTTCCCATCCATTAACCTAAACATGATCCTA

711_501Mod CCCATTTCACTTCTGACTCTCTACCAAATTTTCCCATCCATTAACCTAAACATGATCCTA

702_508Mod CCCATTTCACTTCTGACTCTCTACCAAATTTTCCCATCCATTAACCTAAACATGATCCTA

Dama_mesopotamica CCCATTCCATTTTTGA-GCTCTACCAAATCTCTTCATCCATCAATCTAAATATAATTCTA

Irish_Elk CCCCACCCATATCCGT-TCTCTACCAAATTTCTCCATCCATTAATCTAAATATAATCTTA

red_deer TAGCACCCATATCCGT-TCTTTACCAAATTTTTCCATCTATCAATTTAAATATAATTTTA

** * * ** ******** * **** ** ** **** ** ** **

709_501Mod ACCATCTCCATTCTATCAATCATAATTGGAGGTTGAGGAGGACTAAACCAAACCCAGCTA

702_501Mod ACCATCTCCATTCTATCAATCATAATTGGAGGTTGAGGAGGACTAAACCAAACCCAGCTA

Eemian ACCATCTCCATTCTATCAATCATAATTGGAGGTTGAGGAGGACTAAACCAAACCCAGCTA

710_501Mod ACCATCTCCATTCTATCAATCATAATTGGAGGTTGAGGAGGACTAAACCAAACCCAGCTA

706_501Mod ACCATCTCCATTCTATCAATCATAATTGGAGGTTGAGGAGGACTAAACCAAACCCAGCTA

707_501Mod ACCATCTCCATTCTATCAATCATAATTGGAGGTTGAGGAGGACTAAACCAAACCCAGCTA

701_508Mod ACCATCTCCATTCTATCAATCATAATTGGAGGTTGAGGAGGACTAAACCAAACCCAGCTA

712_501Mod ACCATCTCCATTCTATCAATCATAATTGGAGGTTGAGGAGGACTAAACCAAACCCAGCTA

705_501Mod ACCATCTCCATTCTATCAATCATAATTGGAGGTTGAGGAGGACTAAACCAAACCCAGCTA

711_501Mod ACCATCTCCATTCTATCAATCATAATTGGAGGTTGAGGAGGACTAAACCAAACCCAGCTA

702_508Mod ACCATCTCCATTCTATCAATCATAATTGGAGGTTGAGGAGGACTAAACCAAACCCAGCTA

Dama_mesopotamica ACCATTTCCATTTTATCAATCATAATTGGAGGCTGAGGAGGACTAAACCAAACCCAACTA

Irish_Elk ACCATTTCCATCTTATCAATCATAATTGGAGGCTGAGGAGGACTAAACCAAACCCAACTA

red_deer ACCATTTCCATTTTATCAATCATAATTGGGGGCTGAGGAGGGCTAAACCAAACTCAACTA

***** ***** **************** ** ******** *********** ** ***

709_501Mod CGAAAAATTATAGCATATTCATCAATTGCTCATATGGGTTAATTATTCAAGAAATAACAA

702_501Mod CGAAAAATTATAGCATATTCATCAATTGCTCATATGGGTTAATTATTCAAGAAATAACAA

Eemian CGAAAAATTATAGCATATTCATCAATTGCCCATATGGGT-AATTATTCAAGAAATAACAA

710_501Mod CGAAAAATTATAGCATATTCATCAATTGCTCATATGGGTTAATTATTCAAGAAATAACAA

706_501Mod CGAAAAATTATAGCATATTCATCAATTGCCCATATGGGTTAATTATTCAAGAAATAACAA

707_501Mod CGAAAAATTATAGCATATTCATCAATTGCCCATATGGGTTAATTATTCAAGAAATAACAA

701_508Mod CGAAAAATTATAGCATATTCATCAATTGCCCATATGGGTTAATTATTCAAGAAATAACAA

712_501Mod CGAAAAATTATAGCATATTCATCAATTGCCCATATGGGTTAATTATTCAAGAAATAACAA

705_501Mod CGAAAAATTATAGCATATTCATCAATTGCCCATATGGGTTAATTATTCAAGAAATAACAA

711_501Mod CGAAAAATTATAGCATATTCATCAATTGCCCATATGGGTTAATTATTCAAGAAATAACAA

702_508Mod CGAAAAATTATAGCATATTCATCAATTGCCCATATGGGTTAATTATTCAAGAAATAACAA

Dama_mesopotamica CGAAAAATTATAGCATATTCATCAATTGCTCACATAGGCTA-------------------

Irish_Elk CGAAAAATCATAGCATATTCATCAATTGCTCACA--------------------------

red_deer CGAAAAATTATGGCATATTCATGA---------G--------------------------

******** ** ********** *

709_501Mod AAAATGACAGCCTCATCCTACCCACTCTCATAGCAATCACAGCACTACTAAATTTATATT

702_501Mod AAAATGACAGCCTCATCCTACCCACTCTCATAGCAATCACAGCACTACTAAATTTATATT

Eemian AAAATGACAGCCTCATCCTACCCACTCTCATAGCAATCACAGCACTACTAAATTTATATT

710_501Mod AAAATGACAGCCTCATCCTACCCACTCTCATAGCAATCACAGCACTACTAAATTTATATT

706_501Mod AAAATGACAGCCTCATCCTACCCACTCTCATAGCAATCACAGCACTACTAAATTTATATT

707_501Mod AAAATGACAGCCTCATCCTACCCACTCTCATAGCAATCACAGCACTACTAAATTTATATT

701_508Mod AAAATGACAGCCTCATCCTACCCACTCTCATAGCAATCACAGCACTACTAAATTTATATT

712_501Mod AAAATGACAGCCTCATCCTACCCACTCTCATAGCAATCACAGCACTACTAAATTTATATT

705_501Mod AAAATGACAGCCTCATCCTACCCACTCTCATAGCAATCACAGCACTACTAAATTTATATT

711_501Mod AAAATGACAGCCTCATCCTACCCACTCTCATAGCAATCACAGCACTACTAAATTTATATT

702_508Mod AAAATGACAGCCTCATCCTACCCACTCTCATAGCAATCACAGCACTACTAAATTTATATT

Dama_mesopotamica ------------------------------------------------------------

Irish_Elk ------------------------------------------------------------

red_deer ------------------------------------------------------------

709_501Mod TCTATATACGACTCACATATTCCACCGCACTAACAATATTTCCCTTAACTCCTGATAAGG

702_501Mod TCTATATACGACTCACATATTCCACCGCACTAACAATATTTCCCTTAACTCCTGATAAGG

Eemian TCTATATACGACTCACATATTCCACCGCACTAACAATATTTCCCT-AACTCCTGATAAGG

710_501Mod TCTATATACGACTCACATATTCCACCGCACTAACAATATTTCCCTTAACTCCTGATAAGG

706_501Mod TCTATATACGACTCACATATTCCACCGCACTAACAATATTTCCCTTAACTCCTGATAAGG

707_501Mod TCTATATACGACTCACATATTCCACCGCACTAACAATATTTCCCTTAACTCCTGATAAGG

701_508Mod TCTATATACGACTCACATATTCCACCGCACTAACAATATTTCCCTTAACTCCTGATAAGG

712_501Mod TCTATATACGACTCACATATTCCACCGCACTAACAATATTTCCCTTAACTCCTGATAAGG

705_501Mod TCTATATACGACTCACATATTCCACCGCACTAACAATATTTCCCTTAACTCCTGATAAGG

711_501Mod TCTATATACGACTCACATATTCCACCGCACTAACAATATTTCCCTTAACTCCTGATAAGG

702_508Mod TCTATATACGACTCACATATTCCACCGCACTAACAATATTTCCCTTAACTCCTGATAAGG

Dama_mesopotamica ------------------------------------------------CTCCTGATAAGG

Irish_Elk ------------------------------------------------------------

red_deer ------------------------------------------------------------

709_501Mod ATTGCAAGACCATATCTTACATCAATTGAATGCAAATCAACCACTTTAATTAAGCTAAAT

702_501Mod ATTGCAAGACCATATCTTACATCAATTGAATGCAAATCAACCACTTTAATTAAGCTAAAT

Eemian ATTGCAAGACCATATCTTACATCAATTGAATGCAAATCAACCACTTTAATTAAGCTAAAT

710_501Mod ATTGCAAGACCATATCTTACATCAATTGAATGCAAATCAACCACTTTAATTAAGCTAAAT

706_501Mod ATTGCAAGACCATATCTTACATCAATTGAATGCAAATCAACCACTTTAATTAAGCTAAAT

707_501Mod ATTGCAAGACCATATCTTACATCAATTGAATGCAAATCAACCACTTTAATTAAGCTAAAT

701_508Mod ATTGCAAGACCATATCTTACATCAATTGAATGCAAATCAACCACTTTAATTAAGCTAAAT

712_501Mod ATTGCAAGACCATATCTTACATCAATTGAATGCAAATCAACCACTTTAATTAAGCTAAAT

705_501Mod ATTGCAAGACCATATCTTACATCAATTGAATGCAAATCAACCACTTTAATTAAGCTAAAT

711_501Mod ATTGCAAGACCATATCTTACATCAATTGAATGCAAATCAACCACTTTAATTAAGCTAAAT

702_508Mod ATTGCAAGACCATATCTTACATCAATTGAATGCAAATCAACCACTTTAATTAAGCTAAAT

Dama_mesopotamica ATTGCAAGACCACATCTTACATCAATTGAATGCAAATCAACCACTTTAATTAAGCTAAAT

Irish_Elk ---------------------------TAATATAAA----CCTCTGT-------------

red_deer ---------------------------GAATACAAA----CCTCTGT-------------

*** *** ** ** *

709_501Mod CCTCACTAGATTGGTGGGCTCCACCCCCACGAAACTTTAGATTTACAGTCTAATGCTTCA

702_501Mod CCTCACTAGATTGGTGGGCTCCACCCCCACGAAACTTTAGATTTACAGTCTAATGCTTCA

Eemian CCTCACTAGATTGGTGG-----------------CTTTAGATTTACAGTCTAATGCTTCA

710_501Mod CCTCACTAGATTGGTGGGCTCCACCCCCACGAAACTTTAGATTTACAGTCTAATGCTTCA

706_501Mod CCTCACTAGATTGGTGGGCTCCACCCCCACGAAACTTTAGATTTACAGTCTAATGCTTCA

707_501Mod CCTCACTAGATTGGTGGGCTCCACCCCCACGAAACTTTAGATTTACAGTCTAATGCTTCA

701_508Mod CCTCACTAGATTGGTGGGCTCCACCCCCACGAAACTTTAGATTTACAGTCTAATGCTTCA

712_501Mod CCTCACTAGATTGGTGGGCTCCACCCCCACGAAACTTTAGATTTACAGTCTAATGCTTCA

705_501Mod CCTCACTAGATTGGTGGGCTCCACCCCCACGAAACTTTAGATTTACAGTCTAATGCTTCA

711_501Mod CCTCACTAGATTGGTGGGCTCCACCCCCACGAAACTTTAGATTTACAGTCTAATGCTTCA

702_508Mod CCTCACTAGATTGGTGGGCTCCACCCCCACGAAACTTTAGATTTACAGTCTAATGCTTCA

Dama_mesopotamica CCTCACTAGATTGGTGGGTTCCACCCCCACGAAACTTTAGATTTACAGTCTAATGCTTCA

Irish_Elk ----------------------------------CTTTAGATTTACAGTCTAATGCTTCA

red_deer ----------------------------------CTTTAGATTTACAGTCTAATGCTTCA

**************************

709_501Mod -CTCAGCCATTCTACCTATGTTCATTAACCGCTGATTGTTCTCAACCAACCATAAAGATA

702_501Mod -CTCAGCCATTCTACCTATGTTCATTAACCGCTGATTGTTCTCAACCAACCATAAAGATA

Eemian -CTCAGCCATTCTACCTATGTTCATTAACCGCTGATTGTTCTCAACCAACCATAAAGATA

710_501Mod -CTCAGCCATTCTACCTATGTTCATTAACCGCTGATTGTTCTCAACCAACCATAAAGATA

706_501Mod -CTCAGCCATTCTACCTATGTTCATTAACCGCTGATTGTTCTCAACCAACCATAAAGATA

707_501Mod -CTCAGCCATTCTACCTATGTTCATTAACCGCTGATTGTTCTCAACCAACCATAAAGATA

701_508Mod -CTCAGCCATTCTACCTATGTTCATTAACCGCTGATTGTTCTCAACCAACCATAAAGATA

712_501Mod -CTCAGCCATTCTACCTATGTTCATTAACCGCTGATTGTTCTCAACCAACCATAAAGATA

705_501Mod -CTCAGCCATTCTACCTATGTTCATTAACCGCTGATTGTTCTCAACCAACCATAAAGATA

711_501Mod -CTCAGCCATTCTACCTATGTTCATTAACCGCTGATTGTTCTCAACCAACCATAAAGATA

702_508Mod -CTCAGCCATTCTACCTATGTTCATTAACCGCTGATTGTTCTCAACCAACCATAAAGATA

Dama_mesopotamica -CTCAGCCATTTTACCTATGTTCATTAACCGCTGATTATTTTCAACTAACCATAAAGATA

Irish_Elk -CTCAGCCATTCTACCTATGTTCATTAACCGCTGATTATTTTCAACTAACCACAAAGATA

red_deer ACTCAGCCATTCTACCTATGTTCATTAACCGCTGATTATTTTCAACCAACCATAAAGATA

********** ************************* ** ***** ***** *******

709_501Mod TCGGTACCCTGTATCTACTATTTGGTGCCTGAGCAGGCATAGTAGGAACAGCTTTAAGCC

702_501Mod TCGGTACCCTGTATCTACTATTTGGTGCCTGAGCAGGCATAGTAGGAACAGCTTTAAGCC

Eemian TCGGTACCCTGTATCTACTATTTGGTGCCTGAGCAGGCATAGTAGGAACAGCTTTAAGCC

710_501Mod TCGGTACCCTGTATCTACTATTTGGTGCCTGAGCAGGCATAGTAGGAACAGCTTTAAGCC

706_501Mod TCGGTACCCTGTATCTACTATTTGGTGCCTGAGCAGGCATAGTAGGAACAGCTTTAAGCC

707_501Mod TCGGTACCCTGTATCTACTATTTGGTGCCTGAGCAGGCATAGTAGGAACAGCTTTAAGCC

701_508Mod TCGGTACCCTGTATCTACTATTTGGTGCCTGAGCAGGCATAGTAGGAACAGCTTTAAGCC

712_501Mod TCGGTACCCTGTATCTACTATTTGGTGCCTGAGCAGGCATAGTAGGAACAGCTTTAAGCC

705_501Mod TCGGTACCCTGTATCTACTATTTGGTGCCTGAGCAGGCATAGTAGGAACAGCTTTAAGCC

711_501Mod TCGGTACCCTGTATCTACTATTTGGTGCCTGAGCAGGCATAGTAGGAACAGCTTTAAGCC

702_508Mod TCGGTACCCTGTATCTACTATTTGGTGCCTGAGCAGGCATAGTAGGAACAGCTTTAAGCC

Dama_mesopotamica TCGGTACCCTGTATCTGCTATTTGGTGCCTGAGCAGGCATAGTAGGAACAGCCTTAAGCC

Irish_Elk TTGGTACTCTGTATCTACTATTTGGTGCTTGAGCAGGCATAGTAGGAACGGCCCTAAGCC

red_deer TCGGTACTCTGTATCTATTATTTGGTGCCTGAGCAGGCATAGTAGGGACAGCCTTAAGCC

* ***** ******** ********** ***************** ** ** ******

709_501Mod TATTGATTCGTGCTGAACTGGGTGGACCTAACTATCTTTTCTCTACACCTGGCAGGTGTC

702_501Mod TATTGATTCGTGCTGAACTGGGTGGACCTAACTATCTTTTCTCTACACCTGGCAGGTGTC

Eemian TATTGATTCGTGCTGAACTGG-TGGACCTAACTATCTTTTCTCTACACCTGGCAGGTGTC

710_501Mod TATTGATTCGTGCTGAACTGGGTGGACCTAACTATCTTTTCTCTACACCTGGCAGGTGTC

706_501Mod TATTGATTCGTGCTGAACTGGGTGGACCTAACTATCTTTTCTCTACACCTGGCGGGTGTC

707_501Mod TATTGATTCGTGCTGAACTGGGTGGACCTAACTATCTTTTCTCTACACCTGGCGGGTGTC

701_508Mod TATTGATTCGTGCTGAACTGGGTGGACCTAACTATCTTTTCTCTACACCTGGCAGGTGTC

712_501Mod TATTGATTCGTGCTGAACTGGGTGGACCTAACTATCTTTTCTCTACACCTGGCAGGTATC

705_501Mod TATTGATTCGTGCTGAACTGGGTGGACCTAACTATCTTTTCTCTACACCTGGCGGGTGTC

711_501Mod TATTGATTCGTGCTGAACTGGGTGGACCTAACTATCTTTTCTCTACACCTGGCAGGTATC

702_508Mod TATTGATTCGTGCTGAACTGGGTGGACCTAACTATCTTTTCTCTACACCTGGCAGGTGTC

Dama_mesopotamica TATTGATTCGTGCTGAACTGG--AGACCTGACTATTTTTTCTTTGCATCTGGCAGGTGTC

Irish_Elk TATTAACGCAGG--GGCCTCARTAGACYTGAYTATTTTTTCTTTACACCTGGCAGGTGTT

red_deer AGCTCATGCAGG--GGCTTCAGTAGACCTGACTATTTTTTCTTTACACTTAGCAGGCGTC

* * * * * * *** * * *** ****** * ** * ** ** *

709_501Mod TCTTCAATTCTAGGGGCCATTAACTTTATTACAACAATTATCAATATAAAACCCCCTGCT

702_501Mod TCTTCAATTCTAGGGGCCATTAACTTTATTACAACAATTATCAATATAAAACCCCCTGCT

Eemian TCTTCAATTCTAGGGGCCATTAACTTTATTACAACAATTATCAATATAAAACCCCCTGCT

710_501Mod TCTTCAATTCTAGGGGCCATTAACTTTATTACAACAATTATCAATATAAAACCCCCTGCT

706_501Mod TCTTCAATTCTAGGGGCCATTAACTTTATTACAACAATTATCAATATAAAACCCCCTGCT

707_501Mod TCTTCAATTCTAGGGGCCATTAACTTTATTACAACAATTATCAATATAAAACCCCCTGCT

701_508Mod TCTTCAATTCTAGGGGCCATTAACTTTATTACAACAATTATCAATATAAAACCCCCTGCT

712_501Mod TCTTCAATTCTAGGGGCCATTAACTTTATTACAACAATTATCAATATAAAACCCCCTGCT

705_501Mod TCTTCAATTCTAGGGGCCATTAACTTTATTACAACAATTATCAATATAAAACCCCCTGCT

711_501Mod TCTTCAATTCTAGGGGCCATTAACTTTATTACAACAATTATCAATATAAAACCCCCTGCT

702_508Mod TCTTCAATTCTAGGGGCCATTAACTTTATTACAACAATTATCAATATAAAACCCCCTGCT

Dama_mesopotamica TCTTCAATCCTAGGGGCCATTAACTTTATTACAACAATTATCAATATAAAACCTCCTGCC

Irish_Elk TCTTCAATCCTAGGGGCCATTAACTTTATTACAACAATTATCAATATAAAACCTCCTGCC

red_deer TCCTCAATTCTAGGGGCCATTAACTTTATTACAACAATTATCAATATAAAACCCCCTGCC

** ***** ******************************************** *****

709_501Mod ATGTCACAATACCAAACTCCCCTATTTGTGTGATCCGTACTAGTCACTGCTGTATTACTA

702_501Mod ATGTCACAATACCAAACTCCCCTATTTGTGTGATCCGTACTAGTCACTGCTGTATTACTA

Eemian ATGTCACAATACCAAACTCCCCTATTTGTGTGATCCGTACTAGTCACTGCTGTATTACTA

710_501Mod ATGTCACAATACCAAACTCCCCTATTTGTGTGATCCGTACTAGTCACTGCTGTATTACTA

706_501Mod ATGTCACAATACCAAACTCCCCTATTTGTGTGATCCGTACTAGTCACTGCTGTATTACTA

707_501Mod ATGTCACAATACCAAACTCCCCTATTTGTGTGATCCGTACTAGTCACTGCTGTATTACTA

701_508Mod ATGTCACAATACCAAACTCCCCTATTTGTGTGATCCGTACTAGTCACTGCTGTATTACTA

712_501Mod ATGTCACAATACCAAACTCCCCTATTTGTGTGATCCGTACTAGTCACTGCTGTATTACTA

705_501Mod ATGTCACAATACCAAACTCCCCTATTTGTGTGATCCGTACTAGTCACTGCTGTATTACTA

711_501Mod ATGTCACAATACCAAACTCCCCTATTTGTGTGATCCGTACTAGTCACTGCTGTATTACTA

702_508Mod ATGTCACAATACCAAACTCCCCTATTTGTGTGATCCGTACTAGTCACTGCTGTATTACTA

Dama_mesopotamica ATATCACAATATCAAACTCCCCTATTTGTGTGATCCGTACTAGTTACTGCTGTGTTATTA

Irish_Elk ATATCACAATATCAAACTCCCTTATTCGTGTGATCCGTACTAGTTACTGCTGTATTATTA

red_deer ATATCACAATATCAAACCCCTCTATTTGTGTGATCCGTATTAGTCACTGCTGTACTACTA

** ******** ***** ** **** ************ **** ******** ** **

709_501Mod CTTCTCTCACTCCCAGTACTAGCAGCTGGAATTACAATATTAT-TAACAGACCGAAATTT

702_501Mod CTTCTCTCACTCCCAGTACTAGCAGCTGGAATTACAATATTAT-TAACAGACCGAAATTT

Eemian CTTCTCTCACTCCCAGTACTAGCAGCCGGAATTACAATATTAT-TAACAGACCGAAATT-

710_501Mod CTTCTCTCACTCCCAGTACTAGCAGCTGGAATTACAATATTAT-TAACAGACCGAAATTT

706_501Mod CTTCTCTCACTCCCAGTACTAGCAGCCGGAATTACAATATTAT-TAACAGACCGAAATTT

707_501Mod CTTCTCTCACTCCCAGTACTAGCAGCCGGAATTACAATATTAT-TAACAGACCGAAATTT

701_508Mod CTTCTCTCACTCCCAGTACTAGCAGCCGGAATTACAATATTAT-TAACAGACCGAAATTT

712_501Mod CTTCTCTCACTCCCAGTACTAGCAGCCGGAATTACAATATTAT-TAACAGACCGAAATTT

705_501Mod CTTCTCTCACTCCCAGTACTAGCAGCCGGAATTACAATATTAT-TAACAGACCGAAATTT

711_501Mod CTTCTCTCACTCCCAGTACTAGCAGCCGGAATTACAATATTAT-TAACAGACCGAAATTT

702_508Mod CTTCTCTCACTCCCAGTACTAGCAGCCGGAATTACAATATTAT-TAACAGACCGAAATTT

Dama_mesopotamica CTTCTCTCACTCCCTGTACTAGCAGCCGGAATTACAATATTAT-TAACAGACCGAAATT-

Irish_Elk CTTCTCTCACTCCCTGTACTAGCAGCCGGAATTACAATATTATGCAGGAGGTGGAGAT--

red_deer CTTCTCTCACTCCCTGTACTAGCAGCCGGAATTACAAGACCCAGCAGGAGGCGGAGAT--

************** *********** ********** * * ** ** **

709_501Mod CCCATTCTATATCAACACTTATTCTGATTCTTTGGTCACCCTGAAGTATATATTCTTATT

702_501Mod CCCATTCTATATCAACACTTATTCTGATTCTTTGGTCACCCTGAAGTATATATTCTTATT

Eemian CCCATTCTATATCAACACTTATTCTGATTCTTTGGTCACCCTGAAGTATATATTCTTATT

710_501Mod CCCATTCTATATCAACACTTATTCTGATTCTTTGGTCACCCTGAAGTATATATTCTTATT

706_501Mod CCCATTCTATATCAACACTTATTCTGATTCTTTGGTCACCCTGAAGTATATATTCTTATT

707_501Mod CCCATTCTATATCAACACTTATTCTGATTCTTTGGTCACCCTGAAGTATATATTCTTATT

701_508Mod CCCATTCTATATCAACACTTATTCTGATTCTTTGGTCACCCTGAAGTATATATTCTTATT

712_501Mod CCCATTCTATATCAACACTTATTCTGATTCTTTGGTCACCCTGAAGTATATATTCTTATT

705_501Mod CCCATTCTATATCAACACTTATTCTGATTCTTTGGTCACCCTGAAGTATATATTCTTATT

711_501Mod CCCATTCTATATCAACACTTATTCTGATTCTTTGGTCACCCTGAAGTATATATTCTTATT

702_508Mod CCCATTCTATATCAACACTTATTCTGATTCTTTGGTCACCCTGAAGTATATATTCTTATT

Dama_mesopotamica CCCATTCTATATCAACACTTATTCTGATTCTTTGGCCACCCTGAAGTATACATTCTTATT

Irish_Elk CCCATTCTATATCAACACTTATTCTGATTCTTTGGCCACCCTGAAGTGTATATTCTCATT

red_deer CCTATTCTATATCAACACTTATTCTGATTCTTTGGCCACCCTGAAGTATATATCCTTATT

** ******************************** *********** ** ** ** ***

709_501Mod TTACCCGGCTTTGGCATAATTTCCCATATCGTAACATATTATTCAGGAAAAAAAGAACCA

702_501Mod TTACCCGGCTTTGGCATAATTTCCCATATCGTAACATATTATTCAGGAAAAAAAGAACCA

Eemian TTACCCGGCTTTGGCATAATTTCCCATATCGAAA----TTAT-CAGGAAAAAAAGAACCA

710_501Mod TTACCCGGCTTTGGCATAATTTCCCATATCGTAACATATTATTCAGGAAAAAAAGAACCA

706_501Mod TTACCCGGCTTTGGCATAATTTCCCATATCGTAACATATTATTCAGGAAAAAAAGAACCA

707_501Mod TTACCCGGCTTTGGCATAATTTCCCATATCGTAACATATTATTCAGGAAAAAAAGAACCA

701_508Mod TTACCCGGCTTTGGCATAATTTCCCATATCGTAACATATTATTCAGGAAAAAAAGAACCA

712_501Mod TTACCCGGCTTTGGCATAATTTCCCATATCGTAACATATTATTCAGGAAAAAAAGAACCA

705_501Mod TTACCCGGCTTTGGCATAATTTCCCATATCGTAACATATTATTCAGGAAAAAAAGAACCA

711_501Mod TTACCCGGCTTTGGCATAATTTCCCATATCGTAACATATTATTCAGGAAAAAAAGAACCA

702_508Mod TTACCCGGCTTTGGCATAATTTCCCATATCGTAACATATTATTCAGGAAAAAAAGAACCA

Dama_mesopotamica CTACCCGGCTTTGGTATAATTTCACATATCGTAACATATTACTCAGGAAAAAAAGAACCA

Irish_Elk CTACCCGGCTTTGGTATGATCTCCCATATCGTAACATATTACTCAGGAAAAAAAGAACCA

red_deer CTACCCGGCTTTGGTATAATCTCCCACATCGTAACATACTATTCAGGAAAAAAAGAACCA

************* ** ** ** ** **** ** ** *****************

709_501Mod TTTGGGTACATAGGAATGGTCTGGGCTATAATATCAATTGGATTTTTAGTACA--CGAGC

702_501Mod TTTGGGTACATAGGAATGGTCTGGGCTATAATATCAATTGGATTTTTAGTACA--CGAGC

Eemian TTTGGGTACATAGGAATGGTCTGGGCTATAATATCAATTGGATTTTTATTCCGATCTAGC

710_501Mod TTTGGGTACATAGGAATGGTCTGGGCTATAATATCAATTGGATTTTTAGTACA--CGAGC

706_501Mod TTTGGGTACATAGGAATGGTCTGGGCTATAATATCAATTGGATTTTTAGTACA--CGAGC

707_501Mod TTTGGGTACATAGGAATGGTCTGGGCTATAATATCAATTGGATTTTTAGTACA--CGAGC

701_508Mod TTTGGGTACATAGGAATGGTCTGGGCTATAATATCAATTGGATTTTTAGTACA--CGAGC

712_501Mod TTTGGGTACATAGGAATGGTCTGGGCTATAATATCAATTGGATTTTTAGTACA--CGAGC

705_501Mod TTTGGGTACATAGGAATGGTCTGGGCTATAATATCAATTGGATTTTTAGTACA--CGAGC

711_501Mod TTTGGGTACATAGGAATGGTCTGGGCTATAATATCAATTGGATTTTTAGTACA--CGAGC

702_508Mod TTTGGGTACATAGGAATGGTCTGGGCTATAATATCAATTGGATTTTTAGTACA--CGAGC

Dama_mesopotamica TTTGGGTACATAGGAATGGTCTGGGCTATAATATCAATTGGATTCTTAG-ACA--CGAGC

Irish_Elk TTTGGGTACATAGGAATGGTCTGGGCCATAATATGGTATAGATGTCGAT-ACA--CGAGC

red_deer TTTGGGTACATAGGAATAGTCTGGGCTAACAGTCGGAATAGATGTTGAC-ACG--CGAGC

***************** ******** * * * *** * * * ***

709_501Mod CTACTTTACATCAGCTACCATAATTATTGCTATTCCGACTGGGGTAAAAGTCTTCAGTTG

702_501Mod CTACTTTACATCAGCTACCATAATTATTGCTATTCCGACTGGGGTAAAAGTCTTCAGTTG

Eemian CTACTTTACATCAGCTACCATAATTATTGCTATTACGACTGGGGTAAAAGTCTTCAGTTG

710_501Mod CTACTTTACATCAGCTACCATAATTATTGCTATTCCGACTGGGGTAAAAGTCTTCAGTTG

706_501Mod CTACTTTACATCAGCTACCATAATTATTGCTATTCCGACTGGGGTAAAAGTCTTCAGTTG

707_501Mod CTACTTTACATCAGCTACCATAATTATTGCTATTCCGACTGGGGTAAAAGTCTTCAGTTG

701_508Mod CTACTTTACATCAGCTACCATAATTATTGCTATTCCGACTGGGGTAAAAGTCTTCAGTTG

712_501Mod CTACTTTACATCAGCTACCATAATTATTGCTATTCCGACTGGGGTAAAAGTCTTCAGTTG

705_501Mod CTACTTTACATCAGCTACCATAATTATTGCTATTCCGACTGGGGTAAAAGTCTTCAGTTG

711_501Mod CTACTTTACATCAGCTACCATAATTATTGCTATTCCGACTGGGGTAAAAGTCTTCAGTTG

702_508Mod CTACTTTACATCAGCTACCATAATTATTGCTATTCCGACTGGGGTAAAAGTCTTCAGTTG

Dama_mesopotamica CTATTTTACATCAGCTACCATAATTATTGCTATCCCAACTGGAGTAAAAGTCTTTAGTTG

Irish_Elk CTATTTCACATCAGCTACTATAATTATTGCCATCCCAACTGGAGTAAAAGTCTTCAGTTG

red_deer CTATTTCACATCAGCTACTATAATTATTGCCATCCCAACGGGAGTAAAAGTCTTTAGTTG

*** ** *********** *********** ** * ** ** *********** *****

709_501Mod ATTAGCAACACTCCACGGAGGTAATATTAAATGATCACCTGCTATAATATGAG-------

702_501Mod ATTAGCAACACTCCACGGAGGTAATATTAAATGATCACCTGCTATAATATGAG-------

Eemian ATTAGCAACACTCCACGGAGGTAATATTAAATGATCACCTGCTATAATA-GAG-------

710_501Mod ATTAGCAACACTCCACGGAGGTAATATTAAATGATCACCTGCTATAATATGAG-------

706_501Mod ATTAGCAACACTCCACGGAGGTAATATTAAATGATCACCTGCTATAATATGAG-------

707_501Mod ATTAGCAACACTCCACGGAGGTAATATTAAATGATCACCTGCTATAATATGAG-------

701_508Mod ATTAGCAACACTCCACGGAGGTAATATTAAATGATCACCTGCTATAATATGAG-------

712_501Mod ATTAGCAACACTCCACGGAGGTAATATTAAATGATCACCTGCTATAATATGAG-------

705_501Mod ATTAGCAACACTCCACGGAGGTAATATTAAATGATCACCTGCTATAATATGAG-------

711_501Mod ATTAGCAACACTCCACGGAGGTAATATTAAATGATCACCTGCTATAATATGAG-------

702_508Mod ATTAGCAACACTCCACGGAGGTAATATTAAATGATCACCTGCTATAATATGAG-------

Dama_mesopotamica ATTAGCAACACTCCACGGAGGTAATATTAAATGATCGCCTGCTATAATATGAG-------

Irish_Elk ATTAGCAACACTCCACGGAGGCAATATTAAATGATCACCTGCTATAATATGAGCTTTAGA

red_deer ATTAGCAACACTCCACGGAGGTAATATTAAATGATCACCTGCTTTAGAGTGA--------

********************* ************** ****** ** **

709_501Mod CTTTA-GGTTT--CCCCACCATACCACACATTTGAAGAACCTACATACGTTAACTTGAAA

702_501Mod CTTTA-GGTTT--CCCCACCATACCACACATTTGAAGAACCTACATACGTTAACTTGAAA

Eemian C-------------CCCACCATATCATACATTTGAAGAACCTACATACATTAACTTAAAA

710_501Mod CTTTA-GGTTT--CCCCACCATACCACACATTTGAAGAACCTACATACGTTAACTTGAAA

706_501Mod CTTTA-GGTTT--CCCCGCCATACCACACATTTGAAGAACCTACATACGTTAACTTGAAA

707_501Mod CTTTA-GGTTT--CCCCGCCATACCACACATTTGAAGAACCTACATACGTTAACTTGAAA

701_508Mod CTTTA-GGTTT--CCCCGCCATACCACACATTTGAAGAACCTACATACGTTAACTTGAAA

712_501Mod CTTTA-GGTTT--CCCCGCCATACCACACATTTGAAGAACCTACATACGTTAACTTGAAA

705_501Mod CTTTA-GGTTT--CCCCGCCATACCACACATTTGAAGAACCTACATACGTTAACTTGAAA

711_501Mod CTTTA-GGTTT--CCCCGCCATACCACACATTTGAAGAACCTACATACGTTAACTTGAAA

702_508Mod CTTTA-GGTTT--CCCCGCCATACCACACATTTGAAGAACCTACATACGTTAACTTGAAA

Dama_mesopotamica CTTTA-GGCTTTCCCCCACCATATCATACATTTGAAGAACCTACATACATTAACTTAAAA

Irish_Elk CTAAATGGATGCCCCCCACCATACCACACATTTGAAGAACCTACATACGTTAACTTAA--

red_deer CTAAATGGATGTCCCCCACCATATCATACATTTGAAGAACCTACATACGTTAACTTAAAA

* *** ***** ** ********************* ******* *

709_501Mod TAAGAAAGGAAGGAATCGAACCCCCTATAGCTGGTTTCAAGCCAACATCATAACCACTAT

702_501Mod TAAGAAAGGAAGGAATCGAACCCCCTATAGCTGGTTTCAAGCCAACATCATAACCACTAT

Eemian TAAGAAAGGAAGGAATCGAACCCCCCATAGCTGGTTTCAAGCCAACATCATAACCACTAT

710_501Mod TAAGAAAGGAAGGAATCGAACCCCCTATAGCTGGTTTCAAGCCAACATCATAACCACTAT

706_501Mod TAAGAAAGGAAGGAATCGAACCCCCTATAGCTGGTTTCAAGCCAACATCATAACCACTAT

707_501Mod TAAGAAAGGAAGGAATCGAACCCCCTATAGCTGGTTTCAAGCCAACATCATAACCACTAT

701_508Mod TAAGAAAGGAAGGAATCGAACCCCCTATAGCTGGTTTCAAGCCAACATCATAACCACTAT

712_501Mod TAAGAAAGGAAGGAATCGAACCCCCTATAGCTGGTTTCAAGCCAACATCATAACCACTAT

705_501Mod TAAGAAAGGAAGGAATCGAACCCCCTATAGCTGGTTTCAAGCCAACATCATAACCACTAT

711_501Mod TAAGAAAGGAAGGAATCGAACCCCCTATAGCTGGTTTCAAGCCAACATCATAACCACTAT

702_508Mod TAAGAAAGGAAGGAATCGAACCCCCTATAGCTGGTTTCAAGCCAACATCATAACCACTAT

Dama_mesopotamica TAAGAAAGGAAGGAATCGAACCCCCCATAGCTGGTTTCAAGCCAACATCATAACCACTAT

Irish_Elk TAAGAAAGGAAGGAATCGAACCCCCCATAGCTGGTTTCAAGCCAACATCATAACCACTAT

red_deer TAAGAAAGGAAGGAATCGAACCCCCCATAGCTGGTTTCAAGCCAACATCATAACCACTAT

************************* **********************************

709_501Mod GTCTTTCTCAATTAATGAGGTGTTAGTAAAACATTATATAACTTTGTCAGGGTTAAGTTA

702_501Mod GTCTTTCTCAATTAATGAGGTGTTAGTAAAACATTATATAACTTTGTCAGGGTTAAGTTA

Eemian GTCTTTCTCAATTAATGAGGTGTTAGTAAAATATTATATAACTTTGTCAAGGTTAAGTTA

710_501Mod GTCTTTCTCAATTAATGAGGTGTTAGTAAAACATTATATAACTTTGTCAGGGTTAAGTTA

706_501Mod GTCTTTCTCAATTAATGAGGTGTTAGTAAAACATTATATAACTTTGTCAGGGTTAAGTTA

707_501Mod GTCTTTCTCAATTAATGAGGTGTTAGTAAAACATTATATAACTTTGTCAGGGTTAAGTTA

701_508Mod GTCTTTCTCAATTAATGAGGTGTTAGTAAAACATTATATAACTTTGTCAGGGTTAAGTTA

712_501Mod GTCTTTCTCAATTAATGAGGTGTTAGTAAAACATTATATAACTTTGTCAGGGTTAAGTTA

705_501Mod GTCTTTCTCAATTAATGAGGTGTTAGTAAAACATTATATAACTTTGTCAGGGTTAAGTTA

711_501Mod GTCTTTCTCAATTAATGAGGTGTTAGTAAAACATTATATAACTTTGTCAGGGTTAAGTTA

702_508Mod GTCTTTCTCAATTAATGAGGTGTTAGTAAAACATTATATAACTTTGTCAGGGTTAAGTTA

Dama_mesopotamica GTCTTTCTCAATTAATGAGGTGTTAGTAAAATATTATATAACTTTGTCAAGGTTAAGTTA

Irish_Elk GTCTTTCTCAATCAATGAGGTGTTAGTAAAATATTATATAACTTTGTCAGGGTATTATAG

red_deer GTCTTTCTCAATTAATGAGGTGTTAGTAAAATATTATATAACTTACATCACCTATTATAG

************ ****************** ************ * *

709_501Mod CAGGTAATTATTACATTTTCATGATCACACGCTAATAATTGTTTTTCTAATCAGCTCGCT

702_501Mod CAGGTAATTATTACATTTTCATGATCACACGCTAATAATTGTTTTTCTAATCAGCTCGCT

Eemian CAGGT-----------TTCCATGATCACACACTAATAATTGTTTTTCTAATTAGCTCGCT

710_501Mod CAGGTAATTATTACATTTTCATGATCACACGCTAATAATTGTTTTTCTAATCAGCTCGCT

706_501Mod CAGGTAATTATTACATTTTCATGATCACACGCTAATAATTGTTTTTCTAATCAGCTCGCT

707_501Mod CAGGTAATTATTACATTTTCATGATCACACGCTAATAATTGTTTTTCTAATCAGCTCGCT

701_508Mod CAGGTAATTATTACATTTTCATGATCACACGCTAATAATTGTTTTTCTAATCAGCTCGCT

712_501Mod CAGGTAATTATTACATTTTCATGATCACACGCTAATAATTGTTTTTCTAATCAGCTCGCT

705_501Mod CAGGTAATTATTACATTTTCATGATCACACGCTAATAATTGTTTTTCTAATCAGCTCGCT

711_501Mod CAGGTAATTATTACATTTTCATGATCACACGCTAATAATTGTTTTTCTAATCAGCTCGCT

702_508Mod CAGGTAATTATTACATTTTCATGATCACACGCTAATAATTGTTTTTCTAATCAGCTCGCT

Dama_mesopotamica CAGGT--CTACTACATTTCCATGATCACACACTAATAATTGTTTTTCTAATCAGCTCGCT

Irish_Elk AAGAA--TTACTACATTTCCATGATCACACACTAATAATTGTTTTTCTAATCAGCTCACT

red_deer AAGAA--TTACTACATTTTCATGATCATACATTAATAATCGTTTTTCTAATCAGCTCACT

** ** ******** ** ******* *********** ***** **

709_501Mod AGTACTCTATGTTATCTCATTAATACTAACAACAAAATTAACACACACTAGCACAATAGA

702_501Mod AGTACTCTATGTTATCTCATTAATACTAACAACAAAATTAACACACACTAGCACAATAGA

Eemian AGTACTCTATGTCATCTCATTAATATTAACAACAAAATTAACACACACTAGCACAATAGA

710_501Mod AGTACTCTATGTTATCTCATTAATACTAACAACAAAATTAACACACACTAGCACAATAGA

706_501Mod AGTACTCTATGTTATCTCATTAATACTAACAACAAAATTAACACACACTAGCACAATAGA

707_501Mod AGTACTCTATGTTATCTCATTAATACTAACAACAAAATTAACACACACTAGCACAATAGA

701_508Mod AGTACTCTATGTTATCTCATTAATACTAACAACAAAATTAACACACACTAGCACAATAGA

712_501Mod AGTACTCTATGTTATCTCATTAATACTAACAACAAAATTAACACACACTAGCACAATAGA

705_501Mod AGTACTCTATGTTATCTCATTAATACTAACAACAAAATTAACACACACTAGCACAATAGA

711_501Mod AGTACTCTATGTTATCTCATTAATACTAACAACAAAATTAACACACACTAGCACAATAGA

702_508Mod AGTACTCTATGTTATCTCATTAATACTAACAACAAAATTAACACACACTAGCACAATAGA

Dama_mesopotamica AGTACTCTATGTCATCTCATTAATATTAACAACAAAATTAACACACACTAGCACAATAGA

Irish_Elk AGTACTCTACGTCATCTCATTAATGCTAACGACAAAATTAACACACACTAGCAC------

red_deer AGTACTCTACGTCATTTCATTAATGCTAACGACAAAATTAACACACACTAGTACCGT--A

********* ** ** ******** **** ******************** **

709_501Mod TGCCCAAGAGGTAAATGATCTGCATCAATATTATAAAATCATTAAGAAGCTAAAATAGCA

702_501Mod TGCCCAAGAGGTAAATGATCTGCATCAATATTATAAAATCATTAAGAAGCTAAAATAGCA

Eemian TGCCCAAGAAA-AAATGATCTGCATCAATATTATAAAATCATTAAGAAGCTAAAATAGCA

710_501Mod TGCCCAAGAGGTAAATGATCTGCATCAATATTATAAAATCATTAAGAAGCTAAAATAGCA

706_501Mod TGCCCAAGAGGTAAATGATCTGCATCAATATTATAAAATCATTAAGAAGCTAAAATAGCA

707_501Mod TGCCCAAGAGGTAAATGATCTGCATCAATATTATAAAATCATTAAGAAGCTAAAATAGCA

701_508Mod TGCCCAAGAGGTAAATGATCTGCATCAATATTATAAAATCATTAAGAAGCTAAAATAGCA

712_501Mod TGCCCAAGAGGTAAATGATCTGCATCAATATTATAAAATCATTAAGAAGCTAAAATAGCA

705_501Mod TGCCCAAGAGGTAAATGATCTGCATCAATATTATAAAATCATTAAGAAGCTAAAATAGCA

711_501Mod TGCCCAAGAGGTAAATGATCTGCATCAATATTATAAAATCATTAAGAAGCTAAAATAGCA

702_508Mod TGCCCAAGAGGTAAATGATCTGCATCAATATTATAAAATCATTAAGAAGCTAAAATAGCA

Dama_mesopotamica TGCCCAAGAAGTAG----------------------------------------------

Irish_Elk ------------------------------------------------------------

red_deer CGACTAACAGCTAA----------------------------------------------

709_501Mod ATAGCCTTTTAAGCTAGAGATTATTACTGCAGGGCACCTGCTAATTCACTTAATTGGAGG

702_501Mod ATAGCCTTTTAAGCTAGAGATTATTACTGCAGGGCACCTGCTAATTCACTTAATTGGAGG

Eemian ATAGCCTTTTAAGCTAGAGAT-ATTACTGCAGGGCACCTGCTAATTCACTTAATTGGAGG

710_501Mod ATAGCCTTTTAAGCTAGAGATTATTACTGCAGGGCACCTGCTAATTCACTTAATTGGAGG

706_501Mod ATAGCCTTTTAAGCTAGAGATTATTACTGCAGGGCACCTGCTAATTCACTTAATTGGAGG

707_501Mod ATAGCCTTTTAAGCTAGAGATTATTACTGCAGGGCACCTGCTAATTCACTTAATTGGAGG

701_508Mod ATAGCCTTTTAAGCTAGAGATTATTACTGCAGGGCACCTGCTAATTCACTTAATTGGAGG

712_501Mod ATAGCCTTTTAAGCTAGAGATTATTACTGCAGGGCACCTGCTAATTCACTTAATTGGAGG

705_501Mod ATAGCCTTTTAAGCTAGAGATTATTACTGCAGGGCACCTGCTAATTCACTTAATTGGAGG

711_501Mod ATAGCCTTTTAAGCTAGAGATTATTACTGCAGGGCACCTGCTAATTCACTTAATTGGAGG

702_508Mod ATAGCCTTTTAAGCTAGAGATTATTACTGCAGGGCACCTGCTAATTCACTTAATTGGAGG

Dama_mesopotamica -----------------------TTACTGCAGGACACCTGCTAATTCACTTAATTGGAGG

Irish_Elk ------------------------------------------------------------

red_deer ---------------------CATTACTGCAGGACACCTACTAATTCACCTAATTGGAGG

709_501Mod GGCTACACTTGCACTAATAAGCATCAGTACTACAATAGCTCTTATTACATTTACTGTTCT

702_501Mod GGCTACACTTGCACTAATAAGCATCAGTACTACAATAGCTCTTATTACATTTACTGTTCT

Eemian GGCTACACTTGCACTAATAAGCATCAGTACTACAATAGCTCTTATTACATTTACTGTTCT

710_501Mod GGCTACACTTGCACTAATAAGCATCAGTACTACAATAGCTCTTATTACATTTACTGTTCT

706_501Mod GGCTACACTTGCACTAATAAGCATCAGTACTACAATAGCTCTTATTACATTTACTGTTCT

707_501Mod GGCTACACTTGCACTAATAAGCATCAGTACTACAATAGCTCTTATTACATTTACTGTTCT

701_508Mod GGCTACACTTGCACTAATAAGCATCAGTACTACAATAGCTCTTATTACATTTACTGTTCT

712_501Mod GGCTACACTTGCACTAATAAGCATCAGTACTACAATAGCTCTTATTACATTTACTGTTCT

705_501Mod GGCTACACTTGCACTAATAAGCATCAGTACTACAATAGCTCTTATTACATTTACTGTTCT

711_501Mod GGCTACACTTGCACTAATAAGCATCAGTACTACAATAGCTCTTATTACATTTACTGTTCT

702_508Mod GGCTACACTTGCACTAATAAGCATCAGTACTACAATAGCTCTTATTACATTTACTGTTCT

Dama_mesopotamica AGCCACACTTGCACTAACAAGCATCAGTACTACAATAGCTCTTATTACATTTATTATTCT

Irish_Elk ---------------AATAGAT--------------------------------------

red_deer AGCTGCACTTGCACTAATAAGTATCAGTACTACAATAGCTCTCATCACATTTATTATTCT

** *

709_501Mod GGTCTTACTTACAATCCTTGAGTTTGCAGTAGCCATAATCCAAGCCTACGACTTCTGGCT

702_501Mod GGTCTTACTTACAATCCTTGAGTTTGCAGTAGCCATAATCCAAGCCTACGACTTCTGGCT

Eemian GGTCTTACTTACAATCCTTGAGTTTGCAGTAGCCATAATCCAAGCCTAC--CTTCTGACC

710_501Mod GGTCTTACTTACAATCCTTGAGTTTGCAGTAGCCATAATCCAAGCCTACGACTTCTGGCT

706_501Mod GGTCTTACTTACAATCCTTGAGTTTGCAGTAGCCATAATCCAAGCCTACGACTTCTGGCT

707_501Mod GGTCTTACTTACAATCCTTGAGTTTGCAGTAGCCATAATCCAAGCCTACGACTTCTGGCT

701_508Mod GGTCTTACTTACAATCCTTGAGTTTGCAGTAGCCATAATCCAAGCCTACGACTTCTGGCT

712_501Mod GGTCTTACTTACAATCCTTGAGTTTGCAGTAGCCATAATCCAAGCCTACGACTTCTGGCT

705_501Mod GGTCTTACTTACAATCCTTGAGTTTGCAGTAGCCATAATCCAAGCCTACGACTTCTGGCT

711_501Mod GGTCTTACTTACAATCCTTGAGTTTGCAGTAGCCATAATCCAAGCCTACGACTTCTGGCT

702_508Mod GGTCTTACTTACAATCCTTGAGTTTGCAGTAGCCATAATCCAAGCCTACGACTTCTGGCT

Dama_mesopotamica AGTTCTACTCACAATCCTTGAATTCGCAGTAGCTATAATCCAAGCCTACG-CTTCTGGCT

Irish_Elk --------------------------------------GCCCTACTAATAACTTCTGGCT

red_deer AGTCCTACTCACAATCCTTGAGTTTGCAGCT-CTATCAGCCCTATTAATAACTTCCGGCT

** * **** * *

709_501Mod TAATTATATGATTTCATTTCAACTCAATAGTCCTACTGACACTTGGCCTAACAACAAACA

702_501Mod TAATTATATGATTTCATTTCAACTCAATAGTCCTACTGACACTTGGCCTAACAACAAACA

Eemian T-------TGATTTCATTTCAACTCAATAGTCCTACTGACACTTGGCCTAACAACAAACA

710_501Mod TAATTATATGATTTCATTTCAACTCAATAGTCCTACTGACACTTGGCCTAACAACAAACA

706_501Mod TAATTATATGATTTCATTTCAACTCAATAGTCCTACTGACACTTGGCCTAACAACAAACA

707_501Mod TAATTATATGATTTCATTTCAACTCAATAGTCCTACTGACACTTGGCCTAACAACAAACA

701_508Mod TAATTATATGATTTCATTTCAACTCAATAGTCCTACTGACACTTGGCCTAACAACAAATA

712_501Mod TAATTATATGATTTCATTTCAACTCAATAGTCCTACTGACACTTGGCCTAACAACAAACA

705_501Mod TAATTATATGATTTCATTTCAACTCAATAGTCCTACTGACACTTGGCCTAACAACAAACA

711_501Mod TAATTATATGATTTCATTTCAACTCAATAGTCCTACTGACACTTGGCCTAACAACAAACA

702_508Mod TAATTATATGATTTCATTTCAACTCAATAGTCCTACTGACACTTGGCCTAACAACAAACA

Dama_mesopotamica TAATTATATGATTTCATTTCAACTCAATAATCCTATTAACACTTGGCTTAACAACAAACA

Irish_Elk TAATTATATGATTTCATTTCAACTCANNNNNNNNNTTAACATTTGGCCTGACAACAAATA

red_deer TAATTATATGATTTCATTTTAACTCAATAGTCCTATTAACACTTGGCCTAACAACAAATA

* *********** ****** * *** ***** * ******** *

709_501Mod TACTTACAATATATCAATGATGACGAGATATCATTCGAGAAAGTTATGGCTCAACCTTCT

702_501Mod TACTTACAATATATCAATGATGACGAGATATCATTCGAGAAAGTTATGGCTCAACCTTCT

Eemian TACTTACAATATATCAATGATGACGAGATATCATTCGAGAAAGTT-------------CT

710_501Mod TACTTACAATATATCAATGATGACGAGATATCATTCGAGAAAGTTATGGCTCAACCTTCT

706_501Mod TACTTACAATATATCAATGATGACGAGATATCATTCGAGAAAGTTATGGCTCAACCTTCT

707_501Mod TACTTACAATATATCAATGATGACGAGATATCATTCGAGAAAGTTATGGCTCAACCTTCT

701_508Mod TACTTACAATATATCAATGATGACGAGATATCATTCGAGAAAGTTATGGCTCAACCTTCT

712_501Mod TACTTACAATATATCAATGATGACGAGATATCATTCGAGAAAGTTATGGCTCAACCTTCT

705_501Mod TACTTACAATATATCAATGATGACGAGATATCATTCGAGAAAGTTATGGCTCAACCTTCT

711_501Mod TACTTACAATATATCAATGATGACGAGATATCATTCGAGAAAGTTATGGCTCAACCTTCT

702_508Mod TACTTACAATATATCAATGATGACGAGATATCATTCGAGAAAGTTATGGCTCAACCTTCT

Dama_mesopotamica TACTTACAATATATCAATGATGACGAGACATTATTCGAGAAAGC-ATGGTTCAACTTTCT

Irish_Elk TACTTACAATATATCAATGATGACGAGATATT-CAGACGGAGTTTATGGCTCAACCTTCT

red_deer TACTTACAATATACCAATGATGGTTACCATTT-CAGACGGAGTTTATGGCTCAACCTTCT

************* ******** * * * * **

709_501Mod TCGTAGCTACAGGCTTCCACGGCCTACATGTTATCATCGGATCTACTTTCTTAATTGTCT

702_501Mod TCGTAGCTACAGGCTTCCACGGCCTACATGTTATCATCGGATCTACTTTCTTAATTGTCT

Eemian TCGTAGCTACAGGCTTCCACGGCCTACATGTTATCATCGGATCTACTTTCTTAATTGTCT

710_501Mod TCGTAGCTACAGGCTTCCACGGCCTACATGTTATCATCGGATCTACTTTCTTAATTGTCT

706_501Mod TCGTAGCTACAGGCTTCCACGGCCTACATGTTATCATCGGATCTACTTTCTTAATTGTCT

707_501Mod TCGTAGCTACAGGCTTCCACGGCCTACATGTTATCATCGGATCTACTTTCTTAATTGTCT

701_508Mod TCGTAGCTACAGGCTTCCACGGCCTACATGTTATCATCGGATCTACTTTCTTAATTGTCT

712_501Mod TCGTAGCTACAGGCTTCCACGGCCTACATGTTATCATCGGATCTACTTTCTTAATTGTCT

705_501Mod TCGTAGCTACAGGCTTCCACGGCCTACATGTTATCATCGGATCTACTTTCTTAATTGTCT

711_501Mod TCGTAGCTACAGGCTTCCACGGCCTACATGTTATCATCGGATCTACTTTCTTAATTGTCT

702_508Mod TCGTAGCTACAGGCTTCCACGGCCTACATGTTATCATCGGATCTACTTTCTTAATTGTCT

Dama_mesopotamica TCGTAGCTACAGGTTTCCATGGCCTACACGTCATCATTGGGTCTACTTTCTTAATTGTCT

Irish_Elk TCGTAGCCACAGGCTTCCACGGCCTACATGTTATCATTGGGTCTACTTTCTTAATTGTCT

red_deer TTGTAGCTACAGGCTTCCATGGCCTACATGTCATTATTGGATCTACCTTCTTAATTGTCT

* ***** ***** ***** ******** ** ** ** ** ***** *************

709_501Mod GCTTCTTTCGCCAATTAAAATTCCACTTTACTTCCAGCCAT---CACTAGTAGGCTCTCT

702_501Mod GCTTCTTTCGCCAATTAAAATTCCACTTTACTTCCAGCCAT---CACTAGTAGGTTCTCT

Eemian GCTTCTTTCGCCAATTAAAATTCCACTTTACTTCCAAC-------GCTAGTAGGTTCTCT

710_501Mod GCTTCTTTCGCCAATTAAAATTCCACTTTACTTCCAGCCAT---CACTAGTAGGCTCTCT

706_501Mod GCTTCTTTCGCCAATTAAAATTCCACTTTACTTCCAGCCAT---CACTAGTAGGTTCTCT

707_501Mod GCTTCTTTCGCCAATTAAAATTCCACTTTACTTCCAGCCAT---CACTAGTAGGTTCTCT

701_508Mod GCTTCTTTCGCCAATTAAAATTCCACTTTACTTCCAGCCAT---CACTAGTAGGTTCTCT

712_501Mod GCTTCTTTCGCCAATTAAAATTCCACTTTACTTCCAGCCAT---CACTAGTAGGTTCTCT

705_501Mod GCTTCTTTCGCCAATTAAAATTCCACTTTACTTCCAGCCAT---CACTAGTAGGTTCTCT

711_501Mod GCTTCTTTCGCCAATTAAAATTCCACTTTACTTCCAGCCAT---CACTAGTAGGTTCTCT

702_508Mod GCTTCTTTCGCCAATTAAAATTCCACTTTACTTCCAGCCAT---CACTAGTAGGTTCTCT

Dama_mesopotamica GCTTTTTTCGTCAATTAAAATTCCACTTTACTTCCAGCCAC---CACTAGTAGGTTCTCT

Irish_Elk GCTTTTTTCGCCAATTAAAATTCCACTTCACT----ATTTTATACACTAGTAGGTTCTCT

red_deer GCTTTTTTCGCCAATTAAAATTTCACTCTACTTCCTGTTTTATACACTAGTAGGCTCTCT

**** ***** *********** **** *** ******** *****

709_501Mod CCCCCTACTAGTCGCATTGGTTTATCTCCAAAACATTACTGGGTCTCTAAACTTTTTAGT

702_501Mod CCCCCTACTAGTCGCATTGGTTTATCTCCAAAACATTACTGGGTCTCTAAACTTTTTAGT

Eemian TCCACTACTAGTTGCACTAGTCTACCTCCAAAACATTACTGGATCTCTAAACTTTCTAGT

710_501Mod CCCCCTACTAGTCGCATTGGTTTATCTCCAAAACATTACTGGGTCTCTAAACTTTTTAGT

706_501Mod CCCCCTACTAGTCGCATTGGTTTATCTCCAAAACATTACTGGGTCTCTAAACTTTTTAGT

707_501Mod CCCCCTACTAGTCGCATTGGTTTATCTCCAAAACATTACTGGGTCTCTAAACTTTTTAGT

701_508Mod CCCCCTACTAGTCGCATTGGTTTATCTCCAAAACATTACTGGGTCTCTAAACTTTTTAGT

712_501Mod CCCCCTACTAGTCGCATTGGTTTATCTCCAAAACATTACTGGGTCTCTAAACTTTTTAGT

705_501Mod CCCCCTACTAGTCGCATTGGTTTATCTCCAAAACATTACTGGGTCTCTAAACTTTTTAGT

711_501Mod CCCCCTACTAGTCGCATTGGTTTATCTCCAAAACATTACTGGGTCTCTAAACTTTTTAGT

702_508Mod CCCCCTACTAGTCGCATTGGTTTATCTCCAAAACATTACTGGGTCTCTAAACTTTTTAGT

Dama_mesopotamica TCCACTACTAGTTGCACTAGTCTACCTCCAAAACATTACTGGATCTCTAAACTTTCTAGT

Irish_Elk TCCACTACTAGTCGCACTAGTCTATCTCCAAAACATTACTGGATCTCTAAACTTTCTAGT

red_deer CCCACTACTAGTCGCATTAGTCTATCTCCAAAACATTACTGGGTCTCTAAACTTTCTAGT

** ******** *** * ** ** ***************** ************ ****

709_501Mod ACTCCAATACTGAATACAACCCCTATCCAACACCTGATCAAACATTTTTATATGACTAGC

702_501Mod ACTCCAATACTGAATACAACCCCTATCCAACACCTGATCAAACATTTTTATATGACTAGC

Eemian ACTCCAATACTGAATACAACCCCTATCCAATTCCTGATCAAACGTTTTCATATGACTAGC

710_501Mod ACTCCAATACTGAATACAACCCCTATCCAACACCTGATCAAACATTTTTATATGACTAGC

706_501Mod ACTCCAATACTGAATACAACCCCTATCCAACACCTGATCAAACATTTTTATATGACTAGC

707_501Mod ACTCCAATACTGAATACAACCCCTATCCAACACCTGATCAAACATTTTTATATGACTAGC

701_508Mod ACTCCAATACTGAATACAACCCCTATCCAACACCTGATCAAACATTTTTATATGACTAGC

712_501Mod ACTCCAATACTGAATACAACCCCTATCCAACACCTGATCAAACATTTTTATATGACTAGC

705_501Mod ACTCCAATACTGAATACAACCCCTATCCAACACCTGATCAAACATTTTTATATGACTAGC

711_501Mod ACTCCAATACTGAATACAACCCCTATCCAACACCTGATCAAACATTTTTATATGACTAGC

702_508Mod ACTCCAATACTGAATACAACCCCTATCCAACACCTGATCAAACATTTTTATATGACTAGC

Dama_mesopotamica ACTCCAATACTGAATACAACCCCTATCCAATTCCTGATCAAACGTTTTCATATGACTAGC

Irish_Elk ACTCCAATACTGAGTACAACCTCTATCCAACTCCTGATCAAATGTTTTCATATGACTAGC

red_deer ACTCCAATACTGAGTACAACCCCTACCTAACTCCTGATCAAACGTTTTCATGTGACTAGC

************* ******* *** * ** ********** **** ** ********

709_501Mod ATGCATAATAGCCTTCATGGTAAAAATACCACTATACGGCCTTCACCTTTGGCTACCTAA

702_501Mod ATGCATAATAGCCTTCATGGTAAAAATACCACTATACGGCCTTCACCTTTGGCTACCTAA

Eemian ATGCATAATAGCCTTTATAGTAAAAATACCACTATACGGTCTCCACCTTTGATTACCTAA

710_501Mod ATGCATAATAGCCTTCATGGTAAAAATACCACTATACGGCCTTCACCTTTGGCTACCTAA

706_501Mod ATGCATAATAGCCTTCATGGTAAAAATACCACTATACGGCCTTCACCTTTGGCTACCTAA

707_501Mod ATGCATAATAGCCTTCATGGTAAAAATACCACTATACGGCCTTCACCTTTGGCTACCTAA

701_508Mod ATGCATAATAGCCTTCATGGTAAAAATACCACTATACGGCCTTCACCTTTGGCTACCTAA

712_501Mod ATGCATAATAGCCTTCATGGTAAAAATACCACTATACGGCCTTCACCTTTGGCTACCTAA

705_501Mod ATGCATAATAGCCTTCATGGTAAAAATACCACTATACGGCCTTCACCTTTGGCTACCTAA

711_501Mod ATGCATAATAGCCTTCATGGTAAAAATACCACTATACGGCCTTCACCTTTGGCTACCTAA

702_508Mod ATGCATAATAGCCTTCATGGTAAAAATACCACTATACGGCCTTCACCTTTGGCTACCTAA

Dama_mesopotamica ATGCATAATAGCCTTTATAGTAAAAATACCACTATACGGTCTCCACCTTTGATTACCTAA

Irish_Elk ATGCATAATAGCCTTTATGGTAAAAATACCACTATATGGCCTCCACCTTTGACTACCTAA

red_deer ATGCATAATAGCTTTTATAGTAAAAATACCACTATATGGCCTCCATCTTTGACTACCCAA

************ ** ** ***************** ** ** ** ***** **** **

709_501Mod AGCCCATGTAGAAGCCCCTATTGCAGGCTCCATGGTCCTTGCAGCAATTCTACTAAAATT

702_501Mod AGCCCATGTAGAAGCCCCTATTGCAGGCTCCATGGTCCTTGCAGCAATTCTACTAAAATT

Eemian AGCCCATGTAGAAGCCCCTATTGCAGGCTCCATGGTCCTTGCAGCAATTCTACTAAAATT

710_501Mod AGCCCATGTAGAAGCCCCTATTGCAGGCTCCATGGTCCTTGCAGCAATTCTACTAAAATT

706_501Mod AGCCCATGTAGAAGCCCCTATTGCAGGCTCCATGGTCCTTGCAGCAATTCTACTAAAATT

707_501Mod AGCCCATGTAGAAGCCCCTATTGCAGGCTCCATGGTCCTTGCAGCAATTCTACTAAAATT

701_508Mod AGCCCATGTAGAAGCCCCTATTGCAGGCTCCATGGTCCTTGCAGCAATTCTACTAAAATT

712_501Mod AGCCCATGTAGAAGCCCCTATTGCAGGCTCCATGGTCCTTGCAGCAATTCTACTAAAATT

705_501Mod AGCCCATGTAGAAGCCCCTATTGCAGGCTCCATGGTCCTTGCAGCAATTCTACTAAAATT

711_501Mod AGCCCATGTAGAAGCCCCTATTGCAGGCTCCATGGTCCTTGCAGCAATTCTACTAAAATT

702_508Mod AGCCCATGTAGAAGCCCCTATTGCAGGCTCCATGGTCCTTGCAGCAATTCTACTAAAATT

Dama_mesopotamica AGCCCATGTAGAAGCTCCTATTGCAGGCTCTATGGTCCTTGCAGCAATTCTACTAAAACT

Irish_Elk AGCCCATGTAGAAGCCCCTATCGCAGGCTCTATGGTTCTCGCAGCAATTCTACTAAAATT

red_deer AGCCCATGTAGAAGCCCCTATTGCAGGTTCCATAGTCCTTGCAGCAATTCTACTAAAATT

*************** ***** ***** ** ** ** ** ****************** *

709_501Mod AGGAGGATACGGTATATTACGAATTACAACATTTTTAAACCCACTTACCGAATTTATAGC

702_501Mod AGGAGGATACGGTATATTACGAATTACAACATTTTTAAACCCACTTACCGAATTTATAGC

Eemian AGGAGGATACGGTATATTACGAATTACA-CATTTTTAAACCCACTTACCGAATT-ATAGC

710_501Mod AGGAGGATACGGTATATTACGAATTACAACATTTTTAAACCCACTTACCGAATTTATAGC

706_501Mod AGGAGGATACGGTATATTACGAATTACAACATTTTTAAACCCACTTACCGAATTTATAGC

707_501Mod AGGAGGATACGGTATATTACGAATTACAACATTTTTAAACCCACTTACCGAATTTATAGC

701_508Mod AGGAGGATACGGTATATTACGAATTACAACATTTTTAAACCCACTTACCGAATTTATAGC

712_501Mod AGGAGGATACGGTATATTACGAATTACAACATTTTTAAACCCACTTACCGAATTTATAGC

705_501Mod AGGAGGATACGGTATATTACGAATTACAACATTTTTAAACCCACTTACCGAATTTATAGC

711_501Mod AGGAGGATACGGTATATTACGAATTACAACATTTTTAAACCCACTTACCGAATTTATAGC

702_508Mod AGGAGGATACGGTATATTACGAATTACAACATTTTTAAACCCACTTACCGAATTTATAGC

Dama_mesopotamica AGGGGGATATGGCATGTTACGAATTACAACATTTTTAAACCCACTTACCGAATTCATAGC

Irish_Elk AGGAGGATNNNNNNTACTACGAATTACAACGTTTTTAAACCCACTTACCGAATTCATAGC

red_deer AGGAGGATATGGTATATTACGGATTACAACATTTTTAAATCCACTTACCGAATTCATAGC

*** **** * **** ****** * ******** ************** *****

709_501Mod ATACCCCTTTATTATACTGTCCTTATGAGGCATAATTATAACCAGCTCAATCTGCCTCCG

702_501Mod ATACCCCTTTATTATACTGTCCTTATGAGGCATAATTATAACCAGCTCAATCTGCCTCCG

Eemian ATACCCCTTTATTATACTGTCCTTATGAGGCATAATTATAACCAGCTCAATCTGCCTCCG

710_501Mod ATACCCCTTTATTATACTGTCCTTATGAGGCATAATTATAACCAGCTCAATCTGCCTCCG

706_501Mod ATACCCCTTTATTATACTGTCCTTATGAGGCATAATTATAACCAGCTCAATCTGCCTCCG

707_501Mod ATACCCCTTTATTATACTGTCCTTATGAGGCATAATTATAACCAGCTCAATCTGCCTCCG

701_508Mod ATACCCCTTTATTATACTGTCCTTATGAGGCATAATTATAACCAGCTCAATCTGCCTCCG

712_501Mod ATACCCCTTTATTATACTGTCCTTATGAGGCATAATTATAACCAGCTCAATCTGCCTCCG

705_501Mod ATACCCCTTTATTATACTGTCCTTATGAGGCATAATTATAACCAGCTCAATCTGCCTCCG

711_501Mod ATACCCCTTTATTATACTGTCCTTATGAGGCATAATTATAACCAGCTCAATCTGCCTCCG

702_508Mod ATACCCCTTTATTATACTGTCCTTATGAGGCATAATTATAACCAGCTCAATCTGCCTCCG

Dama_mesopotamica ATACCCCTTTATTATATTATCCTTATGAGGCATAATCATAACCAGCTCAATCTGCCTCCG

Irish_Elk ATACCCCTTTATTATACTATCCTTATGAGGCATAATTATAACCAGCTCAATCTGCCTCCG

red_deer ATATCCCTTTATTATACTGTCCTTATGAGGCATAATTATAACCAGCTCAATCTGCCTCCG

*** ************ * ***************** ***********************

709_501Mod CCAAACAGACCTTAAATCATTAATTGCTTACTCCTCCGTTAGTCACATAGCACTCGTTAT

702_501Mod CCAAACAGACCTTAAATCATTAATTGCTTACTCCTCCGTTAGTCACATAGCACTCGTTAT

Eemian CCAAACAGACCTTAAATCATTAATTGCTTACTCC--CGTTAGTCACATAGCACTCGTTAT

710_501Mod CCAAACAGACCTTAAATCATTAATTGCTTACTCCTCCGTTAGTCACATAGCACTCGTTAT

706_501Mod CCAAACAGACCTTAAATCATTAATTGCTTACTCCTCCGTTAGTCACATAGCACTCGTTAT

707_501Mod CCAAACAGACCTTAAATCATTAATTGCTTACTCCTCCGTTAGTCACATAGCACTCGTTAT

701_508Mod CCAAACAGACCTTAAATCATTAATTGCTTACTCCTCCGTTAGTCACATAGCACTCGTTAT

712_501Mod CCAAACAGACCTTAAATCATTAATTGCTTACTCCTCCGTTAGTCACATAGCACTCGTTAT

705_501Mod CCAAACAGACCTTAAATCATTAATTGCTTACTCCTCCGTTAGTCACATAGCACTCGTTAT

711_501Mod CCAAACAGACCTTAAATCATTAATTGCTTACTCCTCCGTTAGTCACATAGCACTCGTTAT

702_508Mod CCAAACAGACCTTAAATCATTAATTGCTTACTCCTCCGTTAGTCACATAGCACTCGTTAT

Dama_mesopotamica CCAAACAGACCTTAAATCATTAATTGCTTATTCTTCTGTTAGTCATATAGCACTTGTTAT

Irish_Elk CCAAACAGACCTTAAATCGTTAATTGCTTACTCCTCCGTCAGCCATATAGCACTTRTTAT

red_deer CCAGACAGACCTCAAATCATTAATTGCTTATTCCTCCGTTAGTCATATAGCACTTGTCAT

*** ******** ***** *********** ** ** ** ** ******** * **

709_501Mod TGTAGCTATTCTCATTCAAACACCTTGAAGCTATATAGGAGCCACGGCCCTAATAATCGC

702_501Mod TGTAGCTATTCTCATTCAAACACCTTGAAGCTATATAGGAGCCACGGCCCTAATAATCGC

Eemian TGTAGCTATTCTCATT-AAACACCTTGAAGCTATATAGGAGC-ACGGCCCTAATAATCGC

710_501Mod TGTAGCTATTCTCATTCAAACACCTTGAAGCTATATAGGAGCCACGGCCCTAATAATCGC

706_501Mod TGTAGCTATTCTCATTCAAACACCTTGAAGCTATATAGGAGCCACGGCCCTAATAATCGC

707_501Mod TGTAGCTATTCTCATTCAAACACCTTGAAGCTATATAGGAGCCACGGCCCTAATAATCGC

701_508Mod TGTAGCTATTCTCATTCAAACACCTTGAAGCTATATAGGAGCCACGGCCCTAATAATCGC

712_501Mod TGTAGCTATTCTCATTCAAACACCTTGAAGCTATATAGGAGCCACGGCCCTAATAATCGC

705_501Mod TGTAGCTATTCTCATTCAAACACCTTGAAGCTATATAGGAGCCACGGCCCTAATAATCGC

711_501Mod TGTAGCTATTCTCATTCAAACACCTTGAAGCTATATAGGAGCCACGGCCCTAATAATCGC

702_508Mod TGTAGCTATTCTCATTCAAACACCTTGAAGCTATATAGGAGCCACGGCCCTAATAATCGC

Dama_mesopotamica TGTAGCTATTCTCATTCAAACACCTTGAAGCTATATAGGGGCCACAGCCTTAATAATTGC

Irish_Elk CRTAGCTATCCTTATTCAAACACCTTGAAGCTATATGGGAGCCACAGCCCTA-TAACTCT

red_deer TGTAGCCATCCTCATTCAGACACCCTGAAGCTATATGGGAGCCAAGAACTGC-TAACTCT

**** ** ** *** * ***** *********** ** ** * * ***

709_501Mod CCACGGCTATACTCTCTTTATATATTAATCACAACACAACGAGGCAAATATACCCACCAT

702_501Mod CCACGGCTATACTCTCTTTATATATTAATCACAACACAACGAGGCAAATATACCCACCAT

Eemian CCACG-CTATACTCTCTTTATATATTAATCACAACACAACGAGGCAAATATACCCACCAT

710_501Mod CCACGGCTATACTCTCTTTATATATTAATCACAACACAACGAGGCAAATATACCCACCAT

706_501Mod CCACGGCTATACTCTCTTTATATATTAATCACAACACAACGAGGCAAATATACCCACCAT

707_501Mod CCACGGCTATACTCTCTTTATATATTAATCACAACACAACGAGGCAAATATACCCACCAT

701_508Mod CCACGGCTATACTCTCTTTATATATTAATCACAACACAACGAGGCAAATATACCCACCAT

712_501Mod CCACGGCTATACTCTCTTTATATATTAATCACAACACAACGAGGCAAATATACCCACCAT

705_501Mod CCACGGCTATACTCTCTTTATATATTAATCACAACACAACGAGGCAAATATACCCACCAT

711_501Mod CCACGGCTATACTCTCTTTATATATTAATCACAACACAACGAGGCAAATATACCCACCAT

702_508Mod CCACGGCTATACTCTCTTTATATATTAATCACAACACAACGAGGCAAATATACCCACCAT

Dama_mesopotamica TCAC--------------------------------------------------------

Irish_Elk ATGC--------------------------------------------------------

red_deer ATGC--------------------------------------------------------

*

709_501Mod ATCAACAACATCTCGCCCTCCTTTACACGAGAAAATGCCCTCATATCATTACATCCCCGT

702_501Mod ATCAACAACATCTCGCCCTCCTTTACACGAGAAAATGCCCTCATATCATTACATCCCCGT

Eemian ATCAACAACATCTCGCCCTCCTTTACACGAGAAAATGCCCTCATATCA------CCCCGT

710_501Mod ATCAACAACATCTCGCCCTCCTTTACACGAGAAAATGCCCTCATATCATTACATCCCCGT

706_501Mod ATCAACAACATCTCGCCCTCCTTTACACGAGAAAATGCCCTCATATCATTACATCCCCGT

707_501Mod ATCAACAACATCTCGCCCTCCTTTACACGAGAAAATGCCCTCATATCATTACATCCCCGT

701_508Mod ATCAACAACATCTCGCCCTCCTTTACACGAGAAAATGCCCTCATATCATTACATCCCCGT

712_501Mod ATCAACAACATCTCGCCCTCCTTTACACGAGAAAATGCCCTCATATCATTACATCCCCGT

705_501Mod ATCAACAACATCTCGCCCTCCTTTACACGAGAAAATGCCCTCATATCATTACATCCCCGT

711_501Mod ATCAACAACATCTCGCCCTCCTTTACACGAGAAAATGCCCTCATATCATTACATCCCCGT

702_508Mod ATCAACAACATCTCGCCCTCCTTTACACGAGAAAATGCCCTCATATTATTACATCCCCGT

Dama_mesopotamica -------------------------------------------------------CCCAT

Irish_Elk ------------------------------------------------------CCCCGT

red_deer ------------------------------------------------------CCCCGT

*** *

709_501Mod GCATAATAGCACGGCTTTTTCGAACTTTTAGAGGATGACAGAAATCCGTTGGTCTTAGGA

702_501Mod GCATAATAGCACGGCTTTTTCGAACTTTTAGAGGATGACAGAAATCCGTTGGTCTTAGGA

Eemian GCATAATAGCACGGCTTTTTCGAACTTTTAGAGGATGACAGAAATCCGTTGGTCTTAGGA

710_501Mod GCATAATAGCACGGCTTTTTCGAACTTTTAGAGGATGACAGAAATCCGTTGGTCTTAGGA

706_501Mod GCATAATAGCACGGCTTTTTCGAACTTTTAGAGGATGACAGAAATCCGTTGGTCTTAGGA

707_501Mod GCATAATAGCACGGCTTTTTCGAACTTTTAGAGGATGACAGAAATCCGTTGGTCTTAGGA

701_508Mod GCATAATAGCACGGCTTTTTCGAACTTTTAGAGGATGACAGAAATCCGTTGGTCTTAGGA

712_501Mod GCATAATAGCACGGCTTTTTCGAACTTTTAGAGGATGACAGAAATCCGTTGGTCTTAGGA

705_501Mod GCATAATAGCACGGCTTTTTCGAACTTTTAGAGGATGACAGAAATCCGTTGGTCTTAGGA

711_501Mod GCATAATAGCACGGCTTTTTCGAACTTTTAGAGGATGACAGAAATCCGTTGGTCTTAGGA

702_508Mod GCATAATAGCACGGCTTTTTCGAACTTTTAGAGGATGACAGAAATCCGTTGGTCTTAGGA

Dama_mesopotamica GTATAATAACATGGCTTTTTCGAACTTTTAGAGGATGACAGAAATCCGTTGGTCTTAGGA

Irish_Elk GTATAATAACACGGCTTTTTCGAACTTTTAGAGGATGGCAGGAATCCGTTGGTCTTAGGA

red_deer GTATAATAACACGGCTTTTTCGAACTTTTAGAGGATGACAGAAATCCGTTGGTCTTAGGA

* ****** ** ************************* *** ******************

709_501Mod ACCAAAAAATTGGTGCAACTCCAAATAAAAGTAATAAACCTATTCTCTTCCTTTGCACTA

702_501Mod ACCAAAAAATTGGTGCAACTCCAAATAAAAGTAATAAACCTATTCTCTTCCTTTGCACTA

Eemian ACCAAAAAATTGGTGCAACTCCAAATAAAAGTAATAAACCTATTCTCTTC----------

710_501Mod ACCAAAAAATTGGTGCAACTCCAAATAAAAGTAATAAACCTATTCTCTTCCTTTGCACTA

706_501Mod ACCAAAAAATTGGTGCAACTCCAAATAAAAGTAATAAACCTATTCTCTTCCTTTGCACTA

707_501Mod ACCAAAAAATTGGTGCAACTCCAAATAAAAGTAATAAACCTATTCTCTTCCTTTGCACTA

701_508Mod ACCAAAAAATTGGTGCAACTCCAAATAAAAGTAATAAACCTATTCTCTTCCTTTGCACTA

712_501Mod ACCAAAAAATTGGTGCAACTCCAAATAAAAGTAATAAACCTATTCTCTTCCTTTGCACTA

705_501Mod ACCAAAAAATTGGTGCAACTCCAAATAAAAGTAATAAACCTATTCTCTTCCTTTGCACTA

711_501Mod ACCAAAAAATTGGTGCAACTCCAAATAAAAGTAATAAACCTATTCTCTTCCTTTGCACTA

702_508Mod ACCAAAAAATTGGTGCAACTCCAAATAAAAGTAATAAACCTATTCTCTTCCTTTGCACTA

Dama_mesopotamica ACCAAAAAATTGGTGCAACTCCAAATAAAAGTAATAAACCTATTCTCCTCCTTTGCATTA

Irish_Elk ACCAAAAAATTGGTGCAACTCCAAATAAAAGTAATAAATCTATTCTCTTCCTTTACACTA

red_deer ACCAAAAAATTGGTGCAACTCCAAATAAAAGTAATAAACCTATTCTCCTCCTTTACACTA

************************************** ******** **

709_501Mod ATCACCCTACTACTACTAATTATTCCCATCATAACCACAAGTTCTGACAACTATAAAACC

702_501Mod ATCACCCTACTACTACTAATTATTCCCATCATAACCACAAGTTCTGACAACTATAAAACC

Eemian ---ACCCTACTAATACTAATTATTCCCATCATAACCACAAGTTCTGACAATTATAAAACC

710_501Mod ATCACCCTACTACTACTAATTATTCCCATCATAACCACAAGTTCTGACAACTATAAAACC

706_501Mod ATCACCCTACTACTACTAATTATTCCCATCATAACCACAAGTTCTGACAATTATAAAACC

707_501Mod ATCACCCTACTACTACTAATTATTCCCATCATAACCACAAGTTCTGACAATTATAAAACC

701_508Mod ATCACCCTACTACTACTAATTATTCCCATCATAACCACAAGTTCTGACAATTATAAAACC

712_501Mod ATCACCCTACTACTACTAATTATTCCCATCATAACCACAAGTTCTGACAATTATAAAACC

705_501Mod ATCACCCTACTACTACTAATTATTCCCATCATAACCACAAGTTCTGACAATTATAAAACC

711_501Mod ATCACCCTACTACTACTAATTATTCCCATCATAACCACAAGTTCTGACAATTATAAAACC

702_508Mod ATCACCCTACTACTACTAATTATTCCCATCATAACCACAAGTTCTGACAATTATAAAACC

Dama_mesopotamica GTTACCCTATTACTATTAACTATTCCCATCATAACCACAAACTTTAACAATCACAAAACT

Irish_Elk GTTACCCTACTACTACTAACAATCCCCATCATAACTACAAGTCCCAACAACTATAAGTCC

red_deer GTTACCCTACTACTACTAACTATCCCCATCATTATTACAAGTTCTGACAACTATAAAGCT

****** ** ** *** ** ******** * **** **** * ** *

709_501Mod TCTAACTATCCACTCTACGTAAAAACAACTATCTCATTCCCACAATAATATTCATTCACA

702_501Mod TCTAACTATCCACTCTACGTAAAAACAACTATCTCATTCCCACAATAATATTCATTCACA

Eemian TCTAACTATCCACTCTACGTAAAAACAACTATCTCAT----------ATATTCATTCACA

710_501Mod TCTAACTATCCACTCTACGTAAAAACAACTATCTCATTCCCACAATAATATTCATTCACA

706_501Mod TCTAACTATCCACTCTACGTAAAAACAACTATCTCATTCCCACAATAATATTCATTCACA

707_501Mod TCTAACTATCCACTCTACGTAAAAACAACTATCTCATTCCCACAATAATATTCATTCACA

701_508Mod TCTAACTATCCACTCTACGTAAAAACAACTATCTCATTCCCACAATAATATTCATTCACA

712_501Mod TCTAACTATCCACTCTACGTAAAAACAACTATCTCATTCCCACAATAATATTCATTCACA

705_501Mod TCTAACTATCCACTCTACGTAAAAACAACTATCTCATTCCCACAATAATATTCATTCACA

711_501Mod TCTAACTATCCACTCTACGTAAAAACAACTATCTCATTCCCACAATAATATTCATTCACA

702_508Mod TCTAACTATCCACTCTACGTAAAAACAACTATCTCATTCCCACAATAATATTCATTCACA

Dama_mesopotamica TCTAACTATCCACTTTACGTAAAAACAACTATTTCATTCCCACAATAATATTTATCCATA

Irish_Elk TCTAATTATCCACTCTACGTAAAAACACTAGCATAATTCCCACAATAATATTTATTCACA

red_deer TCTAATTACCCACTCTAGCTTTTATCACTAGTATAATTCCCACAATAATATTTATTCATA

***** ** ***** ** * * ** * ** ***** ** ** *

709_501Mod CTGGCCAAGAAATAATTATCTCAAACTGACACTGATTAACTATTCAAACTATTAAATTAA

702_501Mod CTGGCCAAGAAATAATTATCTCAAACTGACACTGATTAACTATTCAAACTATTAAATTAA

Eemian CTGGCCAAGAAATAATTATCTCAAACTGACACTGATTAACTATTCAAACTATTAAATTAA

710_501Mod CTGGCCAAGAAATAATTATCTCAAACTGACACTGATTAACTATTCAAACTATTAAATTAA

706_501Mod CTGGCCAAGAAATAATTATCTCAAACTGACACTGATTAACTATTCAAACTATTAAATTAA

707_501Mod CTGGCCAAGAAATAATTATCTCAAACTGACACTGATTAACTATTCAAACTATTAAATTAA

701_508Mod CTGGCCAAGAAATAATTATCTCAAACTGACACTGATTGACTATTCAAACTATTAAATTAA

712_501Mod CTGGCCAAGAAATAATTATCTCAAACTGACACTGATTAACTATTCAAACTATTAAATTAA

705_501Mod CTGGCCAAGAAATAATTATCTCAAACTGACACTGATTAACTATTCAAACTATTAAATTAA

711_501Mod CTGGCCAAGAAATAATTATCTCAAACTGACACTGATTAACTATTCAAACTATTAAATTAA

702_508Mod CTGGCCAAGAAATAATTATCTCAAACTGACACTGATTAACTATTCAAACTATTAAATTAA

Dama_mesopotamica CCGGCCAAGAAATAATTATCTCAAACTGACACTGATTAACTATTCAAACTATTAAACTAT

Irish_Elk CCGGCCAAGAAATAATTATTTCAAACTGACACTGACTGACTATTCAAACTATCAAACTAT

red_deer CTGGCCAAGAAATAATTATCTCAAACTGACACTGATTAACTATCCAAACTATCAAACTAT

* ***************** *************** * ***** ******** *** **

709_501Mod CACTCAGCTTCAAAATAGATTATTTCTCAATAATATTTGTACCTCCACCCACTAACAGAG

702_501Mod CACTCAGCTTCAAAATAGATTATTTCTCAATAATATTTGTACCTCCACCCACTAACAGAG

Eemian CACTCAGCTTCAAAATAGATTATTTCTCAATAATATTTGTACC--CACCCACTAACAGAG

710_501Mod CACTCAGCTTCAAAATAGATTATTTCTCAATAATATTTGTACCTCCACCCACTAACAGAG

706_501Mod CACTCAGCTTCAAAATAGATTATTTCTCAATAATATTTGTACCTCCACCCACTAACAGAG

707_501Mod CACTCAGCTTCAAAATAGATTATTTCTCAATAATATTTGTACCTCCACCCACTAACAGAG

701_508Mod CACTCAGCTTCAAAATAGATTATTTCTCAATAATATTTGTACCTCCACCCACTAACAGAG

712_501Mod CACTCAGCTTCAAAATAGATTATTTCTCAATAATATTTGTACCTCCACCCACTAACAGAG

705_501Mod CACTCAGCTTCAAAATAGATTATTTCTCAATAATATTTGTACCTCCACCCACTAACAGAG

711_501Mod CACTCAGCTTCAAAATAGATTATTTCTCAATAATATTTGTACCTCCACCCACTAACAGAG

702_508Mod CACTCAGCTTCAAAATAGATTATTTCTCAATAATATTTGTACCTCCACCCACTAACAGAG

Dama_mesopotamica CACTTAGCTTCAAAATAGATTATTTCTCAATAATATTCGTACC-CCATCCACTAACAGAA

Irish_Elk CACTCAGCTTCAAAATAGACTATTTCTCAATACTAATC-CGCTTCCACCCACTGACAGAA

red_deer CACTTAGCTTCAAAATAGATTATATTTTCCTATTAATC-CGTTTCCATCCACTGACAGAA

**** ************** *** * * ** ** * ** ***** *****

709_501Mod AACAATAAATTCGCACAATCCATTCTACTATGTCTAGGGGCTATTACTACTCTATTTACA

702_501Mod AACAATAAATTTGCACAATCCATTCTACTATGTCTAGGGGCTATTACTACTCTATTTACA

Eemian AACAATAAATTCGCACAATCCATTCTACTATGTCTAGGGGCTATTACTACTCTATTTACA

710_501Mod AACAATAAATTCGCACAATCCATTCTACTATGTCTAGGGGCTATTACTACTCTATTTACA

706_501Mod AACAATAAATTCGCACAATCCATTCTACTATGTCTAGGGGCTATTACTACTCTATTTACA

707_501Mod AACAATAAATTCGCACAATCCATTCTACTATGTCTAGGGGCTATTACTACTCTATTTACA

701_508Mod AACAATAAATTCGCACAATCCATTCTACTATGTCTAGGGGCTATTACTACTCTATTTACA

712_501Mod AACAATAAATTCGCACAATCCATTCTACTATGTCTAGGGGCTATTACTACTCTATTTACA

705_501Mod AACAATAAATTCGCACAATCCATTCTACTATGTCTAGGGGCTATTACTACTCTATTTACA

711_501Mod AACAATAAATTCGCACAATCCATTCTACTATGTCTAGGGGCTATTACTACTCTATTTACA

702_508Mod AACAATAAATTCGCACAATCCATTCTACTATGTCTAGGGGCTATTACTACTCTATTTACA

Dama_mesopotamica AACAACAAATTTGCACAATCTACCCTATTATGCCTAGGAGCTATTACCACTCTATTTACA

Irish_Elk AACAACAAATTTGCACAATCTGTCCTACTATGTCTAGGGGCTATTACTACCCTATTTACA

red_deer AACAACAAGTTTGCACAATCCATCCTACTATGCCTAGGGGCTATTACTACCCTATTTACA

***** ** ** ******** *** **** ***** ******** ** *********

709_501Mod GCAATATGTGCTCTTACCCAGAATGATATCAAAAAA-ATTATTGCTTTTTGTTCTGGCTC

702_501Mod GCAATATGTGCTCTTACCCAGAATGATATCAAAAAA-ATTATTGCTTTTTGTTCTGGCTC

Eemian GCAATATGTGCTCTTACCCAGAATGATATCAAAAAA-ATTATTGCTTTT-GTTCTGGCTC

710_501Mod GCAATATGTGCTCTTACCCAGAATGATATCAAAAAA-ATTATTGCTTTTTGTTCTGGCTC

706_501Mod GCAATATGTGCTCTTACCCAGAATGATATCAAAAAA-ATTATTGCTTTTTGTTCTGGCTC

707_501Mod GCAATATGTGCTCTTACCCAGAATGATATCAAAAAA-ATTATTGCTTTTTGTTCTGGCTC

701_508Mod GCAATATGTGCTCTTACCCAGAATGATATCAAAAAA-ATTATTGCTTTTTGTTCTGGCTC

712_501Mod GCAATATGTGCTCTTACCCAGAATGATATCAAAAAA-ATTATTGCTTTTTGTTCTGGCTC

705_501Mod GCAATATGTGCTCTTACCCAGAATGATATCAAAAAA-ATTATTGCTTTTTGTTCTGGCTC

711_501Mod GCAATATGTGCTCTTACCCAGAATGATATCAAAAAA-ATTATTGCTTTTTGTTCTGGCTC

702_508Mod GCAATATGTGCTCTTACCCAGAATGATATCAAAAAA-ATTATTGCTTTTTGTTCTGGCTC

Dama_mesopotamica GCAATATGTGCCCTTACCCAAAATGATATCAAAAAA-ATTATCGCTTTTT-CTCTGGTTC

Irish_Elk GCAATATGTGCCCTTACCCAAAATGACATCAAAAAAAACTATTCATAT--GCTCTGGCTC

red_deer GCGATATGTGCTCTCACCCAGAATGATATAAAGCCATATTATTCATAT--GCTCCGGCTC

** ******** ** ***** ***** ** ** * * *** * * ** ** **

709_501Mod TATTATCCACAGCCTAAATGACGAACAAGACATCCGAAAAATAGGAGGCTTATTTAAAGC

702_501Mod TATTATCCACAGCCTAAATGACGAACAAGACATCCGAAAAATAGGAGGCTTATTTAAAGC

Eemian TATTATCCACAGCCTAAATGACGAACAAGACATCCGAAAAATAGGAGGCTTATTTAAAGC

710_501Mod TATTATCCACAGCCTAAATGACGAACAAGACATCCGAAAAATAGGAGGCTTATTTAAAGC

706_501Mod TATTATCCACAGCCTAAATGACGAACAAGACATCCGAAAAATAGGAGGCTTATTTAAAGC

707_501Mod TATTATCCACAGCCTAAATGACGAACAAGACATCCGAAAAATAGGAGGCTTATTTAAAGC

701_508Mod TATTATCCACAGCCTAAATGACGAACAAGACATCCGAAAAATAGGAGGCTTATTTAAAGC

712_501Mod TATTATCCACAGCCTAAATGACGAACAAGACATCCGAAAAATAGGAGGCTTATTTAAAGC

705_501Mod TATTATCCACAGCCTAAATGACGAACAAGACATCCGAAAAATAGGAGGCTTATTTAAAGC

711_501Mod TATTATCCACAGCCTAAATGACGAACAAGACATCCGAAAAATAGGAGGCTTATTTAAAGC

702_508Mod TATTATCCACAGCCTAAATGACGAACAAGACATCCGAAAAATAGGAGGCTTATTTAAAGC

Dama_mesopotamica TATTATCCACAGCCTAAATGACGAACAAGATATTCGAAAAATAGGAGGCTTATTTAAAGC

Irish_Elk TATCATCCACAGCCTAAACGATGAACAAGACATTCGAAAAATAGGAGGCTTATTTAAAGC

red_deer TATCATCCACAGCCTAAATGACGAACAAGACATTCGAAAAATAGGGGGCCTATTTAAAGC

*** ************** ** ******** ** *********** *** **********

709_501Mod TATACCATTTACCACAACAGCCTTAATCATTGGCAGTCTTGCACTAACAGGAGTACCT--

702_501Mod TATACCATTTACCACAACAGCCCTAATCATTGGCAGTCTTGCACTAACAGGAGTACCT--

Eemian TATACCATTTACCACAACAGCCCTAATCATTGGCAGTCTTGCACTAACAGGAGTACCT--

710_501Mod TATACCATTTACCACAACAGCCTTAATCATTGGCAGTCTTGCACTAACAGGAGTACCT--

706_501Mod TATACCATTTACCACAACAGCCCTAATCATTGGCAGTCTTGCACTAACAGGAGTACCT--

707_501Mod TATACCATTTACCACAACAGCCCTAATCATTGGCAGTCTTGCACTAACAGGAGTACCT--

701_508Mod TATACCATTTACCACAACAGCCCTAATCATTAGCAGTCTTGCACTAACAGGAGTACCT--

712_501Mod TATACCATTTACCACAACAGCCCTAATCATTGGCAGTCTTGCACTAACAGGAGTACCT--

705_501Mod TATACCATTTACCACAACAGCCCTAATCATTGGCAGTCTTGCACTAACAGGAGTACCT--

711_501Mod TATACCATTTACCACAACAGCCCTAATCATTGGCAGTCTTGCACTAACAGGAGTACCT--

702_508Mod TATACCATTTACCACAACAGCCCTAATCATTGGCAGTCTTGCACTAACAGGAGTACCT--

Dama_mesopotamica CATACCATTTACCACAACAGCCCTAATCATTGGCAGCCTCGCACTAACAGGAATACCC--

Irish_Elk CATACCATTTACCACAACAGCCCTAATTATTGGCAGCCTCGCACTAACAGGAACATTTCC

red_deer CATGCCATTCACCACAACAGCCCTAATCATTGGCAGCCTCGCACTGGATTTATTATTTCC

** ***** ************ **** *** **** ** ***** * *

709_501Mod ----------------------TTCCTCA-------------------------------

702_501Mod ----------------------TTCCTCA-------------------------------

Eemian ----------------------TTCCTC--------------------------------

710_501Mod ----------------------TTCCTCA-------------------------------

706_501Mod ----------------------TTCCTCA-------------------------------

707_501Mod ----------------------TTCCTCA-------------------------------

701_508Mod ----------------------TTCCTCA-------------------------------

712_501Mod ----------------------TTCCTCA-------------------------------

705_501Mod ----------------------TTCCTCA-------------------------------

711_501Mod ----------------------TTCCTCA-------------------------------

702_508Mod ----------------------TTCCTCA-------------------------------

Dama_mesopotamica ----------------------TTCCTCA-------------------------------

Irish_Elk AATAACATTCCTCCAACAACAATTCCCCAATTAACAATACCTTATCACCTAAAAATAATC

red_deer AACAACATTCCTCCAACAACAATTCCTCAACTAACGATACCTTATTAC-TGAAGAAAATC

**** *

709_501Mod ---------CAAGCACAAAAATAATACTCAAGATAAATACAATGTATGTTATCATTATTC

702_501Mod ---------CAAGCACAAAAATAATACTCAAGATAAATACAATGTATGTTATCATTATTC

Eemian ---------------CAAAAATAACACTTAAAATAAATACAATGTACGTTATCATTATTC

710_501Mod ---------CAAGCACAAAAATAATACTCAAGATAAATACAATGTATGTTATCATTATTC

706_501Mod ---------CAAGCACAAAAATAATACTCAAGATAAATACAATGTATGTTATCATTATTC

707_501Mod ---------CAAGCACAAAAATAATACTCAAGATAAATACAATGTATGTTATCATTATTC

701_508Mod ---------CAAGCACAAAAATAATACTCAAGATAAATACAATGTATGTTATCATTATTC

712_501Mod ---------CAAGCACAAAAATAATACTCAAGATAAATACAATGTATGTTATCATTATTC

705_501Mod ---------CAAGCACAAAAATAATACTCAAGATAAATACAATGTATGTTATCATTATTC

711_501Mod ---------CAAGCACAAAAATAATACTCAAGATAAATACAATGTATGTTATCATTATTC

702_508Mod ---------CAAGCACAAAAATAATACTCAAGATAAATACAATGTATGTTATCATTATTC

Dama_mesopotamica ---------CAAGCACAAAAATAATACTTAAGATAAATACAATGTATGTTATCATTATTC

Irish_Elk CTACAAAACCAAGCACAAAAAGAATACTCAAGATAAATACAATGTATGTTATCATTATTC

red_deer CCACAAACCCAAGCACAAAAATGATACTTAAGATAAATACAATGTATGTTATCATTATTC

****** * *** ** ************** *************

709_501Mod TCACATGGAATCTAACCATGACTAATGATATGAAAAACCATCGTTGTCATTCAACTACAA

702_501Mod TCACATGGAATCTAACCATGACTAATGATATGAAAAACCATCGTTGTCATTCAACTACAA

Eemian TCACATGGAATCTAACCATGACCAATGATATGAAAAACCATCGTTGTAATTCAACTATAA

710_501Mod TCACATGGAATCTAACCATGACTAATGATATGAAAAACCATCGTTGTCATTCAACTACAA

706_501Mod TCACATGGAATCTAACCATGACTAATGATATGAAAAACCATCGTTGTCATTCAACTACAA

707_501Mod TCACATGGAATCTAACCATGACTAATGATATGAAAAACCATCGTTGTCATTCAACTACAA

701_508Mod TCACATGGAATCTAACCATGACTAATGATATGAAAAACCATCGTTGTCATTCAACTACAA

712_501Mod TCACATGGAATCTAACCATGACTAATGATATGAAAAACCATCGTTGTCATTCAACTACAA

705_501Mod TCACATGGAATCTAACCATGACTAATGATATGAAAAACCATCGTTGTCATTCAACTACAA

711_501Mod TCACATGGAATCTAACCATGACTAATGATATGAAAAACCATCGTTGTCATTCAACTACAA

702_508Mod TCACATGGAATCTAACCATGACTAATGATATGAAAAACCATCGTTGTCATTCAACTACAA

Dama_mesopotamica TCACATGGAATCTAACCATGACTAATGATATGAAAAACCATCGTTGTCATTCAACTATAA

Irish_Elk TCACATGGAATCTAACCATGACTAATGATATGAAAAACCATCGTTGTCATTCAACTACAA

red_deer TCACATGGAATCTAACCATGACTAATGATATGAAAAACCATCGTTGTCATTCAACTACAA

********************** ************************ ********* **

709_501Mod GAACACTAATGATCAATATCCGAAAAACTCACCCATTGATAAAAATCGTAAACAACGCAT

702_501Mod GAACACTAATGATCAATATCCGAAAAACTCACCCATTGATAAAAATCGTAAACAACGCAT

Eemian GAACACTAATGACCAATATTCGAAAGACACACCCATTGATAAAAATCGTAAACAACGCAT

710_501Mod GAACACTAATGATCAATATCCGAAAAACTCACCCATTGATAAAAATCGTAAACAACGCAT

706_501Mod GAACACTAATGATCAATATCCGAAAAACTCACCCATTGATAAAAATCGTAAACAACGCAT

707_501Mod GAACACTAATGATCAATATCCGAAAAACTCACCCATTGATAAAAATCGTAAACAACGCAT

701_508Mod GAACACTAATGATCAATATCCGAAAAACTCACCCATTGATAAAAATCGTAAACAACGCAT

712_501Mod GAACACTAATGATCAATATCCGAAAAACTCACCCATTGATAAAAATCGTAAACAACGCAT

705_501Mod GAACACTAATGATCAATATCCGAAAAACTCACCCATTGATAAAAATCGTAAACAACGCAT

711_501Mod GAACACTAATGATCAATATCCGAAAAACTCACCCATTGATAAAAATCGTAAACAACGCAT

702_508Mod GAACACTAATGATCAATATCCGAAAAACTCACCCATTGATAAAAATCGTAAACAACGCAT

Dama_mesopotamica GAACACTAATGATCAATATCCGAAAAACCCACCCATTAATAAAAATCGTAAATAACGCAT

Irish_Elk GAACACTAATGACCAATATCCGAAAAACCCACCCACTAATAAAAATCGTAAACAACGCAT

red_deer GAACACTAATGACCAATATCCGAAAAACCCACCCACTAATAAAAATTGTAAACAACGCAT

************ ****** ***** ** ****** * ******** ***** *******

709_501Mod TTATTGATCTCCCAGCCCCATCAAATATTT-CATCCTG--ATGAAATTTTGGCTCCCTAC

702_501Mod TTATTGATCTCCCAGCCCCATCAAATATTT-CATCCTG--ATGAAATTTTGGCTCCCTAC

Eemian TTATTGATCTCCCAGCCCCATCAAATATTT-CATCCTG--ATGAAATTTTGGCTCCCTAC

710_501Mod TTATTGATCTCCCAGCCCCATCAAATATTT-CATCCTG--ATGAAATTTTGGCTCCCTAC

706_501Mod TTATTGATCTCCCAGCCCCATCAAATATTT-CATCCTG--ATGAAATTTTGGCTCCCTAC

707_501Mod TTATTGATCTCCCAGCCCCATCAAATATTT-CATCCTG--ATGAAATTTTGGCTCCCTAC

701_508Mod TTATTGATCTCCCAGCCCCATCAAATATTT-CATCCTG--ATGAAATTTTGGCTCCCTAC

712_501Mod TTATTGATCTCCCAGCCCCATCAAATATTT-CATCCTG--ATGAAATTTTGGCTCCCTAC

705_501Mod TTATTGATCTCCCAGCCCCATCAAATATTT-CATCCTG--ATGAAATTTTGGCTCCCTAC

711_501Mod TTATTGATCTCCCAGCCCCATCAAATATTT-CATCCTG--ATGAAATTTTGGCTCCCTAC

702_508Mod TTATTGATCTCCCAGCCCCATCAAATATTT-CATCCTG--ATGAAATTTTGGCTCCCTAC

Dama_mesopotamica TTATCGACCTCCCAGCCCCATCAAATATTT-CATCCTG--ATG--------GCTCCCTAC

Irish_Elk TCATTGATCTCCCAGCCCCATCAAATATTT-CATCCTGAGATGAAATTTCGGCTCCCTAT

red_deer TTATTGACCTCCCAGCCCCATCAAATATTTTCATCCTG--ATGAAATTTCGGCTCATTAC

* ** ** ********************** ******* *** **** **

709_501Mod TAGGAATTTGCTTAATCCTACAAATCCTCACAGGCCTATTCCTAGCAATACACTACACAT

702_501Mod TAGGAATTTGCTTAATCCTACAAATCCTCACAGGCCTATTCCTAGCAATACACTACACAT

Eemian TAGGAATTTGCTTAATCCTACAAATCCTCACAGGCCTATTCCTAGCAATACACTACACAT

710_501Mod TAGGAATTTGCTTAATCCTACAAATCCTCACAGGCCTATTCCTAGCAATACACTACACAT

706_501Mod TAGGAATTTGCTTAATCCTACAAATCCTCACAGGCCTATTCCTAGCAATACACTACACAT

707_501Mod TAGGAATTTGCTTAATCCTACAAATCCTCACAGGCCTATTCCTAGCAATACACTACACAT

701_508Mod TAGGAATTTGCTTAATCCTACAAATCCTCACAGGCCTATTCCTAGCAATACACTACACAT

712_501Mod TAGGAATTTGCTTAATCCTACAAATCCTCACAGGCCTATTCCTAGCAATACACTACACAT

705_501Mod TAGGAATTTGCTTAATCCTACAAATCCTCACAGGCCTATTCCTAGCAATACACTACACAT

711_501Mod TAGGAATTTGCTTAATCCTACAAATCCTCACAGGCCTATTCCTAGCAATACACTACACAT

702_508Mod TAGGAATTTGCTTAATCCTACAAATCCTCACAGGCCTATTCCTAGCAATACACTACACAT

Dama_mesopotamica TAGGAATTTGCTTAATCCTACAAATTCTCACAGGCCTATTCCTAGCAATGCACTACACAT

Irish_Elk TAGGAATTTGCTTAATTTTACAAATCCTTACAGGCCTATTCCTAGCGAT--AATATCATT

red_deer TAGGAGTCTGTCTAATCCTACAAATCCTCACAGGCCTATTCTGAGGACA--AATATCATT

***** * ** **** ******* ** ************ ** * ** *

709_501Mod C-------TGAGGAGCAACAGTTATTACCAATCTTCTCTCGGCAATCCCATACATTGGTA

702_501Mod C-------TGAGGAGCAACAGTTATTACCAATCTTCTCTCGGCAATCCCATACATTGGTA

Eemian --------TGAGGGGCAACAGTCATTACCAACCTTCTCTCAGCAATCCCATATATTGGTA

710_501Mod C-------TGAGGAGCAACAGTTATTACCAATCTTCTCTCGGCAATCCCATACATTGGTA

706_501Mod C-------TGAGGAGCAACAGTTATTACCAATCTTCTCTCGGCAATCCCATACATTGGTA

707_501Mod C-------TGAGGAGCAACAGTTATTACCAATCTTCTCTCGGCAATCCCATACATTGGTA

701_508Mod C-------TGAGGAGCAACAGTTATTACCAATCTTCTCTCAGCAATCCCATACATTGGTA

712_501Mod C-------TGAGGAGCAACAGTTATTACCAATCTTCTCTCGGCAATCCCATACATTGGTA

705_501Mod C-------TGAGGAGCAACAGTTATTACCAATCTTCTCTCGGCAATCCCATACATTGGTA

711_501Mod C-------TGAGGAGCAACAGTTATTACCAATCTTCTCTCGGCAATCCCATACATTGGTA

702_508Mod C-------TGAGGAGCAACAGTTATTACCAATCTTCTCTCGGCAATCCCATATATTGGTA

Dama_mesopotamica CAAATTTTGGAGGGGCAACAGTCATTACCAACCTTCTCTCAGCAATCCCATATATTGGTA

Irish_Elk C-------TGAGGAGCAACGGTCATTACTAATCTTCTCTCAGCAATTCCATACATTGGTA

red_deer C-------TGAGGAGCAACAGTCATCACCAACCTTCTCTCAGCAATTCCATATATTGGGA

**** ***** ** ** ** ** ******** ***** ***** ***** *

709_501Mod CAAACCTAGTTGAATGAATCTGAGCCATTAAAGATATTTTAGGCATCCTATTCCTATTTC

702_501Mod CAAACCTAGTTGAATGAATCTGAGCCATTAAAGATATTTTAGGCATCCTATTCCTATTTC

Eemian CAAACCTAGTCGAATGAATCTGA-CCATTAAAGATATTTTAGGCATCCTATTCCTATTTC

710_501Mod CAAACCTAGTTGAATGAATCTGAGCCATTAAAGATATTTTAGGCATCCTATTCCTATTTC

706_501Mod CAAACCTAGTTGAATGAATCTGAGCCATTAAAGATATTTTAGGCATCCTATTCCTATTTC

707_501Mod CAAACCTAGTTGAATGAATCTGAGCCATTAAAGATATTTTAGGCATCCTATTCCTATTTC

701_508Mod CAAACCTAGTTGAATGAATCTGAGCCATTAAAGATATTTTAGGCATCCTATTCCTATTTC

712_501Mod CAAACCTAGTTGAATGAATCTGAGCCATTAAAGATATTTTAGGCATCCTATTCCTATTTC

705_501Mod CAAACCTAGTTGAATGAATCTGAGCCATTAAAGATATTTTAGGCATCCTATTCCTATTTC

711_501Mod CAAACCTAGTTGAATGAATCTGAGCCATTAAAGATATTTTAGGCATCCTATTCCTATTTC

702_508Mod CAAACCTAGTTGAATGAATCTGAGCCATTAAAGATATTTTAGGCATCCTATTCCTATTTC

Dama_mesopotamica CAAACCTAGTCGAATGAATCTGAGCTATTAAAGATATCCTAGGCATCCTGTTCCTAGTTT

Irish_Elk CAAACCTAGTCGACCCCTACTATACGATCAAAGACATTCTAGGTATTCTACTTCTAATTC

red_deer CAAACCCCTTTCATCCTTATTATACCATTAAAGATATCTTAGGCATCTTACTTCTTGTAC

****** * * * * ** ***** ** **** ** * * ** *

709_501Mod TCTTCTTAATAACACTAGTACTATTTGCACCAGACTTGCTTGGAGACCCAGACAACTACA

702_501Mod TCTTCTTAATAACACTAGTACTATTTGCACCAGACTTGCTTGGAGACCCAGACAACTACA

Eemian TCTTCTTAATAACACTAGTACTATTTGCACCAGACTTGCTTGGAGACCCAGACAACTACA

710_501Mod TCTTCTTAATAACACTAGTACTATTTGCACCAGACTTGCTTGGAGACCCAGACAACTACA

706_501Mod TCTTCTTAATAACACTAGTACTATTTGCACCAGACTTGCTTGGAGACCCAGACAACTACA

707_501Mod TCTTCTTAATAACACTAGTACTATTTGCACCAGACTTGCTTGGAGACCCAGACAACTACA

701_508Mod TCTTCTTAATAACACTAGTACTATTTGCACCAGACTTGCTTGGAGACCCAGACAACTACA

712_501Mod TCTTCTTAATAACACTAGTACTATTTGCACCAGACTTGCTTGGAGACCCAAACAACTACA

705_501Mod TCTTCTTAATAACACTAGTACTATTTGCACCAGACTTGCTTGGAGACCCAGACAACTACA

711_501Mod TCTTCTTAATAACACTAGTACTATTTGCACCAGACTTGCTTGGAGACCCAGACAACTACA

702_508Mod TCTTCTTAATAACACTAGTACTATTTGCACCAGACTTGCTTGGAGACCCAGACAACTACA

Dama_mesopotamica TCTTCTTAATATTACTAGTATTATTCGCGCCAGACTTGCTTGGAGACCCAGACAACTATA

Irish_Elk TCTTYTTAATATTACTAGTACTATTCGCACCAGACTTGCTTGGAGACCCAGACAACTACA

red_deer TCTTCTTAATATTACTAGTATTATTCGCACCAGACCTACTTGGAGATCCAGATAACTACA

**** ****** ******* **** ** ****** * ******** *** * ***** *

709_501Mod CTCCAGCAAATCCACTCAACACACCTCCTCATATTAAACCCGAATGA-------------

702_501Mod CTCCAGCAAATCCACTCAACACACCTCCTCATATTAAACCCGAATGA-------------

Eemian CTCCAGCAAATCCACTCAACACACCTCCTCATATTAAACCCGAATGA-------------

710_501Mod CTCCAGCAAATCCACTCAACACACCTCCTCATATTAAACCCGAATGA-------------

706_501Mod CTCCAGCAAATCCACTCAACACACCTCCTCATATTAAACCCGAATGA-------------

707_501Mod CTCCAGCAAATCCACTCAACACACCTCCTCATATTAAACCCGAATGA-------------

701_508Mod CTCCAGCAAATCCACTCAACACACCTCCTCATATTAAACCCGAATGA-------------

712_501Mod CTCCAGCAAATCCACTCAACACACCTCCTCATATTAAACCCGAATGA-------------

705_501Mod CTCCAGCAAATCCACTCAACACACCTCCTCATATTAAACCCGAATGA-------------

711_501Mod CTCCAGCAAATCCACTCAACACACCTCCTCATATTAAACCCGAATGA-------------

702_508Mod CTCCAGCAAATCCACTCAACACACCTCCTCATATTAAACCCGAATGA-------------

Dama_mesopotamica CCCCAGCAAATCCACTCAACACACCCCCTCATATTAAACCCGAGTGA-------------

Irish_Elk CCCCAGCAAACCCACTCAACACACCCCCTCATATTAAACCTGA-----------------

red_deer CCCCAGCAAACCCACTCAACACACCCCCTCATATTAAACCTGAATGATATTTCCCTGAAT

* ******** ************** ************** **

709_501Mod --TACTTC-----TGCATACGCAATCCTACGATCAATTCCCAATAAATTAGGAGGGGTCT

702_501Mod --TATTTC-----TGCATACGCAATCCTACGATCAATTCCCAATAAATTAGGAGGGGTCT

Eemian --TACTT------TATATACGCAATCCTACGATCAATTCCCAATAAATTAGGAGGGGTCT

710_501Mod --TACTTC-----TGCATACGCAATCCTACGATCAATTCCCAATAAATTAGGAGGGGTCT

706_501Mod --TACTTC-----TGCATACGCAATCCTACGATCAATTCCCAATAAATTAGGAGGGGTCT

707_501Mod --TATTTC-----TGCATACGCAATCCTACGATCAATTCCCAATAAATTAGGAGGGGTCT

701_508Mod --TACTTC-----TGCATACGCAATCCTACGATCAATTCCCAATAAATTAGGAGGGGTCT

712_501Mod --TACTTC-----TGCATACGCAATCCTACGATCAATTCCCAATAAATTAGGAGGGGTCT

705_501Mod --TACTTC-----TGCATACGCAATCCTACGATCAATTCCCAATAAATTAGGAGGGGTCT

711_501Mod --TACTTC-----TGCATACGCAATCCTACGATCAATTCCCAATAAATTAGGAGGGGTCT

702_508Mod --TACTTC-----TGCATACGCAATCCTACGATCAATTCCCAATAAATTAGGAGGGGTCT

Dama_mesopotamica --TATTTC-----CGCATACGCAATCTTACGATCAATCCCCAATAAACTAGGAGGAGTCT

Irish_Elk --TATTTCCTATTTGCATACGCAATCCTACGATCAATTCCCAATAAACTAGGAGGAGTCT

red_deer GATATTTCCTATTTGCATACGCAATCCTACGATCAATTCCCAACAAACTAGGAGGAGTCT

** ** ********** ********** ***** *** ******* ****

709_501Mod TAGCCCTAGTCTCATCCATCCT--GATCCTAATTCTTATACCCTTCCTCCACACATCCAA

702_501Mod TAGCCCTAGTCTCATCCATCCT--GATCCTAATTCTTATACCCTTCCTCCACACATCCAA

Eemian TAGCCCTAGTCTCATCCATCCNTAGATCCTAATTCTTATACCCTTCCTCCACACATCCAA

710_501Mod TAGCCCTAGTCTCATCCATCCT--GATCCTAATTCTTATACCCTTCCTCCACACATCCAA

706_501Mod TAGCCCTAGTCTCATCCATCCT--GATCCTAATTCTTATACCCTTCCTCCACACATCCAA

707_501Mod TAGCCCTAGTCTCATCCATCCT--GATCCTAATTCTTATACCCTTCCTCCACACATCCAA

701_508Mod TAGCCCTAGTCTCATCCATCCT--GATCCTAATTCTTATACCCTTCCTCCACACATCCAA

712_501Mod TAGCCCTAGTCTCATCCATCCT--GATCCTAATTCTTATACCCTTCCTCCACACATCCAA

705_501Mod TAGCCCTAGTCTCATCCATCCT--GATCCTAATTCTTATACCCTTCCTCCACACATCCAA

711_501Mod TAGCCCTAGTCTCATCCATCCT--GATCCTAATTCTTATACCCTTCCTCCACACATCCAA

702_508Mod TAGCCCTAGTCTCATCCATCCT--GATCCTAATTCTTATACCCTTCCTCCACACATCCAA

Dama_mesopotamica TAGCCCTAGTCTCATCTATCCT--AGTCCTAATTCTCATGCCTCTCCTTCACACATCCAA

Irish_Elk TAGCCCTAGTCTCATCCATCCT--AATCCTAATTCTGATACCTCTTCTCCATACATCTAA

red_deer TAGCCCTAATCTCATCCATCCT--AGTCTTAATTCTCATGCCTCTTCTTCACACATCCAA

******** ******* **** ** ******* ** ** * ** ** ***** **

709_501Mod ACAACGCAGCATGATATTCCGACCATTTAGTCAATGCTTATTCTGAGTCTTAGTAGCAGA

702_501Mod ACAACGCAGCATGATATTCCGACCATTTAGTCAATGCTTATTCTGAGTCTTAGTAGCAGA

Eemian ACAACGCAGCATGATATTCCGACCATTTAGTCAATGCTTATTCTGAGTCTTAGTAGCAGA

710_501Mod ACAACGCAGCATGATATTCCGACCATTTAGTCAATGCTTATTCTGAGTCTTAGTAGCAGA

706_501Mod ACAACGCAGCATGATATTCCGACCATTTAGTCAATGCTTATTCTGAGTCTTAGTAGCAGA

707_501Mod ACAACGCAGCATGATATTCCGACCATTTAGTCAATGCTTATTCTGAGTCTTAGTAGCAGA

701_508Mod ACAACGCAGCATGATATTCCGACCATTTAGTCAATGCTTATTCTGAGTCCTAGTAGCAGA

712_501Mod ACAACGCAGCATGATATTCCGACCATTTAGTCAATGCTTATTCTGAGTCTTAGTAGCAGA

705_501Mod ACAACGCAGCATGATATTCCGACCATTTAGTCAATGCTTATTCTGAGTCTTAGTAGCAGA

711_501Mod ACAACGCAGCATGATATTCCGACCATTTAGTCAATGCTTATTCTGAGTCTTAGTAGCAGA

702_508Mod ACAACGCAGCATGATATTCCGACCATTTAGTCAATGCTTATTCTGAGTCTTAGTAGCAGA

Dama_mesopotamica ACAACGTAGCATAATATTTCGACCATTCAGCCAATGCTTATTCTGAGTATTAGTAGCAGA

Irish_Elk ACAACGCAGCATGATATTCCGACCATTCAGCCAATGCTTATTCTGAATTTTAGTAGCAGA

red_deer ACAACGCAGCATGATATTCCGACCATTCAGTCAATGCCTATTCTGAATCTTAGTAGCAGA

****** ***** ***** ******** ** ****** ******** * **********

709_501Mod TCTACTAACACTTACATGAATCGGAGGACAACCAGTTGAATATCCTTTTATTACCATTGG

702_501Mod TCTACTAACACTTACATGAATCGGAGGACAACCAGTTGAATATCCTTTTATTACCATTGG

Eemian CCTACTAACACTTACATGAATCGGAGGACAACCAGTTGAATATCCTTTTATTACCATTGG

710_501Mod TCTACTAACACTTACATGAATCGGAGGACAACCAGTTGAATATCCTTTTATTACCATTGG

706_501Mod CCTACTAACACTTACATGAATCGGAGGACAACCAGTTGAATATCCTTTTATTACCATTGG

707_501Mod CCTACTAACACTTACATGAATCGGAGGACAACCAGTTGAATATCCTTTTATTACCATTGG

701_508Mod CCTACTAACACTTACATGAATCGGAGGACAACCAGTCGAATATCCTTTTATTACCATTGG

712_501Mod CCTACTAACACTTACATGAATCGGAGGACAACCAGTTGAATATCCTTTTATTACCATTGG

705_501Mod CCTACTAACACTTACATGAATCGGAGGACAACCAGTTGAATATCCTTTTATTACCATTGG

711_501Mod CCTACTAACACTTACATGAATCGGAGGACAACCAGTTGAATATCCTTTTATTACCATTGG

702_508Mod CCTATTAACACTTACATGAATCGGAGGACAACCAGTTGAATATCCTTTTATTACCATTGG

Dama_mesopotamica CCTACTAACACTCACATGAATCGGAGGACAACCAGTCGAATACCCCTTTATCATCATTGG

Irish_Elk CCTACTAACACTCACATGAATCGGAGGACAACCAGTTGAATACCCTTTTATCATCATCGG

red_deer TCTACTAACACTTACATGAATCGGAGGACAACCAGTCGAATACCCCTTTATCATTATTGG

*** ******* *********************** ***** ** ***** * ** **

709_501Mod ACAACTAGCATCTATCTTATATTTTCTCATTATTCTAGTCCTTACAATCCTACGC-TTTA

702_501Mod ACAACTAGCATCTATCTTATATTTTCTCATTATTCTAGTCCTTACAATCCTACGC-TTTA

Eemian ACAACTAGCATCTATCTTATATTTTCTCATTATTCTAGTCCT-ACAACCCTACGC-TTTA

710_501Mod ACAACTAGCATCTATCTTATATTTTCTCATTATTCTAGTCCTTACAATCCTACGC-TTTA

706_501Mod ACAACTAGCATCTATCTTATATTTTCTCATTATTCTAGTCCTTACGACCCTACGC-TTTA

707_501Mod ACAACTAGCATCTATCTTATATTTTCTCATTATTCTAGTCCTTACAATCCTACGC-TTTA

701_508Mod ACAACTAGCATCTATCTTATATTTTCTCATTATTCTAGTCCTTACAACCCTACGC-TTTA

712_501Mod ACAACTAGCATCTATCTTATATTTTCTCATTATTCTAGTCCTTACAATCCTACGC-TTTA

705_501Mod ACAACTAGCATCTATCTTATATTTTCTCATTATTCTAGTCCTTACGACCCTACGC-TTTA

711_501Mod ACAACTAGCATCTATCTTATATTTTCTCATTATTCTAGTCCTTACAACCCTACGC-TTTA

702_508Mod ACAACTAGCATCTATCTTATATTTTCTCATTATTCTAGTCCTTACAACCCTACGC-TTTA

Dama_mesopotamica ACAACTAGCATCTATCTTATATTTCCTCATTATCCTAGTCCTTATAACTTTATGCGCTTA

Irish_Elk ACAACTAGCATCTGTCCTATACTTTCTTA-TNNNNNNNNNNNNNNNNNNNNNNNNNNNNN

red_deer ACAACTAGCATCTGTCTTATATCCACACAACAAAATATGTAATAAAACCTTATGCGCTTA

************* ** **** * *

709_501Mod TAGTACATAGAATTAATGTATTAGGACATACTATGTATAATAGTACATTACATTATATAC

702_501Mod TAGTACATAGAATTAATGTATTAGGACATATTATGTATAATAGTACATTACATTATATAC

Eemian TAGTACATAGAATTAATGTATTAGGACATACTATGTATAATAGTACATTACATTATATAC

710_501Mod TAGTACATAGAATTAATGTATTAGGACATACTATGTATAATAGTACATTACATTATATAC

706_501Mod TAGTACATAGAATTAATGTATTAGGACATATTATGTATAATAGTACATTACATTATATAC

707_501Mod TAGTACATAGAATTAATGTATTAGGACATACTATGTATAATAGTACATTACATTATATAC

701_508Mod TAGTACATAGAATTAATGTATTAGGACATACTATGTATAATAGTACATTACATTATATAC

712_501Mod TAGTACATAGAATTAATGTATTAGGACATATTATGTATAATAGTACATTACATTATATAC

705_501Mod TAGTACATAGAATTAATGTATTAGGACATACTATGTATAATAGTACATTACATTATATAC

711_501Mod TAGTACATAGAATTAATGTATTAGGACATACTATGTATAATAGTACATTACATTATATAC

702_508Mod TAGTACATAGAATTAATGTATTAGGACATACTATGTATAATAGTACATTACATTATATAC

Dama_mesopotamica TAGTACATAGAATTAATGTACTAGGACATATTATGTATAATAGTACATTACATTATATAC

Irish_Elk NNNNNNNNNNAATTAATGTATTAGGACATACTATGTATAATAGTACATTATATTATATGC

red_deer TAGTACATAGAATTAATGTACTAGGACATACTATGTATAATAGTACATTATATTATATGC

********** ********* ******************* ******* *

709_501Mod CCCATGCTTATAAGCATGTACTTTTTACTGTTTACAGTACATAGTACATACCATTGTCCA

702_501Mod CCCATGCTTATAAGCATGTACTTTTCACTGTTTACAGTACATAGTACATACCATTGTCCA

Eemian CCCATGCTTATAAGCATGTACTTTTTACTGTTTACAGTACATAGTACATACTATTGTTCA

710_501Mod CCCATGCTTATAAGCATGTGCTTTTTACTGTTTACAGTACATAGTACATACCATTGTCCA

706_501Mod CCCATGCTTATAAGCATGTACTTTTTACTGTTTACAGTACATAGTACATACTATTGTCTA

707_501Mod CCCATGCTTATAAGCATGTACTTTTTACTGTTTACAGTACATAGTACATACTATTGTCTA

701_508Mod CCCATGCTTATAAGCATGTACTTTTTACTGTTTACAGTACATGGTACATATCATTGTCCA

712_501Mod CCCATGCTTATAAGCATGTACTTTTTACTGTTTACAGTACATAGTACATACTATTGTTCA

705_501Mod CCCATGCTTATAAGCATGTACTTTTTACTGTTTACAGTACATAGTACATACTATTGTCTA

711_501Mod CCCATGCTTATAAGCATGTACTTTTTACTGTTTACAGTACATAGTACATACTATTGTTCA

702_508Mod CCCATGCTTATAAGCATGTACTTTCTACTGTTTACAGTACATAGTACATACTATTGTTCG

Dama_mesopotamica CCCATGCTTATAAGCATGTATTTTCCATTATTTATAGTACATGGTACATGTTATTGTTCA

Irish_Elk CCCATGCTTATAAGCACGTATATTCCANNNNNNNNNNNNNNNNNNNNNNNNNNNNNNNNN

red_deer CCCATGCATATAAGCATGTACTTTCTATTATTTATAGTACATAGTACATGATGTTGTTCA

******* ******** ** ** *

709_501Mod TCGTACATAGCACATTGAGTCAAATCAATTCTCGTCAA

702_501Mod TCGTACATGGCACATTAAGTCAAATCAATTCTCGTCAA

Eemian TCGTACATAGCACATTAAGTCAAATCAATTCTCGTCA-

710_501Mod TCGTACATAGCACATTAAGTCAAATCAATTCTCGTCAA

706_501Mod TCGTACATAGCACATTAGGTCAAATCAATTCTCGCCAA

707_501Mod TCGTACATAGCACATTAAGTCAAATCAATTCTCGTCAA

701_508Mod TCGTACATAGCACATTAGGTCAAATCAATTCTCGTCAA

712_501Mod TCGTACATAGCACATTAAGTCAAATCAATTCTTGTCAA

705_501Mod TCGTACATAGCACATTAGGTCAAATCAATTCTCGCCAA

711_501Mod TCGTACATAGCACATTAAGTCAAATCAATTCTTGTCAA

702_508Mod TCGTACATAGCACATTAAGTCAAATCAATTCTTGTCAA

Dama_mesopotamica TCGTACATAGCACATTGAGTCAAATCAGTTCTTGTCA-

Irish_Elk NNNNNNNNNNNNNNNNNAGTCAAAT-------------

red_deer TCGTACATAGCGCATTA---------------------
